# Body Roundness Index and Waist–Hip Ratio Result in Better Cardiovascular Disease Risk Stratification: Results From a Large Chinese Cross-Sectional Study

**DOI:** 10.3389/fnut.2022.801582

**Published:** 2022-03-10

**Authors:** Ying Li, Yongmei He, Lin Yang, Qingqi Liu, Chao Li, Yaqin Wang, Pingting Yang, Jiangang Wang, Zhiheng Chen, Xin Huang

**Affiliations:** ^1^Department of Health Management, The Third Xiangya Hospital, Central South University, Changsha, China; ^2^Department of Health Management, Aerospace Center Hospital, Beijing, China; ^3^Department of Cancer Epidemiology and Prevention Research, Cancer Care Alberta, Alberta Health Services, Calgary, AB, Canada; ^4^Departments of Oncology and Community Health Sciences, Cumming School of Medicine, University of Calgary, Calgary, AB, Canada; ^5^Department of Biostatistics, Bioinformatics and Biomathematics, Georgetown University, Washington, DC, United States; ^6^Hunan Key Laboratory for Bioanalysis of Complex Matrix Samples, Changsha, China; ^7^Department of Epidemiology, School of Medicine, Hunan Normal University, Changsha, China

**Keywords:** waist circumference, hip circumference, ABSI, BRI, WHR, WHtR, cardiovascular disease risk factors

## Abstract

**Background:**

The appropriate optimal anthropometric indices and their thresholds within each BMI category for predicting those at a high risk of cardiovascular disease risk factors (CVDRFs) among the Chinese are still under dispute.

**Objectives:**

We aimed to identify the best indicators of CVDRFs and the optimal threshold within each BMI category among the Chinese.

**Methods:**

Between 2012 and 2020, a total of 500,090 participants were surveyed in Hunan, China. Six anthropometric indices including waist circumference (WC), a body shape index (ABSI), body roundness index (BRI), waist–hip ratio (WHR), hip circumference (HC), and waist–height ratio (WHtR) were evaluated in the present study. Considered CVDRFs included dyslipidaemia, hypertension, diabetes mellitus (DM), and chronic kidney disease (CKD). The associations of anthropometrics with CVDRFs within each BMI category were evaluated through logistic regression models. The area under the receiver operating characteristic curve (AUROC) was used to assess the predictive abilities.

**Results:**

For the presence of at least one CVDRFs, the WHR had the highest AUROC in overweight [0.641 (95%CI:0.638, 0.644)] and obese [0.616 (95%CI:0.609, 0.623)] men. BRI had the highest AUROC in underweight [0.649 (95%CI:0.629, 0.670)] and normal weight [0.686 (95%CI:0.683, 0.690)] men. However, the BRI had the highest discrimination ability among women in all the BMI categories, with AUROC ranging from 0.641 to 0.727. In most cases, the discriminatory ability of WHtR was similar to BRI and was easier to calculate; therefore, thresholds of BRI, WHR, and WHtR for CVDRFs identification were all calculated. In men, BRI thresholds of 1.8, 3.0, 3.9, and 5.0, WHtR thresholds of 0.41, 0.48, 0.53, and 0.58, and WHR thresholds of 0.81, 0.88, 0.92, and 0.95 were identified as optimal thresholds across underweight, normal weight, overweight, and obese populations, respectively. The corresponding BRI values in women were 1.9, 2.9, 4.0, and 5.2, respectively, and WHtR were 0.41, 0.48, 0.54, and 0.59, while the WHR values were 0.77, 0.83, 0.88, and 0.90. The recommended BRI, WHtR, or WHR cut-offs could not statistically differentiate high-risk CKD or hypercholesterolemia populations.

**Conclusions:**

We found that BRI and WHR were superior to other indices for predicting CVD risk factors, except CKD or hypercholesterolemia, among the Chinese.

## Introduction

Obesity is one of the most serious public health problems in the world. Obesity and adiposity undoubtedly increase the risk of chronic diseases, such as hypertension, diabetes, coronary heart disease, liver cirrhosis, certain types of cancer, poor mental health, and premature death (1–7). There is an unprecedented interest in discovering useful indicators of obesity and adiposity to identify chronic disease risk. As anthropometric measures are simple, inexpensive, and noninvasive tools to assess body weight and shape, a number of studies have focused on proposing better measurement and calculation methods to predict chronic disease risk and mortality (8–10).

Body mass index (BMI), defined as weight in kilograms divided by height in meters squared (kg/m^2^), has been the most widely adopted weight-related anthropometric measure in the past decade. However, the predictive ability of BMI is limited, as it does not differentiate fat from lean mass or consider the distribution of adipose tissue (11, 12). In recent years, several alternative anthropometric adiposity measurements, such as waist circumference (WC), hip circumstance (HC), waist–height ratio (WHtR), and waist–hip ratio (WHR), which focus on abdominal adiposity, have been identified as useful weight-related anthropometric measures to predict the risk of type 2 diabetes (13), cardiovascular disease (CVD) (14, 15), and all-cause mortality (16). Along with the effort of designing and testing alternative anthropometric measures, two new indices that standardize WC for height and BMI, known as the body shape index (ABSI) and body roundness index (BRI), have been proposed. Previous studies indicated that BRI is a good predictor of metabolic syndrome in both sexes in populations of various nationalities and ethnic groups, including the Chinese population (17). BRI and ABSI have a discriminatory power for hypertension in adult women and men from different populations, although BRI was a significantly better predictor of hypertension than ABSI (18). Unfortunately, until now, the best indicator of these diseases has remained unclear, especially in the Chinese population.

Furthermore, at present, there is no evidence to suggest that the current WC thresholds are the optimal cut-offs within a given BMI category. Indeed, these cut-offs may not provide adequate discrimination of CVD risk. For example, few normal weight individuals have WC values above the cut-offs, whereas almost all obese individuals have WC values exceeding these thresholds (19, 20). Moreover, a study indicated that BMI category-specific WC thresholds may refine the severe liver disease risk more accurately than traditional thresholds in UK (21). Thus, the International Atherosclerosis Society (IAS) and International Chair on Cardiometabolic Risk (ICCR) Working Group on Visceral Obesity suggested refining WC threshold values for a given BMI category in 2020 (22). With the exception of WC, some studies have illustrated the association between other adiposity anthropometric indices and chronic disease risk in each BMI category. An Iranian study indicated that WHtR has a different ability to predict hypertension in each BMI category (23). Meanwhile, ABSI achieves better mortality risk stratification of abdominal obesity in different BMI categories in European populations, which suggests complementary BMI and enables efficient risk stratification, which could facilitate personalization of screening, treatment, and monitoring (24). However, with the exception of WC, insufficient evidence has illustrated the association between other adiposity anthropometric indices and chronic disease risk in each BMI category.

This study focused on comparing the predictive value of six anthropometric indices for cardiovascular disease risk factors (CVDRFs) and then identified the best indicators of CVDRFs in each BMI category in a large-scale population. Furthermore, we aimed to identify the optimal threshold within each BMI category for predicting those at a high risk of CVDRFs in the Chinese population.

## Methods

### Study Population

Between 2012 and 2020, more than 0.7 million individuals from a mixed urban and rural area who visited health management centers for annual health check-ups in Hunan were enrolled in our cross-sectional study. The participants had a diverse socioeconomic background (public services employees, workers, self-employed persons, farmers, and others) and signed informed consent forms. Our survey was approved by the Ethics Committee of the Third Xiangya Hospital.

**Figure 1:**
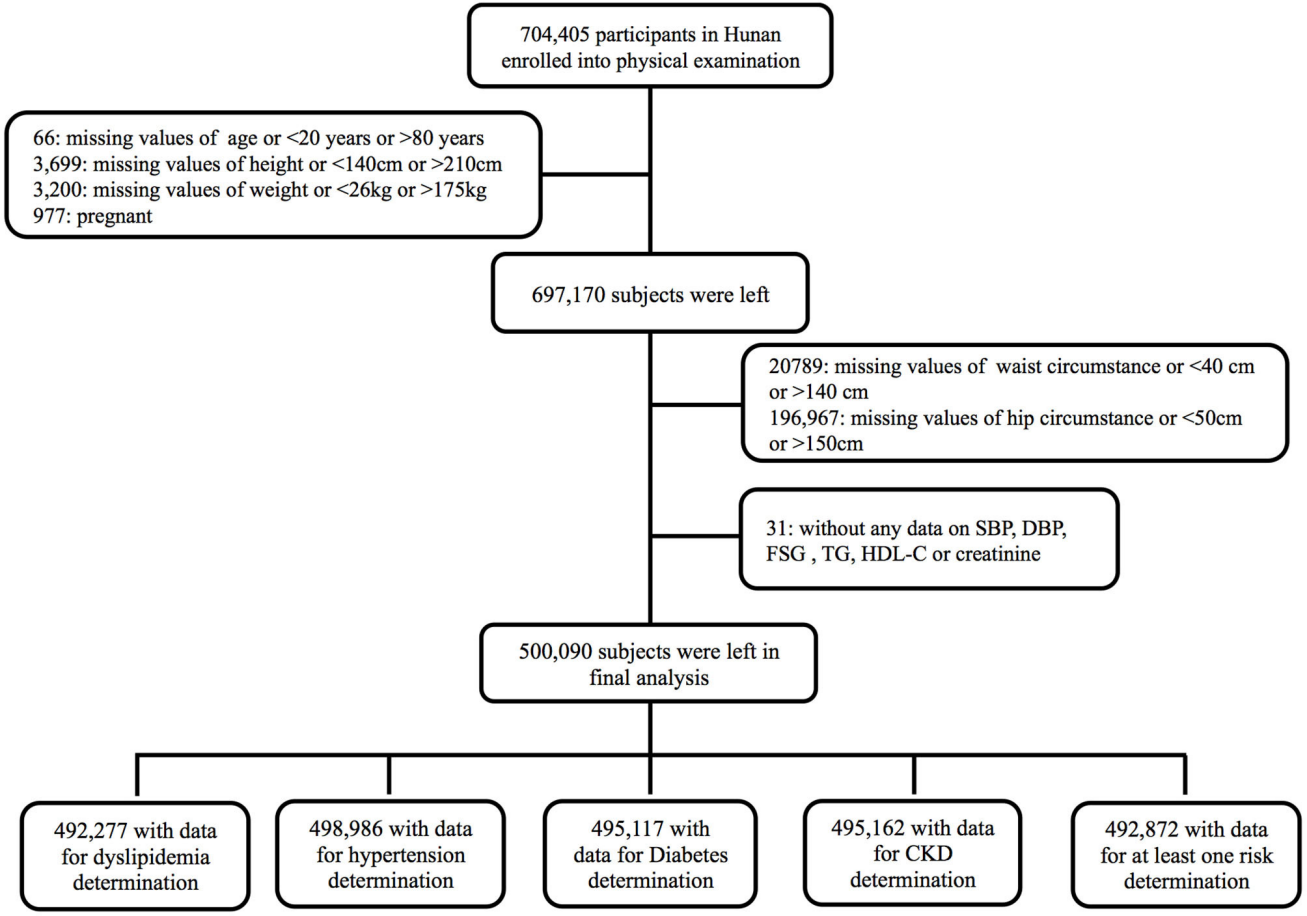
Enrollment flowchart.

All enrolled participants had undergone a routine clinical examination and completed questionnaires. Age, sex, smoking history, alcohol consumption, current medication use, and previous medical diagnoses were recorded. Individuals with missing data or unreasonable values for age, height, weight, WC, or hip circumference (HC) were excluded. Those without any data on blood pressure, serum glucose, lipids, or creatinine were further excluded. Finally, a total of 500,090 participants were left for analysis (Figure 1).

### Anthropometric Measurements and Indices

Anthropometric measurements, including body weight (Wt), height (Ht), WC, and HC, were obtained by trained physicians. Body weight (kg) and height (m) were measured with the individuals barefooted and wearing light clothes using an intelligent ultrasonic height and weight meter (SK-X80, Shuangjia Limited, Shanghai, China) with a resolution of 0.1 kg and 0.001 m. WC was measured midway between the lower rib margin and the iliac crest (25, 26). The HC was measured in the maximum circumference of the buttocks. In addition to BMI, an index of general adiposity, other anthropometric indices considered in our analyses included four abdominal adiposity indices (WC, ABSI, BRI, and WHR) and two gluteofemoral adiposity indices (HC and WHtR). The calculation of the indices is described below, with the relevant reference (27, 28) cited at the end of each formula:


$$\begin{array}{l} \text{ABSI}(28)=1,000^{*}{\text{WC}}^{\text{*}}{\text{Wt}}^{-2/3*}Ht^{5/6} \end{array}$$

$$\begin{array}{l} {\text{BMI=Wt/Ht}}^{\text{2}} \end{array}$$

$$\text{BRI}\left(28\right)\text{=364}.\text{2}-\text{365}.{\text{5}}^{*}{\left(\text{1}-({\left(\text{0}.{\text{5}}^{*}\text{WC/}\pi \right)}^{\text{2}}\text{/}{\left(\text{0}.{\text{5}}^{*}\text{Ht}\right)}^{\text{2}})\right)}^{\text{0}.\text{5}}$$

$$\begin{array}{l} \text{WHR}=\text{WC}/\text{HC} \end{array}$$

$$\begin{array}{l} \text{WHtR}=\text{WC}/\text{Ht} \end{array}$$

In the above formula, Ht, WC, and HC are in meters, and Wt is in kilograms. The original ABSI values (27) are <0.1, and in the present study, the ABSI was multiplied by 1,000, resulting in a number of WC orders of magnitude, which would be more intuitive to use than the original values.

### Definition of CVDRFs

The CVDRFs considered in our study included dyslipidaemia, hypertension, diabetes mellitus (DM), and chronic kidney disease (CKD). Dyslipidaemia was defined as serum total cholesterol (TC) >6.22 mmol/L (240 mg/dL), total triglycerides (TGs) >1.69 mmol/L (150 mg/dL), and/or the use of lipid-lowering medications. Dyslipidaemia was further classified into three subtypes: hypercholesterolemia (elevated TC and normal TGs) and hypertriglyceridemia [elevated TGs, normal TC, and mixed hyperlipidaemia (elevated TGs and TC)] (29). Hypertension was defined as elevated blood pressure (systolic blood pressure (SBP) ≥140 mmHg or diastolic blood pressure (DBP) ≥ 90 mmHg) or the use of antihypertensive medications (30). DM was defined as fasting plasma glucose (FPG) ≥ 7 mmol/L (126 mg/dL) and/or treatment for diabetes (31). CKD was defined as kidney damage (albumin-to-creatinine ratio >30 mg/g) or estimated glomerular filtration rate (eGFR) (based on CKD-EPI equation) <60 mL/min/1.73 m^2^ (32, 33).

### Statistical Analysis

According to BMI value, participants were categorized into four categories: underweight (<18.5 kg/m^2^), normal weight (18.5– <24 kg/m^2^), overweight (24– <28 kg/m^2^), and obese (≥28 kg/m^2^). We analyzed the data by sex and BMI category. Continuous variables are expressed as the means and standard deviations (SDs), categorical variables are expressed as the number of cases and percentages, and differences among different BMI groups were tested by analysis of variance (ANOVA) and the Chi-square test. The Cochran–Armitage linear trend test will be further performed when prevalence shows a trend association with BMI. The correlations between obesity indices were examined with partial Pearson correlation coefficients (r), adjusted for age. To explore whether establishing an anthropometric index for each category of BMI could enable better CVDRFs recognition, we compared anthropometric indices in three steps, as described below. First, the exposure level of each anthropometric index was classified into quartiles < Q1, Q1– < Q2, Q2– < Q3, and ≥Q3. The odds ratios (ORs) and their 95% confidence intervals (CIs) of the presence of CVDRFs according to higher anthropometric index exposure compared with the lowest quartile of each anthropometric index were calculated within each BMI category by multivariate logistic regression models controlling for age, smoking, and alcohol consumption status. Second, we used the area under the receiver operating characteristic curve (AUROC) and 95% CIs to assess the capacity of each anthropometric measure to identify CVDRFs, and the Delong test was used to compare the area under different index curves. Third, the indices with the highest and most significant AUROCs for identifying most of the CVD risk factors were selected, and the optimal cut-off value for each CVD risk factor was determined by Youden's index. After additionally considering the distribution of the selected index, only one cut-off value for each sex and BMI category was determined as the recommended value. Finally, adjusted ORs were calculated for high index exposure and risk of CVDRFs within each BMI category using logistic regression models as described for the first step above by categorizing participants with a high index (≥ recommended cut-off) as having exposure and with a low index (< recommended cut-off) as without exposure. A two-sided *p* < 0.05 was considered statistically significant. SAS version 9.4 (SAS Institute Inc) was used for analyses.

## Results

### General Characteristics of Study Participants

**Figure 2:**
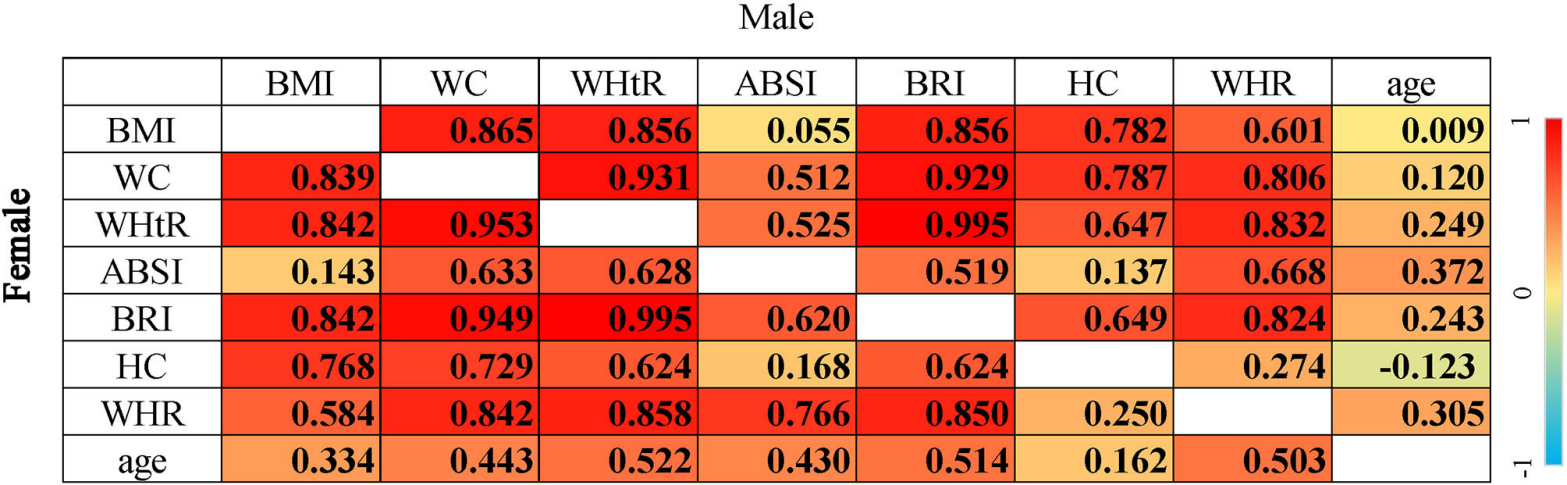
Correlation between anthropometric indices among Chinese. The *p*-value in all cells was < 0.001; correlations between each of the two anthropometric indices were partial Pearson correlation with adjustment for age, and correlations between age and each of the anthropometric indices were Pearson correlation; BMI, body mass index; WC, waist circumstance; WHtR, waist height ratio; ABSI, a body shape index; BRI, body round index; HC, hip circumstance; WHR, waist hip ratio.

A total of 500,090 participants aged 20–99 who met our inclusion criteria between 2012 and 2020 in Hunan were enrolled in our study. Participant characteristics and waist and hip indices are summarized by sex and BMI category in Table 1. There were 6,813; 1,104; 4,973; 4,928; and 7,218 subjects without enough data for the determination of dyslipidaemia, hypertension, DM, CKD, and at least one risk factor, respectively (Figure 1). The mean BMI was 24.99 ± 3.13 kg/m^2^ for men and 22.49 ± 3.00 kg/m^2^ for women. Only 3.78% men and 7.81% women had WCs above the WHO cut-offs (102 cm for men and 88 cm for women). WHR was above the high-risk WHO cut-offs (0.90 for men; 0.85 for women) in 54.46% of men and only 33.01% of women. Participants within the higher BMI category were more likely to have hypertriglyceridemia, mixed hyperlipidaemia, dyslipidaemia, hypertension, DM, and at least one CVDRF (*P*_fortrend_ <0.001). However, among men, those with a higher BMI were less likely to have CKD (*P*_fortrend_ < 0.001). The distribution of anthropometric indices by sex and BMI category among the Hunan Chinese participants is shown in Supplementary Figure S1. The correlations between anthropometric indices are listed in Figure 2. Briefly, the abdominal adiposity indices, except the ABSI (*r* < 0.15), were strongly correlated with BMI (*r* > 0.75). The two gluteofemoral adiposity indices, HC and WHR, were strongly (r > 0.75) and moderately (r ≈ 0.60) correlated with BMI, respectively. In women, age had a significant moderate to weak positive correlation with anthropometric indices (r between 0.16 and 0.52, *p* < 0.001); however, in men, the correlation became weaker (r between 0.01 and 0.37, *p* < 0.001), and the HC became a weak negative correlation (r = −0.12, *p* < 0.001).

**Table 1:** Characteristics of selected participants by different gender and BMI category.

**Characteristics**	**Overall**	**Underweight**	**Normal weight**	**Overweight**	**Obese**	P^*^
	**(Mean ±SD)/*n* (%)**	**,Mean ±SD)/*n* (%)**	**(Mean ±SD)/*n* (%)**	**(Mean ±SD)/*n* (%)**	**(Mean ±SD)/*n* (%)**	
**Male**						
Age, year	45.65 ± 13.80	42.71 ± 18.75	45.10 ± 14.99	46.49 ± 13.05	44.63 ± 12.34	<0.001
20–39 year	106,661 (37.46)	2,543 (57.85)	42,684 (42.30)	44,527 (33.09)	16,907 (37.71)	<0.001
40–59 year	133,105 (46.75)	864 (19.65)	40,163 (42.30)	69,095 (51.35)	22,983 (51.26)	
≥60 year	44,952 (15.79)	989 (22.50)	18,067 (17.90)	20,946 (15.57)	4,950 (11.04)	
Smoke	106,282 (37.33)	1,870 (42.54)	37,072 (36.74)	48,919 (36.35)	18,421 (41.08)	<0.001
Drink	116,962 (41.08)	1,365 (31.05)	37,523 (37.18)	57,591 (42.8)	20,483 (45.68)	<0.001
BMI, kg/m^2^	24.99 ± 3.13	17.57 ± 0.75	22.08 ± 1.39	25.77 ± 1.11	29.91 ± 1.92	<0.001
WC, cm	86.27 ± 8.58	67.61 ± 4.36	79.33 ± 5.58	88.32 ± 4.95	97.57 ± 6.21	<0.001
WHtR	0.51 ± 0.05	0.40 ± 0.03	0.47 ± 0.03	0.52 ± 0.03	0.58 ± 0.04	<0.001
ABSI	77.88 ± 3.70	77.02 ± 4.45	77.65 ± 3.96	78.03 ± 3.55	78.00 ± 3.42	<0.001
BRI	3.64 ± 0.99	1.70 ± 0.41	2.85 ± 0.60	3.85 ± 0.60	4.98 ± 0.81	<0.001
HC, cm	95.79 ± 5.79	84.83 ± 3.65	91.66 ± 4.02	96.84 ± 3.94	102.99 ± 4.86	<0.001
WHR	0.90 ± 0.06	0.80 ± 0.05	0.87 ± 0.05	0.91 ± 0.05	0.95 ± 0.05	<0.001
Hypercholesterolemia	10,406 (3.69)	133 (3.10)	4,180 (4.20)	4,881 (3.66)	1,212 (2.73)	<0.001
Hypertriglyceridemia	103,874 (36.88)	277 (6.46)	24,113 (24.21)	55,997 (42.00)	23,487 (52.86)	<0.001
Mixed hyperlipidemia	25,532 (9.07)	35 (0.82)	4,985 (5.01)	13,889 (10.42)	6,623 (14.91)	<0.001
Dyslipidemia	139,812 (49.64)	445 (10.37)	33,278 (33.42)	74,767 (56.08)	31,322 (70.49)	<0.001
Hypertension	68,781 (24.20)	386 (8.80)	15,731 (15.61)	35,751 (26.61)	16,913 (37.79)	<0.001
Diabetic mellitus	23,245 (8.23)	87 (2.01)	5,641 (5.64)	11,835 (8.85)	5,682 (12.76)	<0.001
Chronic kidney disease	5,725 (2.03)	117 (2.70)	2,063 (2.06)	2,747 (2.05)	798 (1.79)	<0.001
With at least one risk	174,428 (61.93)	879 (20.53)	45,064 (45.28)	91,761 (68.82)	36,724 (82.52)	<0.001
**Female**						
Age, year	42.92 ± 13.21	33.79 ± 11.88	41.42 ± 12.53	48.37 ± 12.85	49.44 ± 13.47	<0.001
20–39 year	97,618 (45.33)	10,504 (79.70)	71,403 (49.93)	12,964 (26.69)	2,747 (25.84)	<0.001
40–59 year	91,643 (42.55)	1,952 (14.81)	58,306 (40.78)	25,983 (53.49)	5,402 (50.82)	
≥60 year	26,111 (12.12)	723 (5.49)	13,284 (9.29)	9,624 (19.81)	2,480 (23.33)	
Smoke	3,490 (1.62)	286 (2.17)	2,122 (1.48)	808 (1.66)	274 (2.58)	<0.001
Drink	15,222 (7.07)	842 (6.39)	10,091 (7.06)	3,474 (7.15)	815 (7.67)	<0.001
BMI, kg/m^2^	22.49 ± 3.00	17.60 ± 0.73	21.37 ± 1.46	25.48 ± 1.08	29.99 ± 2.02	<0.001
WC, cm	74.99 ± 8.38	63.77 ± 4.17	72.29 ± 5.63	82.24 ± 5.50	92.15 ± 6.93	<0.001
WHtR	0.48 ± 0.06	0.40 ± 0.03	0.46 ± 0.04	0.53 ± 0.04	0.59 ± 0.05	<0.001
ABSI	75.12 ± 4.51	74.69 ± 4.46	74.76 ± 4.46	76.00 ± 4.49	76.52 ± 4.58	<0.001
BRI	2.99 ± 1.06	1.69 ± 0.41	2.64 ± 0.66	3.90 ± 0.75	5.29 ± 1.06	<0.001
HC, cm	91.36 ± 5.56	84.32 ± 3.48	89.81 ± 3.96	95.42 ± 4.14	102.44 ± 5.46	<0.001
WHR	0.82 ± 0.06	0.76 ± 0.05	0.81 ± 0.06	0.86 ± 0.06	0.90 ± 0.06	<0.001
Hypercholesterolemia	12,325 (5.82)	377 (2.98)	7,939 (5.65)	3,352 (6.98)	657 (6.25)	<0.001
Hypertriglyceridemia	27,496 (12.99)	297 (2.35)	12,759 (9.08)	11,068 (23.05)	3,372 (32.06)	<0.001
Mixed hyperlipidemia	9,052 (4.28)	57 (0.45)	4,030 (2.87)	3,777 (7.87)	1,188 (11.3)	<0.001
Dyslipidemia	48,873 (23.09)	731 (5.77)	24,728 (17.61)	18,197 (37.90)	5,217 (49.61)	<0.001
Hypertension	25,384 (11.82)	371 (2.82)	10,822 (7.59)	10,468 (21.62)	3,723 (35.13)	<0.001
Diabetic mellitus	6,697 (3.15)	95 (0.74)	2,547 (1.81)	2,874 (5.97)	1,181 (11.21)	<0.001
Chronic kidney disease	1,708 (0.80)	58 (0.45)	804 (0.57)	646 (1.34)	200 (1.90)	<0.001
With at least one risk	63,534 (30.08)	1,066 (8.45)	31,945 (22.8)	23,572 (49.13)	6,951 (65.99)	<0.001

* *Differences among different BMI groups were tested by analysis of variance (ANOVA) for continuous variables and the Chi-square test for binary variables. BMI, body mass index; WC, waist circumstance; WHtR, waist height ratio; ABSI, a body shape index; BRI, body round index; HC, hip circumstance; WHR, waist hip ratio*.

### Associations of Six Anthropometric Indices With CVDRFs

**Figure 3:**
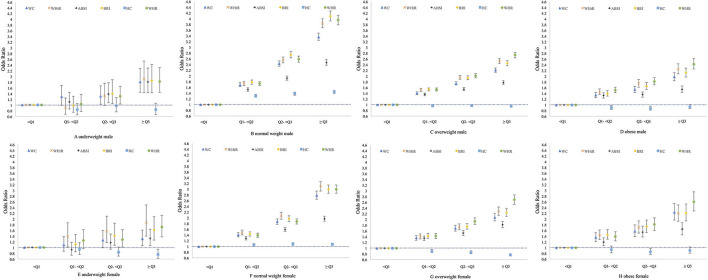
Association between with at least one CVD risk factor and six anthropometric indices among Chinese. Association was adjusted for age, smoking, and drinking status and index at < Q1 level was treated as non-exposed; Q1, first quartile, the 25th percentile; Q2: second quartile, median; Q3, third quartile, the 75th percentile; WC, waist circumstance; WHtR, waist height ratio; ABSI, a body shape index; BRI, body round index; HC, hip circumstance; WHR, waist hip ratio.

Figure 3 and Supplementary Figures S2–S5 show the associations of six anthropometric indices with at least one CVDRF, and each component of CVDRFs studied in the present research by sex and BMI category. Basically, among all the BMI categories, linear positive associations between WC, WHtR, ABSI, BRI, and WHR exposure and dyslipidaemia, hypertension, DM, and at least one CVDRF were observed. CKD was significantly associated with these five indices only among men with normal weight.

The ORs of HC exposure for risk of CVDRFs varied between different sex and BMI categories. Among obese individuals, HC exhibited a “U” -shaped association with dyslipidaemia, hypertension, diabetes, and at least one CVDRF. Among the overweight and underweight individuals, the higher the HC, the lower the risk of hypertension, DM, and presence of at least one CVDRF. In the normal-weight group, increasing trends were observed in the association between HC and dyslipidaemia and the presence of at least one CVDRF.

The subtypes of dyslipidaemia showed different associations with anthropometric indices by BMI category. Hypertriglyceridemia was linearly positively associated with WC, WHtR, ABSI, BRI, and WHR exposure among the overall sample, regardless of BMI. Mixed hyperlipidaemia showed the same positive linear trends with those five indices among the normal weight, overweight, and obese groups. However, among underweight individuals, “U” -shaped associations were found for mixed hyperlipidaemia and the five exposure indices. For the hypercholesterolemia subtype, the higher the WC, WHtR, ABSI, or BRI, the lower the risk among overweight individuals. Among the normal weight and overweight groups, a nonlinear association was found. Moreover, among underweight women, hypercholesterolemia was positively associated with WHtR and BRI exposure.

### ROC Curve of Six Anthropometric Indices for the Identification of CVDRFs

To identify the index with the best discriminatory ability for CVDRFs identification, we compared all the AUROCs of different indicators within different sex and BMI categories (Table 2). The capabilities of six anthropometric indices for identifying each CVDRFs were significantly different (*p* < 0.05). Generally, the identifying capabilities for each CVDRFs component among overweight and obese individuals were lower than those among normal or underweight individuals. In addition, the BRI and WHtR had similar discrimination abilities, and they alternately had the highest AUROC values with WHR in identifying CVDRFs.

**Table 2:** Capacity for identifying CVD associated risk factors by using waist and hip indices within each BMI category among Chinese.

**Index**	**Area under ROC curve (95%CI)**							
	Hypercholest-erolemia^*^	Hypertrigly-ceridemia^*^	Mixed hyperlipidemia^*^	Dyslipidemia^*^	Hypertension^*^	Diabetic mellitus^*^	Chronic kidney disease^*^	With at least one CVD risk^*^
**Male (*****N*** **=** **284, 718)**								
Underweight								
WC	0.555 (0.503, 0.607)	0.608 (0.574, 0.642)	0.675 (0.593, 0.756)	0.603 (0.575, 0.631)	0.538 (0.507, 0.569)	0.579 (0.518, 0.640)	0.522 (0.467, 0.576)	0.570 (0.548, 0.592)
WHtR	0.605 (0.551, 0.659)	0.615 (0.581, 0.648)	0.669 (0.580, 0.757)	0.623 (0.595, 0.651)	0.658 (0.630, 0.687)	0.688 (0.633, 0.744)	0.666 (0.614, 0.719)	0.649 (0.629, 0.670)
ABSI	0.600 (0.548, 0.653)	0.608 (0.574, 0.643)	0.686 (0.601, 0.771)	0.619 (0.591, 0.647)	0.617 (0.588, 0.646)	0.634 (0.573, 0.695)	0.642 (0.592, 0.691)	0.628 (0.607, 0.649)
BRI	0.607 (0.553, 0.661)	0.616 (0.582, 0.650)	0.671 (0.582, 0.760)	0.625 (0.597, 0.653)	0.657 (0.629, 0.686)	0.689 (0.634, 0.744)	0.669 (0.617, 0.722)	0.649 (0.629, 0.670)
HC	0.499 (0.447, 0.551)	0.512 (0.476, 0.548)	0.597 (0.509, 0.686)	0.516 (0.487, 0.545)	0.559 (0.528, 0.590)	0.587 (0.528, 0.646)	0.617 (0.567, 0.666)	0.549 (0.527, 0.570)
WHR	0.548 (0.493, 0.602)	0.627 (0.593, 0.662)	0.756 (0.675, 0.837)	0.620 (0.592, 0.649)	0.590 (0.559, 0.620)	0.637 (0.577, 0.697)	0.616 (0.564, 0.669)	0.611 (0.590, 0.633)
Normal weight								
WC	0.517 (0.508, 0.525)	0.638 (0.635, 0.642)	0.652 (0.644, 0.659)	0.649 (0.646, 0.653)	0.602 (0.597, 0.607)	0.659 (0.652, 0.666)	0.583 (0.570, 0.596)	0.655 (0.652, 0.659)
WHtR	0.556 (0.548, 0.565)	0.630 (0.626, 0.633)	0.668 (0.661, 0.675)	0.653 (0.650, 0.656)	0.662 (0.658, 0.667)	0.703 (0.696, 0.709)	0.687 (0.675, 0.699)	0.685 (0.682, 0.689)
ABSI	0.555 (0.546, 0.563)	0.593 (0.589, 0.597)	0.628 (0.621, 0.636)	0.614 (0.610, 0.618)	0.627 (0.622, 0.631)	0.689 (0.683, 0.696)	0.674 (0.663, 0.686)	0.644 (0.640, 0.647)
BRI	0.557 (0.549, 0.566)	0.630 (0.626, 0.634)	0.669 (0.662, 0.676)	0.654 (0.650, 0.657)	0.663 (0.658, 0.668)	0.704 (0.697, 0.710)	0.688 (0.676, 0.700)	0.686 (0.683, 0.690)
HC	0.540 (0.532, 0.549)	0.545 (0.541, 0.549)	0.518 (0.510, 0.526)	0.534 (0.530, 0.537)	0.549 (0.544, 0.553)	0.555 (0.548, 0.563)	0.593 (0.581, 0.605)	0.503 (0.499, 0.506)
WHR	0.550 (0.541, 0.559)	0.633 (0.629, 0.637)	0.667 (0.659, 0.674)	0.655 (0.651, 0.658)	0.656 (0.651, 0.660)	0.727 (0.721, 0.734)	0.663 (0.651, 0.675)	0.685 (0.681, 0.688)
Overweight								
WC	0.514 (0.506, 0.523)	0.553 (0.550, 0.556)	0.567 (0.562, 0.572)	0.576 (0.573, 0.579)	0.579 (0.576, 0.583)	0.612 (0.606, 0.617)	0.594 (0.583, 0.605)	0.607 (0.604, 0.610)
WHtR	0.527 (0.519, 0.535)	0.540 (0.537, 0.543)	0.573 (0.569, 0.578)	0.571 (0.568, 0.574)	0.629 (0.626, 0.632)	0.656 (0.651, 0.661)	0.689 (0.679, 0.699)	0.629 (0.626, 0.633)
ABSI	0.522 (0.514, 0.530)	0.527 (0.524, 0.530)	0.561 (0.556, 0.566)	0.553 (0.550, 0.557)	0.597 (0.594, 0.600)	0.651 (0.646, 0.656)	0.685 (0.674, 0.695)	0.603 (0.600, 0.606)
BRI	0.527 (0.519, 0.535)	0.540 (0.537, 0.543)	0.574 (0.569, 0.579)	0.572 (0.569, 0.575)	0.630 (0.626, 0.633)	0.657 (0.652, 0.662)	0.690 (0.680, 0.700)	0.630 (0.627, 0.633)
HC	0.537 (0.529, 0.545)	0.507 (0.504, 0.510)	0.512 (0.507, 0.517)	0.503 (0.500, 0.506)	0.547 (0.544, 0.551)	0.573 (0.568, 0.579)	0.578 (0.567, 0.589)	0.529 (0.525, 0.532)
WHR	0.512 (0.504, 0.520)	0.553 (0.550, 0.556)	0.584 (0.579, 0.589)	0.586 (0.583, 0.589)	0.626 (0.623, 0.630)	0.683 (0.678, 0.688)	0.662 (0.652, 0.673)	0.641 (0.638, 0.644)
Obese								
WC	0.513 (0.497, 0.529)	0.531 (0.526, 0.537)	0.523 (0.515, 0.530)	0.550 (0.544, 0.555)	0.563 (0.557, 0.568)	0.596 (0.589, 0.604)	0.561 (0.540, 0.581)	0.580 (0.573, 0.587)
WHtR	0.534 (0.518, 0.550)	0.513 (0.508, 0.518)	0.533 (0.525, 0.540)	0.540 (0.534, 0.546)	0.605 (0.599, 0.610)	0.631 (0.623, 0.638)	0.664 (0.645, 0.682)	0.601 (0.594, 0.608)
ABSI	0.543 (0.526, 0.559)	0.500 (0.495, 0.506)	0.521 (0.514, 0.529)	0.519 (0.513, 0.525)	0.576 (0.571, 0.581)	0.621 (0.613, 0.628)	0.658 (0.638, 0.678)	0.572 (0.565, 0.579)
BRI	0.534 (0.518, 0.550)	0.513 (0.508, 0.519)	0.533 (0.525, 0.540)	0.540 (0.534, 0.546)	0.605 (0.600, 0.611)	0.631 (0.624, 0.639)	0.664 (0.645, 0.682)	0.601 (0.594, 0.608)
HC	0.528 (0.512, 0.544)	0.513 (0.508, 0.519)	0.518 (0.510, 0.525)	0.502 (0.496, 0.508)	0.528 (0.522, 0.533)	0.532 (0.524, 0.540)	0.555 (0.535, 0.575)	0.522 (0.516, 0.529)
WHR	0.505 (0.488, 0.521)	0.526 (0.521, 0.531)	0.544 (0.536, 0.551)	0.558 (0.552, 0.564)	0.601 (0.596, 0.607)	0.646 (0.639, 0.654)	0.613 (0.592, 0.633)	0.616 (0.609, 0.623)
**Female (*****N*** **=** **215, 372)**								
Underweight								
WC	0.569 (0.540, 0.598)	0.615 (0.581, 0.649)	0.651 (0.575, 0.728)	0.598 (0.576, 0.619)	0.592 (0.560, 0.623)	0.657 (0.598, 0.716)	0.552 (0.475, 0.628)	0.591 (0.573, 0.609)
WHtR	0.632 (0.604, 0.660)	0.658 (0.626, 0.690)	0.760 (0.690, 0.831)	0.658 (0.637, 0.679)	0.703 (0.674, 0.732)	0.746 (0.692, 0.800)	0.696 (0.627, 0.765)	0.668 (0.650, 0.685)
ABSI	0.617 (0.588, 0.646)	0.627 (0.593, 0.661)	0.715 (0.640, 0.789)	0.633 (0.612, 0.655)	0.693 (0.664, 0.721)	0.742 (0.687, 0.797)	0.712 (0.646, 0.778)	0.648 (0.630, 0.666)
BRI	0.637 (0.609, 0.665)	0.658 (0.626, 0.690)	0.761 (0.690, 0.833)	0.661 (0.640, 0.681)	0.704 (0.675, 0.733)	0.748 (0.694, 0.802)	0.695 (0.627, 0.763)	0.669 (0.652, 0.687)
HC	0.520 (0.492, 0.549)	0.545 (0.510, 0.579)	0.637 (0.556, 0.718)	0.541 (0.519, 0.563)	0.623 (0.594, 0.653)	0.603 (0.544, 0.661)	0.703 (0.637, 0.768)	0.568 (0.550, 0.586)
WHR	0.589 (0.559, 0.618)	0.647 (0.613, 0.680)	0.752 (0.682, 0.823)	0.630 (0.608, 0.651)	0.677 (0.648, 0.706)	0.721 (0.663, 0.778)	0.699 (0.627, 0.770)	0.642 (0.625, 0.660)
Normal weight								
WC	0.588 (0.582, 0.595)	0.688 (0.684, 0.693)	0.716 (0.708, 0.723)	0.681 (0.677, 0.685)	0.696 (0.691, 0.701)	0.742 (0.733, 0.751)	0.707 (0.689, 0.726)	0.691 (0.688, 0.694)
WHtR	0.622 (0.616, 0.628)	0.702 (0.697, 0.706)	0.748 (0.741, 0.755)	0.707 (0.704, 0.711)	0.747 (0.742, 0.752)	0.786 (0.778, 0.794)	0.771 (0.754, 0.788)	0.726 (0.723, 0.729)
ABSI	0.598 (0.592, 0.605)	0.654 (0.649, 0.658)	0.696 (0.688, 0.704)	0.661 (0.657, 0.665)	0.695 (0.690, 0.700)	0.758 (0.749, 0.767)	0.764 (0.747, 0.781)	0.676 (0.673, 0.680)
BRI	0.622 (0.616, 0.628)	0.703 (0.698, 0.707)	0.749 (0.742, 0.756)	0.708 (0.705, 0.711)	0.748 (0.743, 0.752)	0.787 (0.779, 0.796)	0.771 (0.753, 0.788)	0.727 (0.724, 0.730)
HC	0.502 (0.496, 0.509)	0.510 (0.505, 0.515)	0.502 (0.494, 0.511)	0.507 (0.503, 0.511)	0.518 (0.513, 0.524)	0.545 (0.533, 0.556)	0.543 (0.523, 0.564)	0.500 (0.496, 0.503)
WHR	0.601 (0.594, 0.607)	0.706 (0.702, 0.711)	0.740 (0.732, 0.747)	0.700 (0.697, 0.704)	0.730 (0.726, 0.735)	0.789 (0.780, 0.798)	0.748 (0.731, 0.766)	0.716 (0.713, 0.719)
Overweight								
WC	0.533 (0.523, 0.543)	0.585 (0.579, 0.591)	0.607 (0.598, 0.616)	0.606 (0.601, 0.611)	0.635 (0.629, 0.641)	0.683 (0.673, 0.692)	0.677 (0.656, 0.698)	0.638 (0.633, 0.643)
WHtR	0.557 (0.547, 0.567)	0.593 (0.587, 0.599)	0.637 (0.628, 0.646)	0.628 (0.623, 0.633)	0.681 (0.676, 0.687)	0.720 (0.710, 0.729)	0.740 (0.721, 0.758)	0.673 (0.668, 0.678)
ABSI	0.555 (0.545, 0.565)	0.581 (0.575, 0.587)	0.621 (0.612, 0.629)	0.614 (0.608, 0.619)	0.651 (0.645, 0.656)	0.705 (0.696, 0.715)	0.727 (0.707, 0.746)	0.650 (0.645, 0.655)
BRI	0.558 (0.548, 0.568)	0.594 (0.588, 0.600)	0.638 (0.629, 0.646)	0.629 (0.624, 0.634)	0.683 (0.677, 0.688)	0.721 (0.712, 0.730)	0.741 (0.723, 0.759)	0.674 (0.669, 0.679)
HC	0.504 (0.494, 0.514)	0.544 (0.538, 0.550)	0.543 (0.534, 0.553)	0.548 (0.542, 0.553)	0.548 (0.541, 0.554)	0.575 (0.564, 0.586)	0.560 (0.537, 0.583)	0.555 (0.550, 0.560)
WHR	0.536 (0.527, 0.546)	0.617 (0.611, 0.623)	0.637 (0.629, 0.646)	0.640 (0.635, 0.645)	0.669 (0.664, 0.675)	0.730 (0.721, 0.739)	0.717 (0.697, 0.736)	0.677 (0.672, 0.682)
Obese								
WC	0.523 (0.501, 0.545)	0.538 (0.526, 0.550)	0.569 (0.552, 0.585)	0.566 (0.555, 0.577)	0.601 (0.590, 0.612)	0.633 (0.616, 0.649)	0.623 (0.585, 0.661)	0.614 (0.602, 0.625)
WHtR	0.547 (0.525, 0.568)	0.533 (0.521, 0.545)	0.592 (0.576, 0.608)	0.577 (0.566, 0.588)	0.635 (0.624, 0.646)	0.663 (0.647, 0.679)	0.676 (0.641, 0.711)	0.640 (0.629, 0.651)
ABSI	0.546 (0.524, 0.568)	0.530 (0.518, 0.541)	0.578 (0.561, 0.594)	0.568 (0.557, 0.579)	0.619 (0.608, 0.630)	0.636 (0.620, 0.653)	0.670 (0.631, 0.708)	0.627 (0.615, 0.638)
BRI	0.548 (0.526, 0.570)	0.533 (0.521, 0.545)	0.592 (0.576, 0.609)	0.577 (0.566, 0.588)	0.636 (0.625, 0.646)	0.665 (0.648, 0.681)	0.678 (0.643, 0.713)	0.641 (0.630, 0.653)
HC	0.501 (0.479, 0.524)	0.529 (0.517, 0.541)	0.523 (0.506, 0.540)	0.534 (0.523, 0.545)	0.530 (0.518, 0.542)	0.527 (0.509, 0.545)	0.508 (0.467, 0.549)	0.536 (0.524, 0.547)
WHR	0.518 (0.496, 0.541)	0.565 (0.554, 0.577)	0.585 (0.569, 0.602)	0.595 (0.585, 0.606)	0.630 (0.619, 0.641)	0.657 (0.641, 0.674)	0.642 (0.604, 0.681)	0.647 (0.636, 0.658)

* *Area under ROC curve contrast test for different indices was significant, P < 0.05; The shadowed numbers in the table mean that AUROC of those indices were similar to each other (P > 0.05) for identifying the outcome within the specific BMI category, and meanwhile those AUROC were the highest in the six anthropometric indices (P < 0.05). AUROC, area under receiver operator curves; ROC, receiver operator curves; BMI, body mass index; WC, waist circumstance; WHtR, waist height ratio; ABSI, a body shape index; BRI, body round index; HC, hip circumstance; WHR, waist hip ratio*.

**Figure 4:**
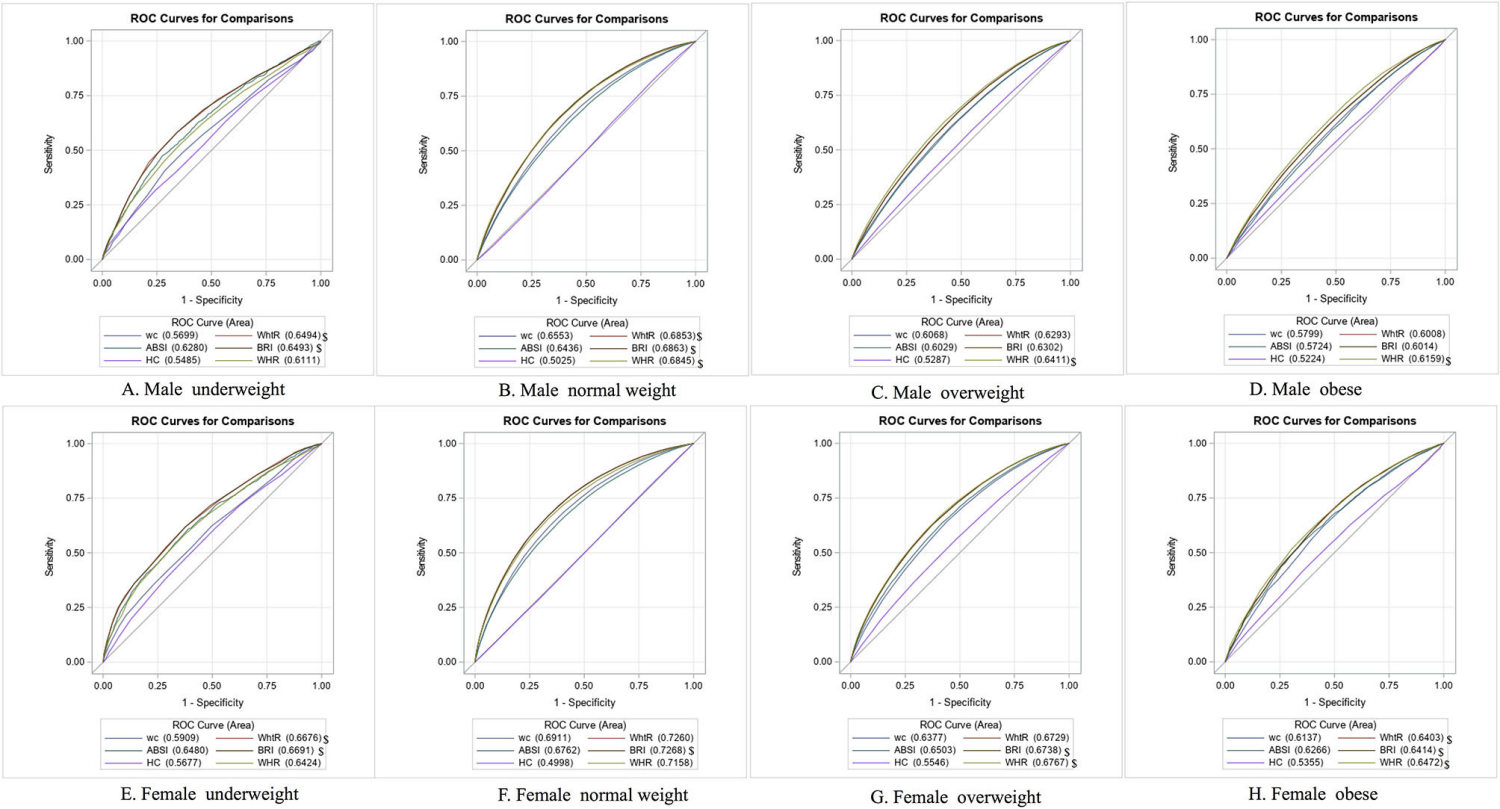
Comparison on area under curves for diagnosing with any one of the CVD risk factors between different waist and hip indices within each BMI category. Comparison on the area under different indices curves was based on Delong test; $ mean that AUROC of those indices were similar to each other (*P* > 0.05) for identifying with any one of CVD risk factors within the specific BMI category, and meanwhile their AUROC were the highest in the six anthropometric indices (*P* < 0.05). AUROC, area under receiver operator curves; ROC, receiver operator curves; BMI, body mass index; WC, waist circumstance; WHtR, waist height ratio; ABSI, a body shape index; BRI, body round index; HC, hip circumstance; WHR, waist hip ratio.

Specifically, for the identification of the presence of at least one CVDRF, WHR had the highest AUROC values in overweight [0.641 (95%CI:0.638, 0.644)] and obese [0.616 (95%CI:0.609, 0.623)] men. On the other hand, BRI had the highest AUROC value in underweight [0.649 (95%CI:0.629, 0.670)] and normal weight [0.686 (95%CI:0.683, 0.690)] men. For the discrimination of any one CVDRF, there was no statistical difference between BRI and WHtR in underweight men, and similarly no statistical difference between BRI and WHtR and WHR in normal men (*p* > 0.05) (Table 2 and Figure 4). Among the women, BRI had the highest discrimination ability in all BMI categories, with AUROC ranging from 0.641 to 0.727. In overweight and obese women, WHR had a nonstatistically significant discriminatory ability with BRI, while WHtR was similar to BRI in the underweight and obese population (*p* > 0.05) (Table 2 and Figure 4).

All six indices had very weak discriminatory ability for hypercholesterolemia and hypertriglyceridemia. For hypercholesterolemia identification in the overweight and obese, the AUROC values were significant but close to 0.5, which might be of little clinical meaning. In the underweight and normal weight population (Table 2 and Supplementary Figure S6), all the indices had improved hypercholesterolemia identification ability, and BRI had the highest stable and statistically significant AUROC values ranging from 0.557 to 0.637 compared to other indices. WHtR had a discriminatory ability not significantly different from that of BRI except for underweight women. For hypertriglyceridemia (Table 2 and Supplementary Figure S7), WHR had the best discriminatory ability in all populations except for normal-weight men, with AUROC values ranging from 0.526 to 0.706. WC had the best discriminatory ability in normal-weight male population (AUROC = 0.638 (95%: 0.635, 0.642)). The ability of WHR to identify mixed hyperlipidaemia was similar to that for hypercholesterolemia, which also had the highest discriminatory power in all populations, except for normal-weight women, with AUROC values ranging from 0.544 to 0.756 (Table 2 and Supplementary Figure S8). Moreover, the BRI had the best discriminatory ability for mixed subtype identification in normal-weight women [AUROC = 0.749 (95%: 0.742, 0.756)]. For dyslipidaemia identification (Table 2 and Supplementary Figure S9), WHR had the best ability in all men and obese/overweight women, with AUROC values ranging from 0.558 to 0.655, and BRI had the best discriminatory ability in underweight and normal-weight female populations (AUROC = 0.661 (95%: 0.640, 0.681) and 0.708 (95%: 0.705, 0.711), respectively).

Compared to other indicators, the BRI had the highest significant discriminatory ability for hypertension (Table 2 and Supplementary Figure S10) and CKD identification (Table 2 and Supplementary Figure S11) in all populations. AUROC values ranged from 0.605 to 0.748 and 0.664 to 0.771, respectively.

For DM identification (Table 2 and Supplementary Figure S12), WHR had the best discriminatory ability in the normal and overweight population, obese men and underweight women with AUROC values ranging from 0.646 to 0.730; on the other hand, BRI had the highest AUROC in underweight men and obese women [AUROC = 0.689 (95%: 0.634, 0.744) and 0.665 (95%: 0.648, 0.681), respectively].

### BRI, WHtR, and WHR Threshold Development

Since the BRI and WHR alternately had the highest discriminatory ability for CVDRFs identification, the optimal BMI-specific BRI and WHR thresholds for each CVDRFs are listed in Supplementary Table S1. Considering that WHtR had a similar discriminatory ability to BRI in most cases and was easier to calculate, the optimal BMI-specific WHtR threshold is also listed in Supplementary Table S1. Generally, the optimal BRI, WHtR, and WHR thresholds were higher in men than in women, except in the underweight population. The optimal threshold for CKD identification was always the highest among those of all the studied CVDRFs. As the cut-off value increased, the sensitivity increased and the specificity decreased. After balancing the sensitivity and specificity, the identification of most CVDRFs, only one cut-off value for each sex, and BMI category was determined as the recommended value and is listed in Table 3. In males, BRI thresholds of 1.8, 3.0, 3.9, and 5.0, WHtR thresholds of 0.41, 0.48, 0.53, and 0.58, and WHR thresholds of 0.81, 0.88, 0.92, and 0.95 were identified as optimal thresholds across the normal weight, overweight, and obese populations, respectively (Table 3). The corresponding BRI cut-off values in women were 1.9, 2.9, 4.0, and 5.2 respectively, and WHtR were 0.41, 0.48, 0.54, and 0.59, whereas the WHR values were 0.77, 0.83, 0.88, and 0.90. To make the use of BRI more feasible, we plotted the BRI chart for high-risk CVDRF identification based on the recommended cut-offs (Supplementary Figures S13, S14).

**Table 3:** Suggested BMI-specific BRI, WHtR, and WHR thresholds and their capacities for identifying participants with CVD associated risk among the Chinese.

**Risk factors**	**Underweight**		**Normal weight**		**Overweight**		**Obese**	
	**Sensitivity**	**Specificity**	**Sensitivity**	**Specificity**	**Sensitivity**	**Specificity**	**Sensitivity**	**Specificity**
Male	BRI ≥ 1.8		BRI ≥ 3.0		BRI ≥ 3.9		BRI ≥ 5.0	
Hypercholesterolemia	58.6	61.3	51.4	56.8	51.8	51.8	51.2	53.6
Hypertriglyceridemia	57.4	61.9	57.8	61.0	51.6	54.1	47.4	54.5
Mixed hyperlipidemia	65.7	60.9	68.0	57.7	57.5	52.8	50.0	54.1
Dyslipidemia	58.4	62.9	58.5	64.0	52.7	57.3	48.1	57.3
Hypertension	60.9	63.0	63.7	60.4	61.7	56.7	55.6	59.2
Diabetic Mellitus	65.5	61.3	71.6	58.2	68.5	53.8	62.8	55.9
Chronic kidney disease	65.8	61.5	70.0	57.1	73.6	52.3	70.1	54.0
With at least one risk	58.4	65.6	58.4	68.6	54.0	64.2	48.9	65.0
	WHtR ≥ 0.41		WHtR ≥ 0.48		WHtR ≥ 0.53		WHtR ≥ 0.58	
Hypercholesterolemia	58.6	61.3	54.5	53.4	52.7	51.0	55.9	49.6
Hypertriglyceridemia	57.4	61.9	61.7	57.8	52.4	53.2	51.5	50.5
Mixed hyperlipidemia	65.7	60.9	71.2	54.4	58.3	51.9	54.2	50.1
Dyslipidemia	58.4	62.8	62.2	60.8	53.5	56.5	52.3	53.5
Hypertension	60.9	62.9	66.9	57.0	62.6	55.9	59.8	55.1
Diabetic Mellitus	65.5	61.3	74.8	54.9	69.2	52.9	67.0	51.9
Chronic kidney disease	65.8	61.5	72.4	53.8	74.2	51.5	73.9	49.9
With at least one risk	58.4	65.5	62.0	65.5	54.8	63.4	53.1	61.1
	WHR ≥0.81		WHR ≥0.88		WHR ≥0.92		WHR ≥0.95	
Hypercholesterolemia	49.6	61.3	50.2	56.3	49.3	52.5	51.3	48.8
Hypertriglyceridemia	56.7	62.2	58.7	60.7	51.9	55.5	52.9	50.7
Mixed hyperlipidemia	74.3	61.2	67.2	57.3	58.2	53.6	56.5	49.7
Dyslipidemia	56.0	62.9	58.9	63.5	52.9	59.2	53.6	54.5
Hypertension	51.3	62.4	62.9	59.8	60.8	57.3	59.9	54.2
Diabetic Mellitus	57.5	61.4	75.4	58.1	71.1	54.8	69.4	51.5
Chronic kidney disease	56.4	61.5	65.5	56.6	69.2	53.0	65.0	49.1
With at least one risk	52.6	64.5	58.7	68.1	53.8	66.0	54.1	62.1
Female	BRI ≥ 1.9		BRI ≥ 2.9		BRI ≥ 4.0		BRI ≥ 5.2	
Hypercholesterolemia	58.6	60.9	50.6	66.2	51.7	56.5	55.4	50.2
Hypertriglyceridemia	61.6	60.8	61.8	67.9	54.1	59.0	53.1	51.2
Mixed hyperlipidemia	71.9	69.9	70.4	66.3	62.7	57.6	61.9	51.4
Dyslipidemia	61.4	61.6	59.6	70.6	55.5	62.9	55.4	55.0
Hypertension	59.03	71.0	68.7	68.3	64.9	61.8	62.8	56.9
Diabetic Mellitus	66.3	70.1	77.6	66.1	74.5	58.0	70.6	52.5
Chronic kidney disease	58.6	69.9	73.4	65.6	76.8	56.5	76.5	50.4
With at least one risk	52.1	71.7	60.2	72.7	56.8	68.3	57.0	63.0
	WHtR ≥ 0.41		WHtR ≥ 0.48		WHtR ≥ 0.54		WHtR ≥ 0.59	
Hypercholesterolemia	58.6	60.9	55.9	49.6	55.9	49.6	57.7	48.0
Hypertriglyceridemia	61.6	60.8	51.5	50.5	51.5	50.5	55.1	48.9
Mixed hyperlipidemia	54.2	50.1	67.2	68.9	58.0	62.1	64.9	49.2
Dyslipidemia	61.4	61.6	59.8	55.1	59.8	55.1	57.7	52.9
Hypertension	59.8	55.1	65.9	70.9	60.1	66.3	65.3	54.8
Diabetic Mellitus	67.0	51.9	75.0	68.7	70.3	62.5	67.0	51.9
Chronic kidney disease	65.5	60.5	73.9	49.9	73.9	49.9	73.9	49.9
With at least one risk	61.9	62.3	53.1	61.1	53.1	61.1	59.3	60.9
	WHR ≥ 0.77		WHR ≥ 0.83		WHR ≥ 0.88		WHR ≥ 0.90	
Hypercholesterolemia	50.9	61.5	47.1	66.7	45.0	59.9	53.0	48.1
Hypertriglyceridemia	60.3	61.7	68.7	62.0	53.4	63.4	58.4	51.0
Mixed hyperlipidemia	73.7	61.3	68.7	67.0	57.8	61.0	63.5	49.5
Dyslipidemia	56.5	62.2	65.3	64.5	52.8	67.0	58.8	54.8
Hypertension	64.7	62.3	65.4	68.7	59.8	65.0	64.5	55.0
Diabetic Mellitus	73.7	61.5	76.9	66.8	72.3	61.6	71.4	50.5
Chronic kidney disease	65.5	61.4	71.1	66.2	71.7	60.0	48.5	71.0
With at least one risk	58.2	62.9	58.4	73.1	53.2	71.7	59.3	62.1

*BRI, body round index; WHtR, waist height ratio; WHR, waist hip ratio*.

By using BRI BMI-specific cut-offs (Table 3) to create high-risk and low-risk exposed status, we found that persons with high BRI exposure in each BMI category showed 19% to 180% significantly higher risk of hypertriglyceridemia, mixed hyperlipidaemia, dyslipidaemia, hypertension, DM, or presence of with at least one CVDRF than the corresponding low-BRI group (Table 4). When using BMI-specific cut-offs for WHtR or WHR (Table 3), a discrimination ability similar to that of BRI was found. Compared to the low WHtR group, those with high WHtR had an 18–175% increased risk of hypertriglyceridemia, mixed hyperlipidemia, dyslipidemia, hypertension, DM, or at least one CVDRF, whereas those with high WHR had a 15–333% increased risk of the above compared to low WHR (Table 4). However, the recommended BRI, WHtR, or WHR BMI-specific cut-offs could not significantly identify the high-risk CKD or hypercholesterolemia population (Table 4). Furthermore, stratification analysis was conducted to test the influence of age on those associations. In the underweight, no significant interaction was found between age and anthropometric indices (*p* > 0.05). However, compared to the 20–39 or 40–59 aged nonunderweight group, significant but slightly lower ORs were observed in the 60 or over-aged group (*p* < 0.05) (Supplementary Table S2).

**Table 4:** Adjusted odds ratios for high BRI, high WHtR, high WHR, and CVD risk factors within each BMI category among Chinese^*^.

**Risk factors**	**Male**				**Female**			
	**Underweight**	**Normal weight**	**Overweight**	**Obese**	**Underweight**	**Normal weight**	**Overweight**	**Obese**
Index = BRI	High-BRI ≥1.8	High-BRI ≥3.0	High-BRI ≥3.9	High-BRI ≥5.0	High-BRI ≥1.9	High-BRI ≥2.9	High-BRI ≥4.0	High-BRI ≥5.2
Hypercholesterolemia	1.49 (1.03, 2.14)	0.99 (0.93, 1.06)	0.91 (0.47, 1.77)	0.93 (0.83, 1.05)	1.29 (1.03, 1.62)	1.00 (0.95, 1.05)	0.87 (0.80, 0.94)	0.88 (0.75, 1.05)
Hypertriglyceridemia	2.42 (1.87, 3.14)	2.43 (2.35, 2.51)	1.43 (1.40, 1.46)	1.21 (1.16, 1.25)	1.53 (1.19, 1.95)	2.45 (2.36, 2.56)	1.50 (1.43, 1.57)	1.19 (1.10, 1.30)
Mixed hyperlipidemia	2.80 (1.35, 5.83)	2.79 (2.61, 2.97)	1.52 (1.47, 1.58)	1.19 (1.13, 1.26)	2.56 (1.39, 4.71)	2.23 (2.07, 2.40)	1.46 (1.35, 1.57)	1.35 (1.18, 1.54)
Dyslipidemia	2.18 (1.77, 2.69)	2.55 (2.48, 2.63)	1.63 (1.59, 1.66)	1.39 (1.33, 1.45)	1.49 (1.27, 1.76)	2.00 (1.94, 2.06)	1.50 (1.44, 1.56)	1.28 (1.18, 1.39)
Hypertension	1.20 (0.94, 1.54)	1.59 (1.53, 1.65)	1.36 (1.32, 1.39)	1.39 (1.34, 1.45)	1.36 (1.07, 1.75)	1.75 (1.67, 1.83)	1.46 (1.39, 1.54)	1.38 (1.26, 1.51)
Diabetic Mellitus	1.82 (1.15, 2.88)	2.15 (2.03, 2.29)	1.64 (1.58, 1.72)	1.67 (1.57, 1.77)	1.67 (1.06, 2.63)	2.50 (2.26, 2.76)	2.21 (2.02, 2.43)	1.96 (1.71, 2.25)
Chronic kidney disease	1.45 (0.96, 2.17)	1.44 (1.30, 1.59)	1.23 (1.12, 1.35)	1.23 (1.04, 1.45)	0.84 (0.48, 1.46)	1.07 (0.90, 1.26)	1.14 (0.94, 1.39)	1.16 (0.81, 1.65)
With at least one risk	1.74 (1.48, 2.05)	2.34 (2.28, 2.41)	1.72 (1.68, 1.77)	1.57 (1.49, 1.66)	1.35 (1.17, 1.56)	2.01 (1.95, 2.07)	1.67 (1.60, 1.74)	1.56 (1.43, 1.71)
Index = WHtR	High-WHtR ≥0.41	High-WHtR ≥0.48	High-WHtR ≥0.53	High-WHtR ≥0.58	High-WHtR ≥0.41	High-WHtR ≥0.48	High-WHtR ≥0.54	High-WHtR ≥0.59
Hypercholesterolemia	1.46 (1.01, 2.11)	0.98 (0.92, 1.05)	0.91 (0.47, 1.77)	0.97 (0.86, 1.09)	1.30 (1.04, 1.62)	1.01 (0.96, 1.06)	0.61 (0.33, 1.12)	0.89 (0.75, 1.06)
Hypertriglyceridemia	2.32 (1.79, 3.02)	2.49 (2.41, 2.57)	1.40 (1.37, 1.43)	1.21 (1.16, 1.26)	1.76 (1.37, 2.26)	2.40 (2.30, 2.50)	1.47 (1.40, 1.54)	1.18 (1.08, 1.29)
Mixed hyperlipidemia	2.72 (1.31, 5.67)	2.75 (2.57, 2.93)	1.48 (1.42, 1.53)	1.20 (1.13, 1.26)	2.58 (1.33, 5.02)	2.15 (2.00, 2.32)	1.44 (1.34, 1.55)	1.42 (1.24, 1.62)
Dyslipidemia	2.10 (1.70, 2.59)	2.59 (2.51, 2.66)	1.59 (1.55, 1.62)	1.40 (1.34, 1.46)	1.58 (1.34, 1.86)	1.99 (1.93, 2.05)	1.47 (1.41, 1.53)	1.30 (1.20, 1.41)
Hypertension	1.38 (1.09, 1.75)	1.59 (1.53, 1.65)	1.37 (1.33, 1.40)	1.41 (1.35, 1.47)	1.19 (0.93, 1.53)	1.75 (1.67, 1.83)	1.44 (1.37, 1.52)	1.44 (1.31, 1.58)
Diabetic Mellitus	1.84 (1.15, 2.93)	2.21 (2.07, 2.35)	1.62 (1.56, 1.70)	1.71 (1.61, 1.82)	1.56 (0.97, 2.52)	2.43 (2.20, 2.67)	2.16 (1.98, 2.36)	1.97 (1.71, 2.26)
Chronic kidney disease	1.46 (0.97, 2.19)	1.45 (1.30, 1.60)	1.24 (1.13, 1.36)	1.33 (1.13, 1.58)	0.79 (0.44, 1.41)	1.13 (0.96, 1.33)	1.15 (0.95, 1.39)	1.24 (0.87, 1.77)
With at least one risk	1.70 (1.44, 2.00)	2.35 (2.29, 2.42)	1.69 (1.65, 1.73)	1.33 (1.13, 1.58)	1.41 (1.21, 1.63)	2.02 (1.96, 2.08)	1.64 (1.57, 1.71)	1.59 (1.46, 1.74)
Index = WHR	High-WHR ≥0.81	High-WHR ≥0.88	High-WHR ≥0.92	High-WHR ≥0.95	High-WHR ≥0.77	High-WHR ≥0.83	High-WHR ≥0.88	High-WHR ≥0.90
Hypercholesterolemia	1.21 (0.85, 1.73)	0.96 (0.90, 1.02)	0.86 (0.67, 1.10)	1.22 (0.84, 1.78)	1.07 (0.86, 1.33)	1.04 (0.59, 1.84)	1.04 (0.71, 1.51)	1.00 (0.99, 1.00)
Hypertriglyceridemia	2.15 (1.67, 2.76)	2.44 (2.36, 2.52)	1.50 (1.47, 1.54)	1.27 (1.22, 1.32)	1.87 (1.47, 2.39)	2.58 (2.48, 2.69)	1.80 (1.72, 1.88)	1.48 (1.36, 1.62)
Mixed hyperlipidemia	4.33 (2.00, 9.37)	2.49 (2.34, 2.66)	1.56 (1.50, 1.61)	1.28 (1.21, 1.35)	2.41 (1.31, 4.45)	2.29 (2.13, 2.46)	1.45 (1.35, 1.56)	1.41 (1.24, 1.60)
Dyslipidemia	1.95 (1.59, 2.39)	2.48 (2.41, 2.55)	1.72 (1.68, 1.76)	1.51 (1.44, 1.57)	1.46 (1.24, 1.71)	2.02 (1.95, 2.08)	1.69 (1.62, 1.76)	1.53 (1.41, 1.66)
Hypertension	1.21 (0.96, 1.53)	1.56 (1.50, 1.62)	1.38 (1.35, 1.42)	1.40 (1.34, 1.46)	1.43 (1.12, 1.83)	1.67 (1.59, 1.75)	1.50 (1.43, 1.58)	1.58 (1.44, 1.73)
Diabetic Mellitus	1.69 (1.09, 2.64)	2.79 (2.61, 2.97)	2.04 (1.96, 2.13)	1.97 (1.85, 2.10)	2.24 (1.39, 3.60)	2.83 (2.57, 3.13)	2.54 (2.33, 2.77)	2.02 (1.76, 2.31)
Chronic kidney disease	1.35 (0.91, 2.00)	1.28 (1.16, 1.42)	1.25 (1.15, 1.37)	1.09 (0.94, 1.28)	1.15 (0.64, 2.05)	1.22 (1.03, 1.43)	1.38 (1.15, 1.66)	1.19 (0.86, 1.65)
With at least one risk	1.57 (1.33, 1.85)	2.29 (2.23, 2.35)	1.81 (1.76, 1.85)	1.70 (1.62, 1.79)	1.43 (1.24, 1.65)	1.98 (1.93, 2.04)	1.80 (1.73, 1.88)	1.80 (1.65, 1.97)

^*^Adjustment variable including age, smoking status and drinking status. The shadowed numbers in the table mean that those ORs were statistically significant with P < 0.05. BRI, body round index; WHtR, waist height ratio; WHR, waist hip ratio; CKD, chronic kidney disease.

## Discussion

In this cross-sectional study, we compared six noninvasive, low-cost, and easily calculated anthropometric measures, including WC, HC, WHtR, WHR, ABSI, and BRI for their ability to predict CVD risk factors in different BMI categories. In general, our results showed that the new anthropometric index ABSI was not suitable for identifying CVD risk, while BRI and WHR were superior to other anthropometric measures for determining the presence of CVDRFs.

Body mass index has been used as a representative index in studies on obesity and related diseases in the past decade. However, BMI is not considered to be related to the detrimental influence of intraabdominal fat on mortality and morbidity, especially in Asian individuals, who may exhibit a “normal” BMI but have a disproportionately large intraabdominal fat region (34); thus, adiposity indices have been suggested as alternative obesity indices that can modulate the limitations of BMI. Recently, a systematic review (10) indicated that independent of overall adiposity, all indices of central fatness, including WC, WHR, WHtR, and ABSI, were positively and significantly associated with a higher all-cause mortality risk. The summary hazard ratios were as follows: WC (10 cm increase): 1.11 (1.08–1.13); WHR (0.12 unit increase): 1.20 (1.15–1.25); WHtR (0.1 unit increase): 1.24 (1.12–1.36); and ABSI (0.005 unit increase): 1.15 (1.10–1.20). In contrast, a 10-cm increment in HC was associated with a 10% lower risk of all-cause mortality. The results suggest that measures of central adiposity could be used with BMI as a supplementary approach to determine the risk of premature death.

However, researchers have failed to identify the best indicators to predict CVD risk and mortality until now. Many studies have suggested that the strongest predictor among anthropometric indices differs according to CVDRFs, age, sex, ethnicity, and country. For example, WHR was the strongest predictor in Australian adults (35), whereas WC was the best predictor in Canadian adults (36). However, WHtR was the best indicator in several ethnic groups and countries (37, 38). For diabetes, in Chang's study, WC had a similar ability as other anthropometric measures to predict the presence of DM in Northeast China (39). Meanwhile, many studies have indicated that WHtR or WHR is the index most strongly associated with diabetes (40, 41). ABSI and BRI have recently attracted intense attention when linked with cardiovascular disease development and other adverse events. Initial studies reported that ABSI had a stronger association with premature mortality than BMI or WC (42). However, subsequent research revealed conflicting results regarding the predictive ability of ABSI for chronic diseases and mortality (43–45). The BRI has been proven to improve body fat prediction (28) and has been confirmed as an alternative index for assessing insulin resistance, diabetes, and hyperuricemia (46–48). Moreover, the BRI performed similarly to or better than BMI and WC at predicting MetS and MetS components in Peruvian adults (49). It was the most superior predictor and an independent determinant for diabetes among the Chinese hypertensive population (50). However, Chang's results demonstrated that the BRI did not show superior power compared to WC or the WHtR for the prediction of DM (39). A meta-analysis indicated that WC and WHtR offer the best performance when screening for MetS and hypertension, but nonsignificant differences were found with BRI (17, 18). Maessen MF et al. found that the BRI could identify CVD and CVDRFs, but was not superior to BMI or WC (51).

To the best of our knowledge, we first calculated the best indicators of CVDRFs within each BMI category in the Chinese population. In accordance with previous results (43–45), the ABSI showed the weakest capacity for identifying CVDRFs. Generally, the BRI possesses a stable ability to predict CVDRFs in all but overweight and obese men, and this result was well determined in other studies (46–50). The advantage of BRI is commonly believed to improve the predictive power of body fat, and visceral adipose and visceral adipose tissue were well known (28). However, WHR is the best indicator of CVDRFs among overweight and obese men in accordance with previous studies (41, 52). Indeed, the American Heart Association Measurement of the WHR provides no advantage over WC alone and is not recommended as part of the routine obesity evaluation. First, our population is mainly from central-southern China, and differences in ethnicity may lead to inconsistent results. Second, the definition of overweight and obesity is different between China and America. As a natural defect of cross-sectional studies, further cohort research is needed. In summary, two anthropometric measurements, BRI and WHR, achieved better cardiovascular disease risk stratification in the current study. Considering the complexity of the BRI calculation, we plotted the BRI chart to improve the feasibility (Supplementary Figures S13, S14). Physicians could conveniently determine the BRI value and persons with higher CVD risks only from height and weight data. Thirdly, in most cases, WHtR had a similar discriminatory ability to BRI and was easier to calculate. WHtR and WHR were the first choices for the health check-up population to predict CVDRFs.

Furthermore, the WHR and BRI thresholds determined in this study have been rounded to simplify their use in clinical settings. Previous studies have reported WC thresholds within BMI categories. For example, in Ardern's study, WC cut-offs of 90, 100, 110, and 125 cm for underweight, normal, overweight and obese men and 80, 90, 105, and 115 cm for women in US and Canadian populations, respectively, could be used to identify chronic disease risk (53). In the current study, BRI and WHR BMI-specific cut-offs by sex group were established. Although the validity and utility of these cut-offs need further confirmation, the results indicated that using the recommended BRI or WHR BMI-specific cut-offs could significantly distinguish those at high risk of hypertriglyceridemia, mixed hyperlipidaemia, dyslipidaemia, hypertension, DM, or the presence of at least one CVDRF, but that a low area of the ROC curve implies a loss of sensitivity and specificity to identify individuals at high risk of CKD or hypercholesterolemia. This result was consistent with Lee's study (54), which showed that anthropometric indices could identify risk factors, except for hypercholesterolemia. We believe these thresholds can be used as a simple clinical tool for the screening of future CVD risk based on WHR or BRI measurements within specific BMI categories in a health check-up population.

We acknowledge that this study has some limitations. First, this was a cross-sectional study, and relevant findings in this study should be verified in a well-designed cohort study. Second, the health check-up population in Hunan may not represent the majority of the Chinese people. Third, insufficient physical activity and unhealthy food consumption were the major risk factors for deaths and DALYs in the Chinese population (55). However, we failed to adjust that information in the current study. Fourth, some researchers (56, 57) suggested that the optimal anthropometric indices cut-off, including BMI, might differ between the elderly and the young or middle-aged. However, there is still no consistency on age-specific BMI cut-offs among adults. According to our main purpose, we therefore, chose not to calculate the sex-age-specific cut-offs for selected anthropometric indices. We used the stratification analysis to discuss the influence of age instead, and a slightly decreased separation ability of a higher CVDRF subgroup was observed among 60 or over-aged nonunderweight group. Fifth, the WC measurement method is based on the Chinese guideline in the current study (26), but it remains to be determined whether this measurement method is optimal for different age groups (58). Last but not least, there is controversy about using the Youden index as an indicator for setting optimal thresholds on medical tests, which indicates that the ratio of misclassification costs was by default and equal to one minus the prevalence of the interests (59). A higher prevalence of CVDRFs was observed in greater BMI categories. Therefore, in the present study, an optimal selection of thresholds in the overweight and obese groups would be based on lower-cost ratios with higher specificity and predictive value of positive classifications than in the underweight and normal-weight groups. This study also has strengths. The findings and statistical results in the present study are robust due to the large study sample. To the best of our knowledge, this is the first study to develop and crossvalidate WHR, WHtR, and BRI thresholds within BMI categories. Depending on sex, the use of BMI category-specific WHR, WHtR, and BRI thresholds improved the identification of individuals at a high risk of CVD in a Chinese population.

## Conclusion

Our present study found that the BRI was superior to WC, HC, and WHR for predicting the presence of CVDRFs in the Chinese female population. However, predicting CVDRFs in men, the BRI best discriminates in underweight or normal-weight men, whereas the WHR had the best ability in overweight and obese men. The ABSI showed the weakest predictive power. Considering that WHtR had a similar discriminatory ability to BRI and was easier to calculate in most cases, WHtR could be used as an alternative obesity measure for predicting CVDRFs among women and overweight/obese mn. BRI, WHtR, and WHR BMI-specific cut-offs by sex were established, but needed further study.

## Data Availability Statement

The raw data supporting the conclusions of this article will be made available by the authors, without undue reservation.

## Ethics Statement

The studies involving human participants were reviewed and approved by the Ethics Committee of the Third Xiangya Hospital (2020-S498). The patients/participants provided their written informed consent to participate in this study.

## Author Contributions

YL, LY, QL, and XH designed the study. YH, YW, PY, JW, CL, and ZC included patients for the study. YL and XH analyzed the data. YL, LY, XH, and YH wrote the manuscript. LY and QL revised the manuscript. All authors reviewed and edited the manuscript. All authors read and approved the final manuscript.

## Funding

This work was supported by funding from the National Natural Science Foundation of China (81973324 to YL, 81872685 to XH, and 82003537 to XH), Hunan Young Talent grant (2020RC3063 to YL), Natural Science Foundation of Hunan Province (2020JJ5858 to YL, 2020JJ4439 to XH) and the Wisdom Accumulation and Talent Cultivation Project of the Third XiangYa hospital of Central South University (YX202002 to YL). The funders had no role in the study design, data collection and analysis, decision to publish, or preparation of the manuscript.

## Conflict of Interest

The authors declare that the research was conducted in the absence of any commercial or financial relationships that could be construed as a potential conflict of interest.

## Publisher's Note

All claims expressed in this article are solely those of the authors and do not necessarily represent those of their affiliated organizations, or those of the publisher, the editors and the reviewers. Any product that may be evaluated in this article, or claim that may be made by its manufacturer, is not guaranteed or endorsed by the publisher.

## References

[1] Must A Spadano J Coakley EH Field AE Colditz G Dietz WH. The disease burden associated with overweight and obesity. JAMA. (1999) 282:1523–9. 10.1001/jama.282.16.1523 10546691

[2] Lauby-Secretan B Scoccianti C Loomis D Grosse Y Bianchini F Straif K. Body Fatness and Cancer–Viewpoint of the IARC Working Group. N Engl J Med. (2016) 375:794–8. 10.1056/NEJMsr1606602 27557308 PMC6754861

[3] Ayton A Ibrahim A. Obesity is a public health emergency. BMJ. (2019) 366:l5463. 10.1136/bmj.l5463 31519652

[4] Yin Y Li Y Shao L Yuan S Liu B Lin S. Effect of Body Mass Index on the Prognosis of Liver Cirrhosis. Front Nutr. (2021) 8:700132. 10.3389/fnut.2021.700132 34490322 PMC8417598

[5] Anstey KJ Cherbuin N Budge M Young J. Body mass index in midlife and late-life as a risk factor for dementia: a meta-analysis of prospective studies. Obes Rev. (2011) 12:e426–437. 10.1111/j.1467-789X.2010.00825.x 21348917

[6] Singh GM Danaei G Farzadfar F Stevens GA Wood ward M Wormser D. The age-specific quantitative effects of metabolic risk factors on cardiovascular diseases and diabetes: a pooled analysis. PLoS ONE. (2013) 8:e65174. 10.1371/journal.pone.0065174 23935815 PMC3728292

[7] Czernichow S Kengne AP Stamatakis E Hamer M Batty GD. Body mass index, waist circumference and waist-hip ratio: which is the better discriminator of cardiovascular disease mor- tality risk?: evidence from an individual-participant meta-analysis of 82 864 participants from nine cohort studies. Obes Rev. (2011) 12:680–7. 10.1111/j.1467-789X.2011.00879.x 21521449 PMC4170776

[8] Tzoulaki I Elliott P Kontis V Ezzati M. Worldwide Exposures to Cardiovascular Risk Factors and Associated Health Effects: Current Knowledge and Data Gaps. Circulation. (2016) 133:2314–33. 10.1161/CIRCULATIONAHA.115.008718 27267538

[9] Lee DY Lee MY Sung KC. Prediction of Mortality with A Body Shape Index in Young Asians: Comparison with Body Mass Index and Waist Circumference. Obesity (Silver Spring). (2018) 26:1096–103. 10.1002/oby.22193 29719128

[10] Jayedi A Soltani S Zargar MS Khan TA Shab-Bidar S. Central fatness and risk of all cause mortality: systematic review and dose-response meta-analysis of 72 prospective cohort studies. BMJ. (2020) 370:m3324. 10.1136/bmj.m3324 32967840 PMC7509947

[11] Heymsfield SB Scherzer R Pietrobelli A Lewis CE Grunfeld C. Int J Obes (Lond). (2009) 12:1363–73. 10.1038/ijo.2009.184 19773739 PMC3156622

[12] Kang SM Yoon JW Ahn HY Kim SY Lee KH Shin H. Android fat depot is more closely associated with metabolic syndrome than abdominal visceral fat in elderly people. PLoS ONE. (2011) 6:e27694. 10.1371/journal.pone.0027694 22096613 PMC3214067

[13] Zhang FL Ren JX Zhang P Jin H Qu Y Yu Y. Strong Association of Waist Circumference (WC), Body Mass Index (BMI), Waist-to-Height Ratio (WHtR), and Waist-to-Hip Ratio (WHR) with Diabetes: A Population-Based Cross-Sectional Study in Jilin Province, China. J Diabetes Res. (2021) 2021:8812431. 10.1155/2021/8812431 34056007 PMC8147550

[14] Ashwell M Gunn P Gibson S. Waist-to-height ratio is a better screening tool than waist circumference and BMI for adult cardiometabolic risk factors: systematic review and meta-analysis. Obes Rev. (2012) 13:275–86. 10.1111/j.1467-789X.2011.00952.x 22106927

[15] Pasdar Y Moradi S Moludi J Saiedi S Moradinazar M Hamzeh B. Waist-to-height ratio is a better discriminator of cardiovascular disease than other anthropometric indicators in Kurdish adults. Sci Rep. (2020) 10:16228. 10.1038/s41598-020-73224-8 33004896 PMC7530727

[16] Kuk JL Ardern CI. Influence of age on the association between various measures of obesity and all-cause mortality. J Am Geriatr Soc. (2009) 57:2077–84. 10.1111/j.1532-5415.2009.02486.x 19754497

[17] Rico-Martín S Calderón-García JF Sánchez-Rey P Franco-Antonio C Martínez Alvarez M Sánchez Muñoz-Torrero JF. Effectiveness of body roundness index in predicting metabolic syndrome: a systematic review and meta-analysis. Obes Rev. (2020) 21:e13023. 10.1111/obr.13023 32267621

[18] Calderón-García JF Roncero-Martín R Rico-Martín S De Nicolás-Jiménez JM López-Espuela F Santano-Mogena E. Effectiveness of Body Roundness Index (BRI) and a Body Shape Index (ABSI) in Predicting Hypertension: A Systematic Review and Meta-Analysis of Observational Studies. Int J Environ Res Public Health. (2021) 18:11607. 10.3390/ijerph182111607 34770120 PMC8582804

[19] Janssen I Katzmarzyk PT Ross R. Body mass index, waist circumference, and health risk: evidence in support of current National Institutes of Health guidelines. Arch Intern Med. (2002) 162:2074–9. 10.1001/archinte.162.18.2074 12374515

[20] Ardern CI Katzmarzyk PT Janssen I Ross R. Discrimination of health risk by combined body mass index and waist circumference. Obes Res. (2003) 11:135–42. 10.1038/oby.2003.22 12529496

[21] De Vincentis A Tavaglione F Spagnuolo R Pujia R Tuccinardi D Mascianà G. Metabolic and genetic determinants for progression to severe liver disease in subjects with obesity from the UK Biobank. Int J Obes (Lond). (2021). 10.1038/s41366-021-01015-w. [Epub ahead of print]. 34750514 PMC8573310

[22] Ross R Neeland IJ Yamashita S Shai I Seidell J Magni P. Waist circumference as a vital sign in clinical practice: a Consensus Statement from the IAS and ICCR Working Group on Visceral Obesity. Nat Rev Endocrinol. (2020) 16:177–89. 10.1038/s41574-019-0310-7 32020062 PMC7027970

[23] Sadeghi M Talaei M Gharipour M Oveisgharan S Nezafati P Dianatkhah M. Anthropometric indices predicting incident hypertension in an Iranian population: The Isfahan cohort study. Anatol J Cardiol. (2019) 22:33–43. 10.14744/AnatolJCardiol.2019.10594 31264654 PMC6683211

[24] Christakoudi S Tsilidis KK Muller DC Freisling H Weiderpass E Overvad K. A Body Shape Index (ABSI) achieves better mortality risk stratification than alternative indices of abdominal obesity: results from a large European cohort. Sci Rep. (2020) 10:14541. 10.1038/s41598-020-71302-5 32883969 PMC7471961

[25] Korhonen PE Jaatinen PT Aarnio PT Kantola IM Saaresranta T. Waist circumference home measurement–a device to find out patients in cardiovascular risk. Eur J Public Health. (2009) 19:95–9. 10.1093/eurpub/ckn090 18927187

[26] Chen C Lu FC. Department of Disease Control Ministry of Health, PR China. The guidelines for prevention and control of overweight and obesity in Chinese adults. Biomed Environ Sci. (2004) 17 Suppl:1–36. 15807475

[27] Krakauer NY Krakauer JC. A new body shape index predicts mortality hazard independently of body mass index. PLoS ONE. (2012) 7:e39504. 10.1371/journal.pone.0039504 22815707 PMC3399847

[28] Thomas DM Bredlau C Bosy-Westphal A Mueller M Shen W Gallagher D. Relationships between body roundness with body fat and visceral adipose tissue emerging from a new geometrical model. Obesity (Silver Spring). (2013) 21:2264–71. 10.1002/oby.20408 23519954 PMC3692604

[29] Joint committee issued Chinese guideline for the management of dyslipidemia in adults. [2016 Chinese guideline for the management of dyslipidemia in adults]. Zhonghua Xin Xue Guan Bing Za Zhi. (2016) 44:833–53. [in Chinese]. 10.3760/cma.j.issn.0253-3758.2016.10.005 27903370

[30] Joint Committee for Guideline Revision. 2018 Chinese Guidelines for Prevention and Treatment of Hypertension-A report of the Revision Committee of Chinese Guidelines for Prevention and Treatment of Hypertension. J Geriatr Cardiol. (2019) 16:182-241. 10.11909/j.issn.1671-5411.2019.03.014 31080465 PMC6500570

[31] Chinese Diabetes Society. Guidelines for the Prevention and Treatment of Type 2 Diabetes in China (2020 Edition). (Part 1). Chin J Pract Intern Med. (2021) 41:668–95. [in Chinese]. 10.19538/j.nk2021080106

[32] Matsushita K Mahmoodi BK Woodward M Emberson JR Jafar TH Jee SH. Comparison of risk prediction using the CKD-EPI equation and the MDRD study equation for estimated glomerular filtration rate. JAMA. (2012) 307:1941–51. 10.1001/jama.2012.3954 22570462 PMC3837430

[33] Kidney Disease: Improving Global Outcomes (KDIGO) CKD Work Group. KDIGO 2012 clinical practice guideline for the evaluation and management of chronic kidney disease. Kidney Int Suppl. (2013) 3:1–150. 10.1038/kisup.2012.73

[34] Deurenberg P Deurenberg-Yap M Guricci S. Asians are different from Caucasians and from each other in their body mass index/body fat per cent relationship. Obes Rev. (2002) 3:141–6. 10.1046/j.1467-789X.2002.00065.x 12164465

[35] Dalton M Cameron AJ Zimmet PZ Shaw JE Jolley D Dunstan DW. Waist circumference, waist-hip ratio and body mass index and their correlation with cardiovascular disease risk factors in Australian adults. J Intern Med. (2003) 254:555–63. 10.1111/j.1365-2796.2003.01229.x 14641796

[36] Dobbelsteyn CJ Joffres MR MacLean DR Flowerdew G. A comparative evaluation of waist circumference, waist-to-hip ratio and body mass index as indicators of cardiovascular risk factors. The Canadian Heart Health Surveys. Int J Obes Relat Metab Disord. (2001) 25:652–61. 10.1038/sj.ijo.0801582 11360147

[37] Nguyen Minh Q Nguyen Vo MH. Anthropometric Indexes for Predicting High Blood Pressure in Vietnamese Adults: A Cross-Sectional Study. Integr Blood Press Control. (2020) 13:181–6. 10.2147/IBPC.S281996 33293857 PMC7718968

[38] Lee CM Huxley RR Wildman RP Woodward M. Indices of abdominal obesity are better discriminators of cardiovascular risk factors than BMI: a meta-analysis. J Clin Epidemiol. (2008) 61:646–53. 10.1016/j.jclinepi.2007.08.012 18359190

[39] Chang Y Guo X Chen Y Guo L Li Z Yu S. A body shape index and body roundness index: two new body indices to identify diabetes mellitus among rural populations in northeast China. BMC Public Health. (2015) 15:794. 10.1186/s12889-015-2150-2 26286520 PMC4544789

[40] Corrêa MM Thumé E De Oliveira ER Tomasi E. Performance of the waist-to-height ratio in identifying obesity and predicting non-communicable diseases in the elderly population: a systematic literature review. Arch Gerontol Geriatr. (2016) 65:174–82. 10.1016/j.archger.2016.03.021 27061665

[41] Kapoor N Lotfaliany M Sathish T Thankappan KR Thomas N Furler J. Obesity indicators that best predict type 2 diabetes in an Indian population: insights from the Kerala Diabetes Prevention Program. J Nutr Sci. (2020) 9:e15. 10.1017/jns.2020.8 32328239 PMC7163399

[42] Dhana K Kavousi M Ikram MA Tiemeier HW Hofman A Franco OH. Body shape index in comparison with other anthropometric measures in prediction of total and cause-specific mortality. J Epidemiol Community Health. (2016) 70:90–6. 10.1136/jech-2014-205257 26160362

[43] Song X Jousilahti P Stehouwer CD Söderberg S Onat A Laatikainen T. Cardiovascular and all-cause mortality in relation to various anthropometric measures of obesity in Europeans. Nutr Metab Cardiovasc Dis. (2015) 25:295–304. 10.1016/j.numecd.2014.09.004 25315666

[44] He S Zheng Y Wang H Chen X. Assessing the relationship between a body shape index and mortality in a group of middle-aged men. Clin Nutr. (2017) 36:1355–9. 10.1016/j.clnu.2016.09.003 27663543

[45] Fujita M Sato Y Nagashima K Takahashi S Hata A. Predictive power of a body shape index for development of diabetes, hypertension, and dyslipidemia in Japanese adults: a retrospective cohort study. PLoS ONE. (2015) 10:e0128972. 10.1371/journal.pone.0128972 26030122 PMC4451769

[46] Zhao Q Zhang K Li Y Zhen Q Shi J Yu Y. Capacity of a body shape index and body roundness index to identify diabetes mellitus in Han Chinese people in Northeast China: a cross-sectional study. Diabet Med. (2018) 35:1580–7. 10.1111/dme.13787 30059165

[47] Feng J He S Chen X. Body Adiposity Index and Body Roundness Index in Identifying Insulin Resistance Among Adults Without Diabetes. Am J Med Sci. (2019) 357:116–23. 10.1016/j.amjms.2018.11.006 30665492

[48] Zhang N Chang Y Guo X Chen Y Ye N Sun Y. Body Shape Index and Body Roundness Index: Two new body indices for detecting association between obesity and hyperuricemia in rural area of China. Eur J Intern Med. (2016) 29:32–6. 10.1016/j.ejim.2016.01.019 26895753

[49] Stefanescu A Revilla L Lopez T Sanchez SE Williams MA Gelaye B. Using A Body Shape Index (ABSI) and Body Roundness Index (BRI) to predict risk of metabolic syndrome in Peruvian adults. J Int Med Res. (2020) 48:300060519848854. 10.1177/0300060519848854 31144540 PMC7140225

[50] Liu Y Liu X Guan H Zhang S Zhu Q Fu X. Body Roundness Index Is a Superior Obesity Index in Predicting Diabetes Risk Among Hypertensive Patients: A Prospective Cohort Study in China. Front Cardiovasc Med. (2021) 8:736073. 10.3389/fcvm.2021.736073 34869638 PMC8638826

[51] Maessen MF Eijsvogels TM Verheggen RJ Hopman MT Verbeek AL de Vegt F. Entering a new era of body indices: the feasibility of a body shape index and body roundness index to identify cardiovascular health status. PLoS ONE. (2014) 9:e107212. 10.1371/journal.pone.0107212 25229394 PMC4167703

[52] Lee BJ Kim JY. Identification of Type 2 Diabetes Risk Factors Using Phenotypes Consisting of Anthropometry and Triglycerides based on Machine Learning. IEEE J Biomed Health Inform. (2016) 20:39–46. 10.1109/JBHI.2015.2396520 25675467

[53] Ardern CI Janssen I Ross R Katzmarzyk PT. Development of health-related waist circumference thresholds within BMI categories. Obes Res. (2004) 12:1094–103. 10.1038/oby.2004.137 15292473

[54] Lee BJ Yim MH. Comparison of anthropometric and body composition indices in the identification of metabolic risk factors. Sci Rep. (2021) 11:9931. 10.1038/s41598-021-89422-x 33976292 PMC8113511

[55] Zhou M Wang H Zeng X Yin P Zhu J Chen W. Mortality, morbidity, and risk factors in China and its provinces, 1990-2017: a systematic analysis for the Global Burden of Disease Study 2017. Lancet. (2019) 394:1145–58. 10.1016/S0140-6736(19)30427-1 31248666 PMC6891889

[56] Matsuo T Sairenchi T Iso H Irie F Tanaka K Fukasawa N. Age- and gender-specific BMI in terms of the lowest mortality in Japanese general population. Obesity (Silver Spring). (2008) 16:2348–55. 10.1038/oby.2008.342 18719651

[57] de HollanderELVan ZutphenMBogersRPBemelmansWJDe GrootLC. The impact of body mass index in old age on cause-specific mortality. J Nutr Health Aging. (2012) 16:100–6. 10.1007/s12603-011-0077-622238008

[58] Guerra RS Amaral TF Marques EA Mota J Restivo MT. Anatomical location for waist circumference measurement in older adults: a preliminary study. Nutr Hosp. (2012) 27:1554-61. 10.3305/nh.2012.27.5.5922 23478705

[59] Smits N. A note on Youden's J and its cost ratio. BMC Med Res Methodol. (2010) 10:89. 10.1186/1471-2288-10-8 20920288 PMC2959030

