# Supplementary material for: Body Roundness Index and Waist–Hip Ratio Result in Better Cardiovascular Disease Risk Stratification: Results From a Large Chinese Cross-Sectional Study
Source: Front Nutr. 2022 Mar 10;9:801582. doi: 10.3389/fnut.2022.801582 (PMC8960742; doi:10.3389/fnut.2022.801582)
Supplement: Supplementary file 1 [file Data_Sheet_1.pdf]

## Supplemental Material

**Table S1**. Optimal BMI-specific BRI, WHtR and WHR thresholds for identifying participants with CVD associated risk factor among Chinese

**Table S2**. Odds ratios for high BRI, high WHtR and high WHR and having at least one CVD risk factors by age stratification

**Figure S1.** Distribution of anthropometric indices by gender and BMI category among Hunan Chinese

**Figure S2.** Association between CVD risk factors and anthropometric indices among underweight Chinese

**Figure S3.** Association between CVD risk factors and anthropometric indices among normal weight Chinese *

**Figure S4.** Association between CVD risk factors and anthropometric indices among overweight Chinese *

**Figure S5.** Association between CVD risk factors and anthropometric indices among obese Chinese *

**Figure S6.** Comparison on area under curves for diagnosing hypercholesterolemia between different waist and hip indices within each BMI category

**Figure S7.** Comparison on area under curves for diagnosing hypertriglyceridemia between different waist and hip indices within each BMI category

**Figure S8.** Comparison on area under curves for diagnosing mixed hyperlipidemia between different waist and hip indices within each BMI category

**Figure S9.** Comparison on area under curves for diagnosing dyslipidemia between different waist and hip indices within each BMI category

**Figure S10.** Comparison on area under curves for diagnosing hypertension between different waist and hip indices within each BMI category

**Figure S11.** Comparison on area under curves for diagnosing chronic kidney disease between different waist and hip indices within each BMI category

**Figure S12.** Comparison on area under curves for diagnosing diabetic mellitus between different waist and hip indices within each BMI category

**Figure S13.** Body roundness index chart for high risk CVDRFs identification among male

**Figure S14.** Body roundness index chart for high risk CVDRFs identification among female

Table S1. Optimal BMI-specific BRI, WHtR and WHR thresholds for identifying participants with CVD associated risk factor among Chinese

| Risk factor | Underweight | | | | Normal weight | | | | Overweight | | | | Obese | | | |
|---|---|---|---|---|---|---|---|---|---|---|---|---|---|---|---|---|
| | Cut-off | Sensitivity | Specificity | Youden | Cut-off | Sensitivity | Specificity | Youden | Cut-off | Sensitivity | Specificity | Youden | Cut-off | Sensitivity | Specificity | Youden |
| Index=BRI | | | | | | | | | | | | | | | | |
| Female | | | | | | | | | | | | | | | | |
| Hypercholesterolemia | 1.9 | 58.6 | 60.9 | 0.195 | 2.8 | 56.8 | 60.7 | 0.174 | 3.9 | 57.6 | 51.1 | 0.088 | 5.3 | 51.6 | 54.0 | 0.056 |
| Hypertriglyceridemia | 1.9 | 61.6 | 60.8 | 0.224 | 2.8 | 67.8 | 62.4 | 0.302 | 3.9 | 59.8 | 53.6 | 0.134 | 5.2 | 53.1 | 51.2 | 0.043 |
| Mixed hyperlipidemia | 2.0 | 71.9 | 69.9 | 0.418 | 2.9 | 70.4 | 66.3 | 0.367 | 4.0 | 62.7 | 57.6 | 0.203 | 5.3 | 57.8 | 55.1 | 0.129 |
| Dyslipidemia | 1.9 | 61.4 | 61.6 | 0.231 | 2.8 | 65.6 | 65.1 | 0.307 | 3.9 | 61.1 | 57.6 | 0.187 | 5.2 | 55.4 | 55.0 | 0.104 |
| Hypertension | 2.0 | 59.0 | 71.0 | 0.300 | 2.9 | 68.7 | 68.3 | 0.370 | 4.0 | 64.9 | 61.8 | 0.267 | 5.2 | 62.8 | 56.9 | 0.197 |
| Diabetic Mellitus | 1.9 | 66.3 | 70.1 | 0.364 | 3.0 | 72.4 | 71.1 | 0.435 | 4.2 | 64.6 | 68.0 | 0.327 | 5.4 | 63.8 | 60.3 | 0.241 |
| Chronic kidney disease | 1.9 | 58.6 | 69.9 | 0.285 | 3.1 | 66.4 | 75.2 | 0.416 | 4.2 | 68.3 | 66.5 | 0.348 | 5.6 | 60.0 | 65.8 | 0.258 |
| With at least one risk | 1.8 | 61.9 | 62.4 | 0.243 | 2.8 | 66.1 | 67.2 | 0.334 | 3.9 | 62.4 | 63.0 | 0.254 | 5.0 | 65.3 | 55.4 | 0.207 |
| Male | | | | | | | | | | | | | | | | |
| Hypercholesterolemia | 1.8 | 58.6 | 61.3 | 0.200 | 3.0 | 51.4 | 56.8 | 0.082 | 3.9 | 51.8 | 51.8 | 0.036 | 4.9 | 57.8 | 48.0 | 0.058 |
| Hypertriglyceridemia | 1.8 | 57.4 | 61.9 | 0.193 | 2.9 | 65.2 | 54.7 | 0.199 | 3.9 | 51.6 | 54.1 | 0.057 | 4.9 | 53.0 | 48.8 | 0.018 |
| Mixed hyperlipidemia | 1.8 | 65.7 | 60.9 | 0.266 | 3.0 | 68.0 | 57.7 | 0.257 | 3.9 | 57.5 | 52.8 | 0.103 | 4.9 | 56.0 | 48.5 | 0.045 |
| Dyslipidemia | 1.8 | 58.4 | 62.9 | 0.213 | 2.9 | 65.7 | 57.7 | 0.234 | 3.8 | 59.9 | 50.7 | 0.106 | 4.9 | 53.8 | 51.8 | 0.056 |
| Hypertension | 1.8 | 60.9 | 63.0 | 0.238 | 3.0 | 63.7 | 60.4 | 0.241 | 3.9 | 61.7 | 56.7 | 0.184 | 5.0 | 55.6 | 59.2 | 0.148 |
| Diabetic Mellitus | 1.8 | 65.5 | 61.3 | 0.268 | 3.1 | 64.6 | 64.7 | 0.293 | 4.0 | 61.9 | 60.5 | 0.224 | 5.1 | 57.8 | 60.9 | 0.187 |
| Chronic kidney disease | 1.8 | 65.8 | 61.5 | 0.273 | 3.2 | 58.2 | 69.8 | 0.280 | 4.1 | 62.2 | 65.3 | 0.276 | 5.2 | 60.3 | 64.3 | 0.246 |
| With at least one risk | 1.8 | 58.4 | 65.6 | 0.239 | 2.9 | 65.3 | 62.4 | 0.276 | 3.8 | 61.0 | 57.5 | 0.186 | 4.8 | 60.2 | 54.2 | 0.144 |
| Index=WHtR | | | | | | | | | | | | | | | | |
| Female | | | | | | | | | | | | | | | | |

| | | | | | | | | | | | | | | | | |
|---|---|---|---|---|---|---|---|---|---|---|---|---|---|---|---|---|
| Hypercholesterolemia | 0.41 | 58.6 | 60.9 | 0.195 | 0.47 | 58.3 | 59.4 | 0.177 | 0.53 | 58.1 | 50.6 | 0.086 | 0.59 | 57.7 | 48.0 | 0.057 |
| Hypertriglyceridemia | 0.41 | 61.6 | 60.8 | 0.224 | 0.47 | 69.1 | 61.2 | 0.303 | 0.53 | 60.3 | 53.0 | 0.133 | 0.59 | 55.1 | 48.9 | 0.040 |
| Mixed hyperlipidemia | 0.42 | 66.7 | 74.2 | 0.409 | 0.48 | 67.2 | 68.9 | 0.361 | 0.54 | 58.0 | 62.1 | 0.201 | 0.59 | 64.9 | 49.2 | 0.141 |
| Dyslipidemia | 0.41 | 61.4 | 61.6 | 0.230 | 0.47 | 67.0 | 63.9 | 0.309 | 0.53 | 61.6 | 57.0 | 0.187 | 0.59 | 57.7 | 52.9 | 0.105 |
| Hypertension | 0.42 | 67.1 | 61.7 | 0.288 | 0.48 | 65.9 | 70.9 | 0.368 | 0.54 | 60.1 | 66.3 | 0.264 | 0.59 | 65.3 | 54.8 | 0.200 |
| Diabetic Mellitus | 0.42 | 63.2 | 74.4 | 0.376 | 0.48 | 75.0 | 68.7 | 0.436 | 0.54 | 70.3 | 62.5 | 0.328 | 0.60 | 65.0 | 59.0 | 0.240 |
| Chronic kidney disease | 0.41 | 65.5 | 60.5 | 0.260 | 0.49 | 64.9 | 76.5 | 0.414 | 0.55 | 63.6 | 70.8 | 0.344 | 0.61 | 60.5 | 65.4 | 0.259 |
| With at least one risk | 0.41 | 61.9 | 62.3 | 0.243 | 0.47 | 67.4 | 66.0 | 0.334 | 0.53 | 63.0 | 62.4 | 0.253 | 0.59 | 59.3 | 60.9 | 0.202 |
| Male | | | | | | | | | | | | | | | | |
| Hypercholesterolemia | 0.41 | 58.6 | 61.3 | 0.199 | 0.48 | 54.5 | 53.4 | 0.079 | 0.53 | 52.7 | 51.0 | 0.037 | 0.58 | 55.9 | 49.6 | 0.055 |
| Hypertriglyceridemia | 0.41 | 57.4 | 61.9 | 0.193 | 0.48 | 61.7 | 57.8 | 0.195 | 0.53 | 52.4 | 53.2 | 0.056 | 0.58 | 51.5 | 50.5 | 0.020 |
| Mixed hyperlipidemia | 0.41 | 65.7 | 60.9 | 0.266 | 0.48 | 71.2 | 54.4 | 0.256 | 0.53 | 58.3 | 51.9 | 0.102 | 0.58 | 54.2 | 50.1 | 0.043 |
| Dyslipidemia | 0.41 | 58.4 | 62.8 | 0.213 | 0.48 | 62.2 | 60.8 | 0.230 | 0.53 | 53.5 | 56.5 | 0.100 | 0.58 | 52.3 | 53.5 | 0.057 |
| Hypertension | 0.41 | 60.9 | 62.9 | 0.238 | 0.48 | 66.9 | 57.0 | 0.239 | 0.53 | 62.6 | 55.9 | 0.184 | 0.58 | 59.8 | 55.1 | 0.149 |
| Diabetic Mellitus | 0.41 | 65.5 | 61.3 | 0.268 | 0.49 | 62.4 | 66.6 | 0.290 | 0.54 | 55.9 | 66.1 | 0.220 | 0.59 | 55.2 | 63.3 | 0.185 |
| Chronic kidney disease | 0.41 | 65.8 | 61.5 | 0.273 | 0.49 | 61.9 | 65.5 | 0.274 | 0.54 | 62.7 | 64.7 | 0.274 | 0.59 | 62.5 | 61.3 | 0.239 |
| With at least one risk | 0.41 | 58.4 | 65.5 | 0.239 | 0.48 | 62.0 | 65.5 | 0.275 | 0.53 | 54.8 | 63.4 | 0.182 | 0.58 | 53.1 | 61.1 | 0.141 |
| Index=WHR | | | | | | | | | | | | | | | | |
| Female | | | | | | | | | | | | | | | | |
| Hypercholesterolemia | 0.76 | 62.6 | 51.5 | 0.141 | 0.81 | 61.3 | 53.8 | 0.151 | 0.87 | 52.2 | 53.2 | 0.054 | 0.90 | 53.0 | 48.1 | 0.011 |
| Hypertriglyceridemia | 0.77 | 60.3 | 61.7 | 0.219 | 0.83 | 68.7 | 62.0 | 0.307 | 0.87 | 60.3 | 56.8 | 0.171 | 0.91 | 51.1 | 59.1 | 0.102 |
| Mixed hyperlipidemia | 0.78 | 70.2 | 69.0 | 0.392 | 0.84 | 68.7 | 67.0 | 0.357 | 0.87 | 65.4 | 54.4 | 0.199 | 0.90 | 63.5 | 49.5 | 0.13 |
| Dyslipidemia | 0.77 | 56.5 | 62.2 | 0.187 | 0.83 | 65.3 | 64.5 | 0.298 | 0.87 | 59.9 | 60.6 | 0.205 | 0.90 | 58.8 | 54.8 | 0.136 |
| Hypertension | 0.77 | 64.7 | 62.3 | 0.270 | 0.83 | 65.4 | 68.7 | 0.341 | 0.88 | 59.8 | 65.0 | 0.248 | 0.90 | 64.5 | 55.0 | 0.195 |
| Diabetic Mellitus | 0.77 | 73.7 | 61.5 | 0.352 | 0.84 | 71.3 | 72.5 | 0.438 | 0.89 | 65.0 | 68.4 | 0.335 | 0.92 | 58.6 | 64.5 | 0.231 |
| Chronic kidney disease | 0.78 | 58.6 | 69.1 | 0.277 | 0.83 | 71.1 | 66.2 | 0.374 | 0.89 | 65.5 | 66.8 | 0.323 | 0.91 | 64 | 56.3 | 0.203 |

| | | | | | | | | | | | | | | | | |
|---|---|---|---|---|---|---|---|---|---|---|---|---|---|---|---|---|
| With at least one risk | 0.77 | 58.2 | 62.9 | 0.211 | 0.82 | 65.5 | 66.5 | 0.320 | 0.87 | 60.2 | 65.4 | 0.257 | 0.90 | 59.3 | 62.1 | 0.214 |
| Male | | | | | | | | | | | | | | | | |
| Hypercholesterolemia | 0.81 | 49.6 | 61.3 | 0.109 | 0.87 | 57.8 | 49.0 | 0.069 | 0.92 | 49.3 | 52.5 | 0.018 | 0.95 | 51.3 | 48.8 | 0.001 |
| Hypertriglyceridemia | 0.81 | 56.7 | 62.2 | 0.188 | 0.88 | 58.7 | 60.7 | 0.194 | 0.92 | 51.9 | 55.5 | 0.074 | 0.95 | 52.9 | 50.7 | 0.036 |
| Mixed hyperlipidemia | 0.82 | 74.3 | 68.7 | 0.430 | 0.88 | 67.2 | 57.3 | 0.244 | 0.92 | 58.2 | 53.6 | 0.118 | 0.95 | 56.5 | 49.7 | 0.062 |
| Dyslipidemia | 0.81 | 56.0 | 62.9 | 0.189 | 0.88 | 58.9 | 63.5 | 0.224 | 0.91 | 62 | 50.7 | 0.127 | 0.95 | 53.6 | 54.5 | 0.081 |
| Hypertension | 0.80 | 61.7 | 52.3 | 0.139 | 0.88 | 62.9 | 59.8 | 0.226 | 0.92 | 60.8 | 57.3 | 0.182 | 0.95 | 59.9 | 54.2 | 0.141 |
| Diabetic Mellitus | 0.81 | 57.5 | 61.4 | 0.189 | 0.89 | 68.4 | 65.9 | 0.343 | 0.93 | 63.4 | 63.0 | 0.265 | 0.96 | 60.1 | 60.4 | 0.205 |
| Chronic kidney disease | 0.80 | 68.4 | 51.4 | 0.198 | 0.89 | 58.2 | 64.4 | 0.226 | 0.93 | 61.9 | 61.2 | 0.231 | 0.96 | 58.5 | 58.1 | 0.166 |
| With at least one risk | 0.81 | 52.6 | 64.5 | 0.171 | 0.87 | 66.1 | 60.9 | 0.271 | 0.91 | 62.9 | 57.7 | 0.205 | 0.95 | 54.1 | 62.1 | 0.162 |

Abbreviation: BRI, body round index; WHtR, waist height ratio; WHR, waist hip ratio.

Table S2. Odds ratios for high BRI, high WHtR and high WHR and with at least one CVD risk factors by age stratification

| Age strata | Male | | | | Female | | | |
|---|---|---|---|---|---|---|---|---|
| | Underweight | Normal weight | Overweight | Obese | Underweight | Normal weight | Overweight | Obese |
| Index=BRI | High-BRI ≥1.8 | High-BRI ≥3.0 | High-BRI ≥3.9 | High-BRI ≥5.0 | High-BRI ≥1.9 | High-BRI ≥2.9 | High-BRI ≥4.0 | High-BRI ≥5.2 |
| <40 years | 1.90(1.47,2.46) | 2.86(2.74,2.98) | 2.10(2.02,2.18) | 1.79(1.66,1.93) | 1.41(1.15,1.74) | 2.63(2.50,2.77) | 2.05(1.90,2.21) | 1.91(1.65,2.20) |
| 40-59 years | 1.79(1.52,2.11) | 2.35(2.29,2.42) | 1.70(1.66,1.75) | 1.57(1.48,1.66) | 1.47(1.26,1.70) | 2.24(2.17,2.30) | 1.85(1.77,1.92) | 1.66(1.52,1.82) |
| ≥ 60 years | 1.68(1.32,2.14) | 1.93(1.83,2.04) | 1.38(1.31,1.46) | 1.38(1.22,1.55) | 1.52(1.17,1.98) | 1.90(1.79,2.02) | 1.66(1.54,1.80) | 1.45(1.21,1.73) |
| $P$ interact* | 0.526 | <0.001 | <0.001 | 0.002 | 0.695 | <0.001 | 0.002 | 0.042 |
| Index=WHtR | High-WHtR ≥0.41 | High-WHtR ≥0.48 | High-WHtR ≥0.53 | High-WHtR ≥0.58 | High-WHtR ≥0.41 | High-WHtR ≥0.48 | High-WHtR ≥0.54 | High-WHtR ≥0.59 |
| <40 years | 1.89(1.46,2.45) | 2.86(2.75,2.98) | 2.09(2.01,2.17) | 1.74(1.61,1.87) | 1.49(1.22,1.81) | 2.67(2.54,2.81) | 2.07(1.92,2.24) | 1.90(1.65,2.19) |
| 40-59 years | 1.79(1.52,2.10) | 2.37(2.31,2.44) | 1.70(1.65,1.74) | 1.58(1.49,1.67) | 1.49(1.28,1.74) | 2.24(2.18,2.31) | 1.83(1.75,1.90) | 1.67(1.53,1.83) |
| ≥ 60 years | 1.68(1.32,2.15) | 1.97(1.87,2.07) | 1.38(1.31,1.46) | 1.44(1.27,1.62) | 1.50(1.13,1.98) | 1.88(1.77,1.99) | 1.61(1.49,1.74) | 1.47(1.23,1.76) |
| $P$ interact* | 0.541 | <0.001 | <0.001 | 0.023 | 0.977 | <0.001 | <0.001 | 0.061 |
| Index=WHR | High-WHR ≥0.81 | High-WHR ≥0.88 | High-WHR ≥0.92 | High-WHR ≥0.95 | High-WHR ≥0.77 | High-WHR ≥0.83 | High-WHR ≥0.88 | High-WHR ≥0.90 |
| <40 years | 1.91(1.48,2.46) | 2.89(2.77,3.01) | 2.29(2.20,2.38) | 2.07(1.92,2.23) | 1.40(1.15,1.71) | 2.54(2.41,2.67) | 2.43(2.25,2.62) | 2.19(1.90,2.52) |
| 40-59 years | 1.63(1.39,1.91) | 2.34(2.27,2.40) | 1.86(1.81,1.91) | 1.67(1.58,1.77) | 1.57(1.35,1.82) | 2.24(2.18,2.31) | 1.99(1.92,2.08) | 1.87(1.71,2.04) |
| ≥ 60 years | 1.39(1.10,1.76) | 1.89(1.79,1.99) | 1.51(1.43,1.59) | 1.35(1.20,1.53) | 1.76(1.35,2.30) | 1.98(1.87,2.10) | 1.64(1.51,1.77) | 1.59(1.34,1.90) |
| $P$ interact* | 0.097 | <0.001 | <0.001 | <0.001 | 0.223 | <0.001 | <0.001 | 0.019 |

*$P$ interact: p value for statistical interaction on a multiplicative scale, derived from a likelihood ratio test comparing a model including the cross-classification anthropometric indices by-BMI-by-age group with a model including separate variables for anthropometric indices-by-BMI and age;
Abbreviation: BRI, body round index; WHtR, waist height ratio; WHR, waist hip ratio;

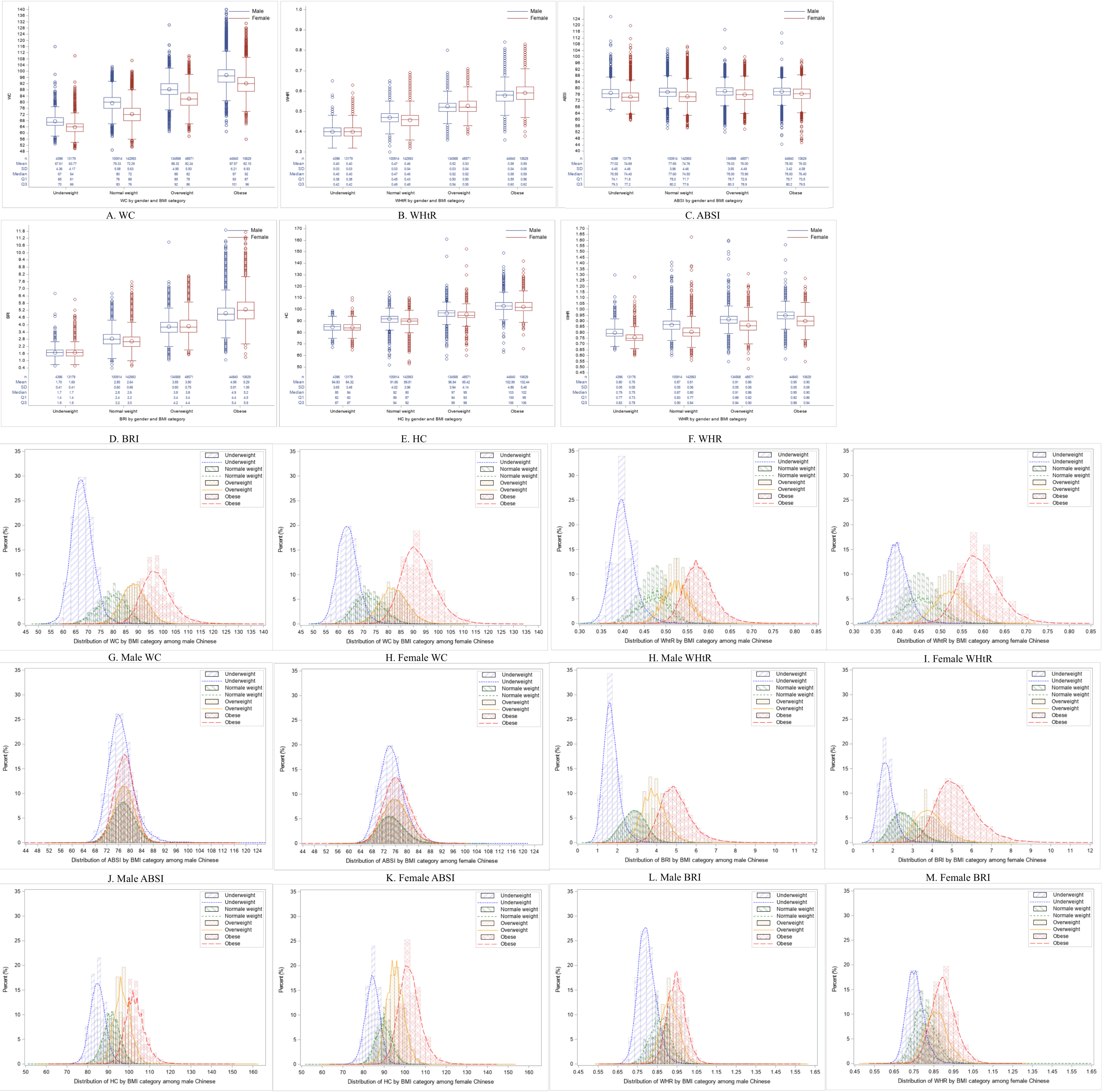

A. WC B. WHtR C. ABSI

D. BRI E. HC F. WHR

G. Male WC H. Female WC H. Male WHtR I. Female WHtR

J. Male ABSI K. Female ABSI L. Male BRI M. Female BRI

I. Male HC H. Female HC K. Male WHR J. Female WHR

Figure S1. Distribution of anthropometric indices by gender and BMI category among Hunan Chinese

A-F: Boxplot showing the distribution of anthropometric indices by gender and BMI category ; G-J: kernel density estimates (KDE) using Gaussian kernels to display anthropometric indices distribution overlaps between different BMI categories ; Abbreviation: n, number of persons; SD, standard deviation; Q1, first quartile, the 25th percentile; Q3, third quartile, the 75th percentile; BMI, body mass index; WC, waist circumstance; WHtR, waist height ratio; ABSI, a body shape index; BRI, body round index; HC, hip circumstance; WHR, waist hip ratio.

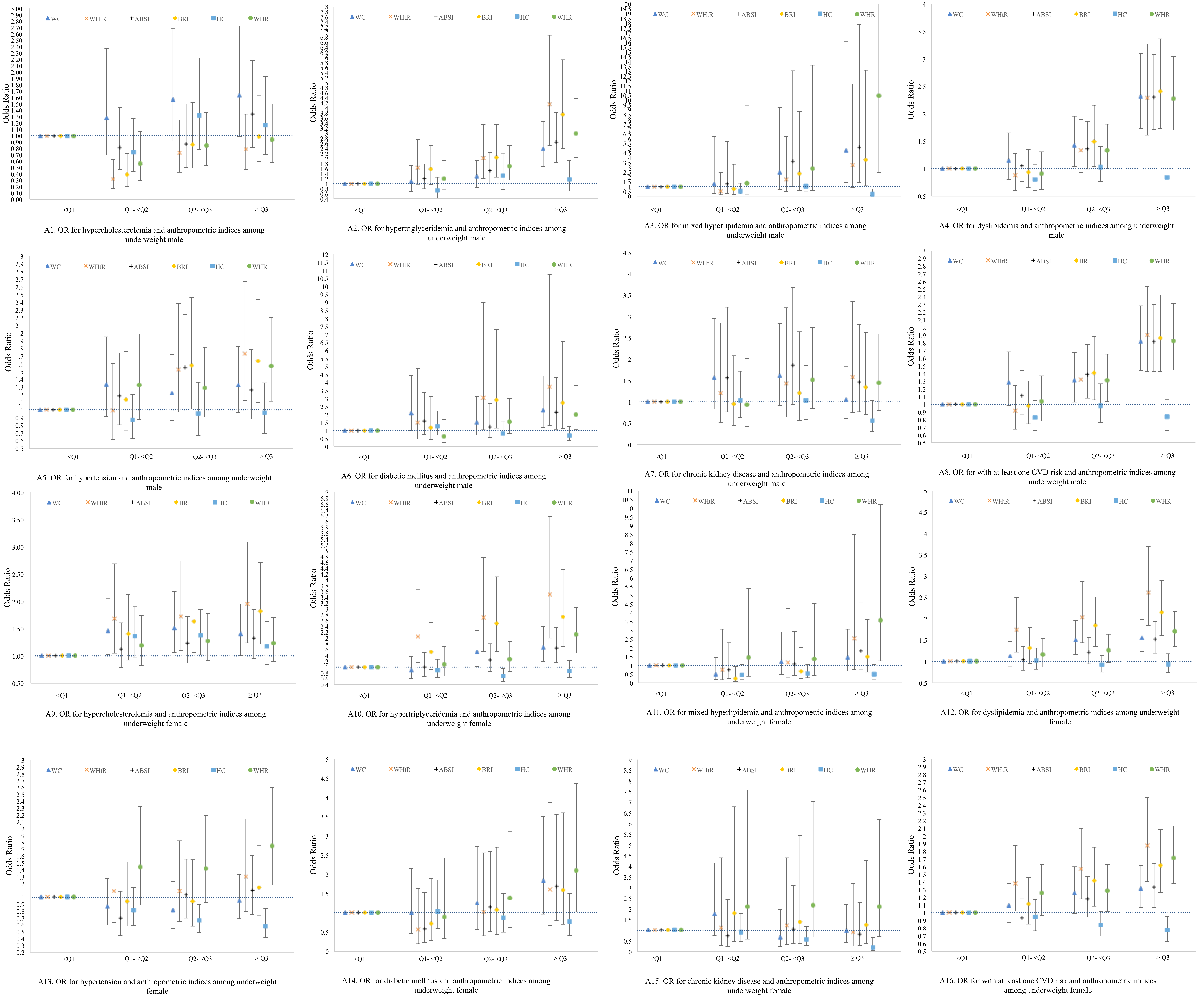


A1. OR for hypercholesterolemia and anthropometric indices among underweight male

A2. OR for hypertriglyceridemia and anthropometric indices among underweight male

A3. OR for mixed hyperlipidemia and anthropometric indices among underweight male

A4. OR for dyslipidemia and anthropometric indices among underweight male

A5. OR for hypertension and anthropometric indices among underweight male

A6. OR for diabetic mellitus and anthropometric indices among underweight male

A7. OR for chronic kidney disease and anthropometric indices among underweight male

A8. OR for with at least one CVD risk and anthropometric indices among underweight male

A9. OR for hypercholesterolemia and anthropometric indices among underweight female

A10. OR for hypertriglyceridemia and anthropometric indices among underweight female

A11. OR for mixed hyperlipidemia and anthropometric indices among underweight female

A12. OR for dyslipidemia and anthropometric indices among underweight female

A13. OR for hypertension and anthropometric indices among underweight female

A14. OR for diabetic mellitus and anthropometric indices among underweight female

A15. OR for chronic kidney disease and anthropometric indices among underweight female

A16. OR for with at least one CVD risk and anthropometric indices among underweight female

Figure S2. Association between CVD risk factors and anthropometric indices among underweight Chinese

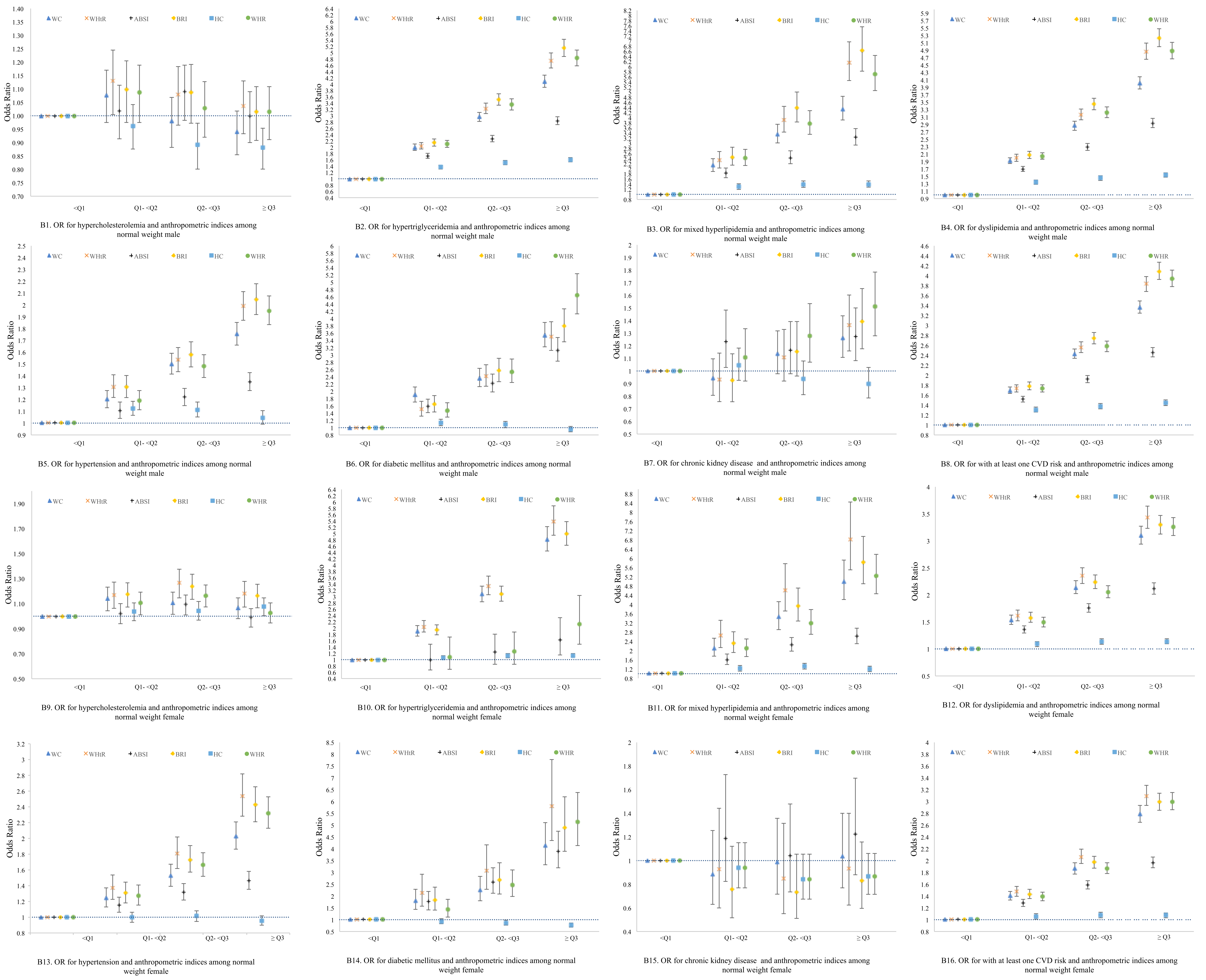


B1. OR for hypercholesterolemia and anthropometric indices among normal weight male

B2. OR for hypertriglyceridemia and anthropometric indices among normal weight male

B3. OR for mixed hyperlipidemia and anthropometric indices among normal weight male

B4. OR for dyslipidemia and anthropometric indices among normal weight male

B5. OR for hypertension and anthropometric indices among normal weight male

B6. OR for diabetic mellitus and anthropometric indices among normal weight male

B7. OR for chronic kidney disease and anthropometric indices among normal weight male

B8. OR for with at least one CVD risk and anthropometric indices among normal weight male

B9. OR for hypercholesterolemia and anthropometric indices among normal weight female

B10. OR for hypertriglyceridemia and anthropometric indices among normal weight female

B11. OR for mixed hyperlipidemia and anthropometric indices among normal weight female

B12. OR for dyslipidemia and anthropometric indices among normal weight female

B13. OR for hypertension and anthropometric indices among normal weight female

B14. OR for diabetic mellitus and anthropometric indices among normal weight female

B15. OR for chronic kidney disease and anthropometric indices among normal weight female

B16. OR for with at least one CVD risk and anthropometric indices among normal weight female

Figure S3. Association between CVD risk factors and anthropometric indices among normal weight Chinese *

* association was adjusted for age, smoking, and drinking status and index at <Q1 level was treated as non-exposed;
Abbreviation: Q1, first quartile, the 25th percentile; Q2: second quartile, median; Q3, third quartile, the 75th percentile; WC, waist circumstance; WHtR, waist height ratio; ABSI, a body shape index; BRI, body round index; HC, hip circumstance; WHR, waist hip ratio.

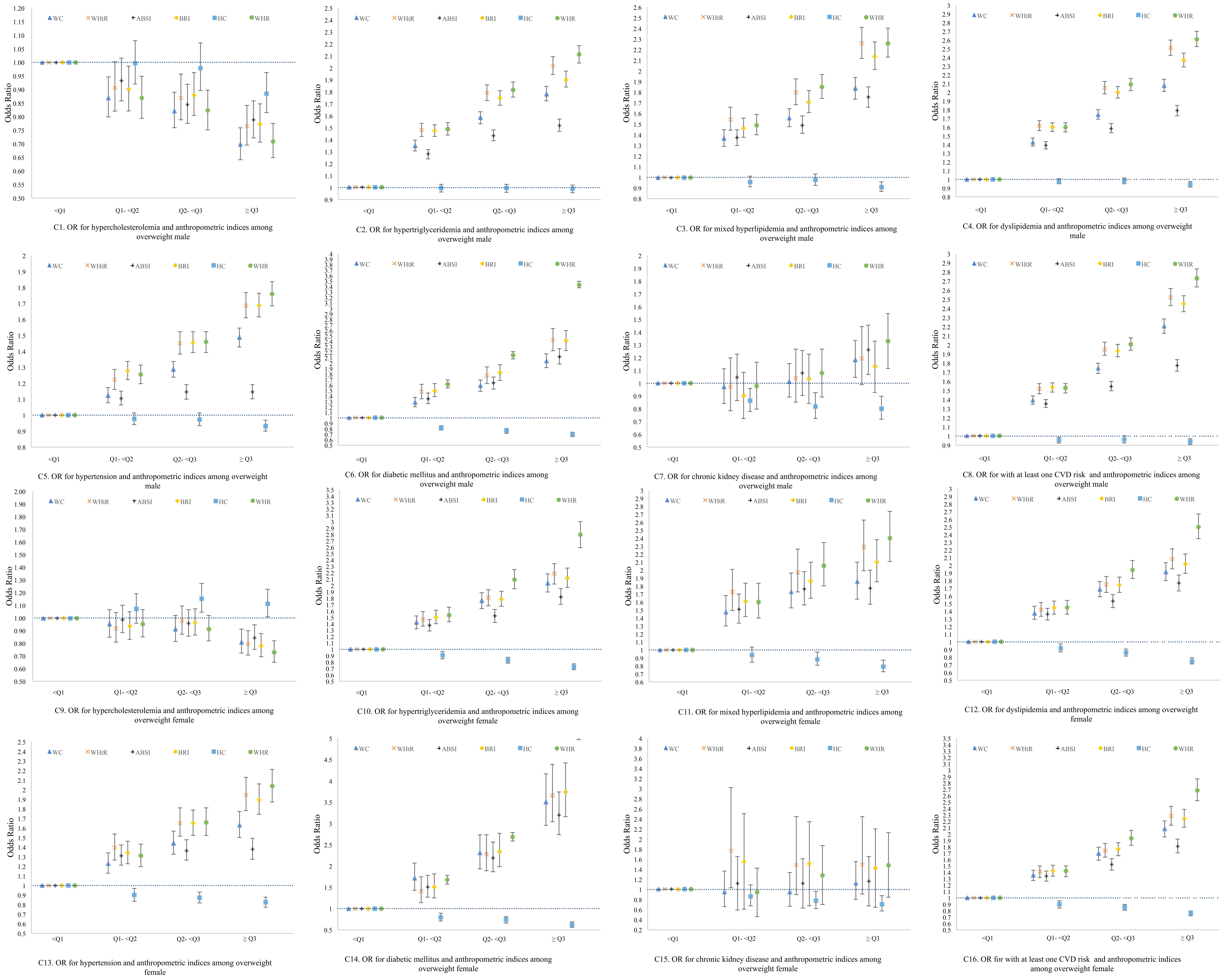


C1. OR for hypercholesterolemia and anthropometric indices among overweight male

C2. OR for hypertriglyceridemia and anthropometric indices among overweight male

C3. OR for mixed hyperlipidemia and anthropometric indices among overweight male

C4. OR for dyslipidemia and anthropometric indices among overweight male

C5. OR for hypertension and anthropometric indices among overweight male

C6. OR for diabetic mellitus and anthropometric indices among overweight male

C7. OR for chronic kidney disease and anthropometric indices among overweight male

C8. OR for with at least one CVD risk and anthropometric indices among overweight male

C9. OR for hypercholesterolemia and anthropometric indices among overweight female

C10. OR for hypertriglyceridemia and anthropometric indices among overweight female

C11. OR for mixed hyperlipidemia and anthropometric indices among overweight female

C12. OR for dyslipidemia and anthropometric indices among overweight female

C13. OR for hypertension and anthropometric indices among overweight female

C14. OR for diabetic mellitus and anthropometric indices among overweight female

C15. OR for chronic kidney disease and anthropometric indices among overweight female

C16. OR for with at least one CVD risk and anthropometric indices among overweight female

Figure S4. Association between CVD risk factors and anthropometric indices among overweight Chinese *

* association was adjusted for age, smoking, and drinking status and index at <Q1 level was treated as non-exposed;

Abbreviation: Q1, first quartile, the 25th percentile; Q2: second quartile, median; Q3, third quartile, the 75th percentile; WC, waist circumstance; WHtR, waist height ratio; ABSI, a body shape index; BRI, body round index; HC, hip circumstance; WHR, waist hip ratio.

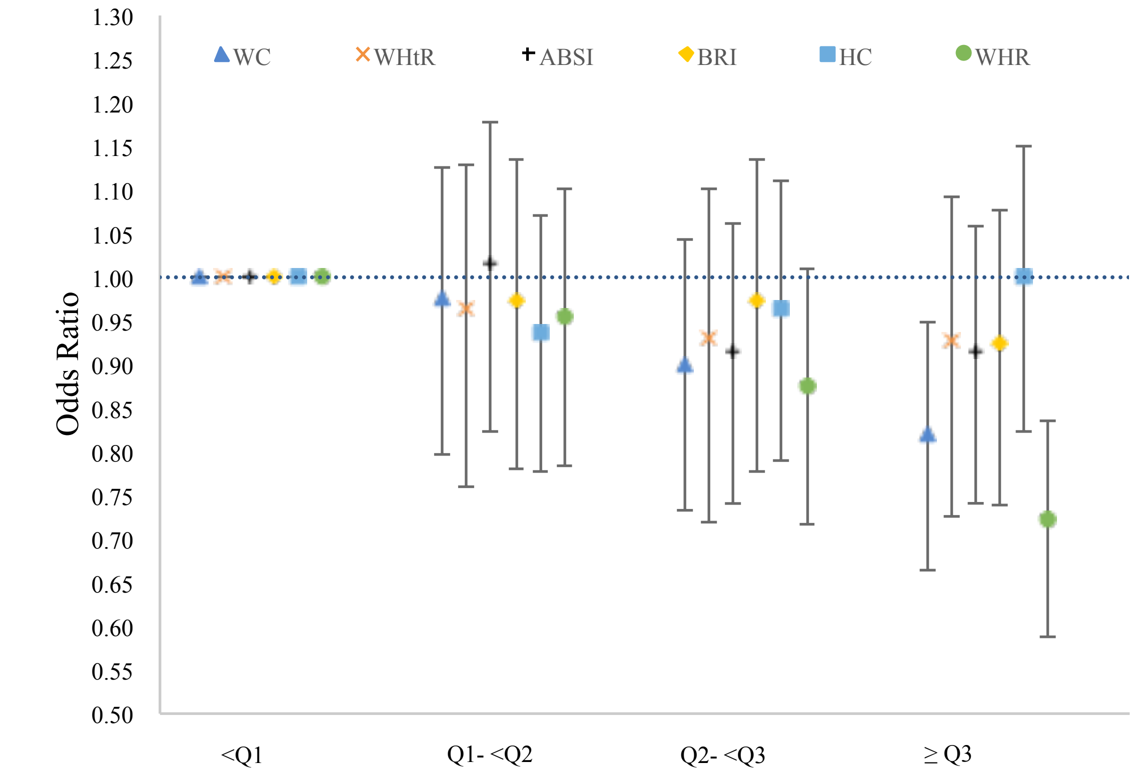

D1. OR for hypercholesterolemia and anthropometric indices among obese male

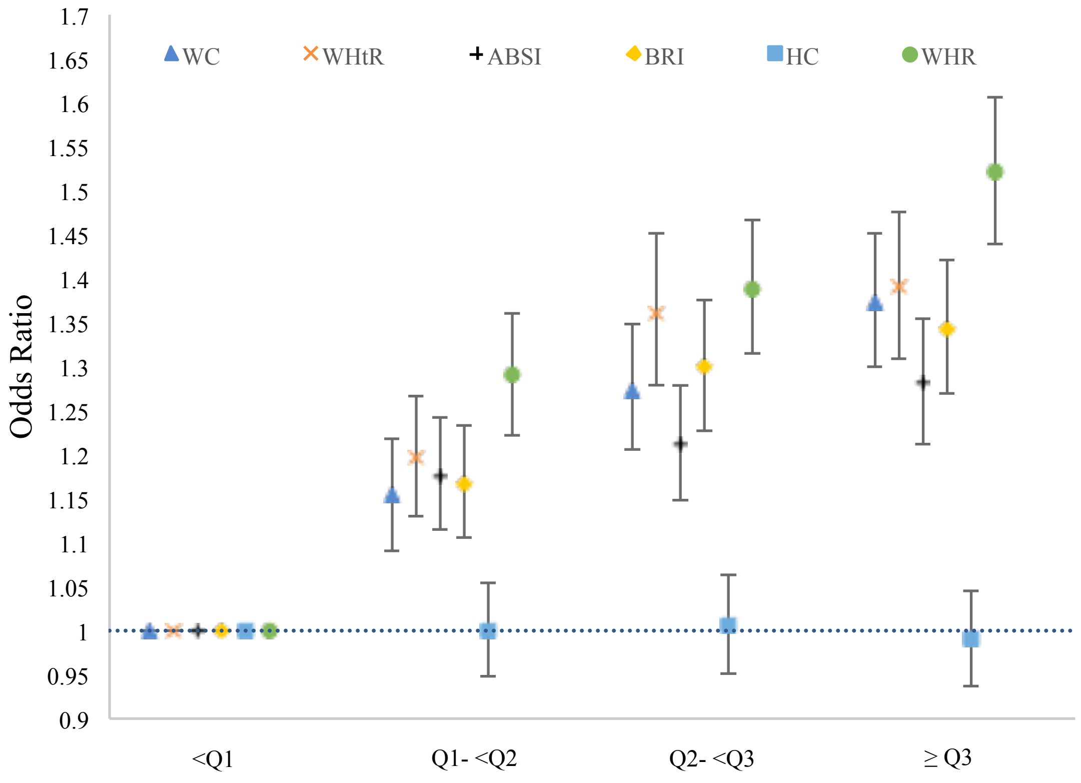

D2. OR for hypertriglyceridemia and anthropometric indices among obese male

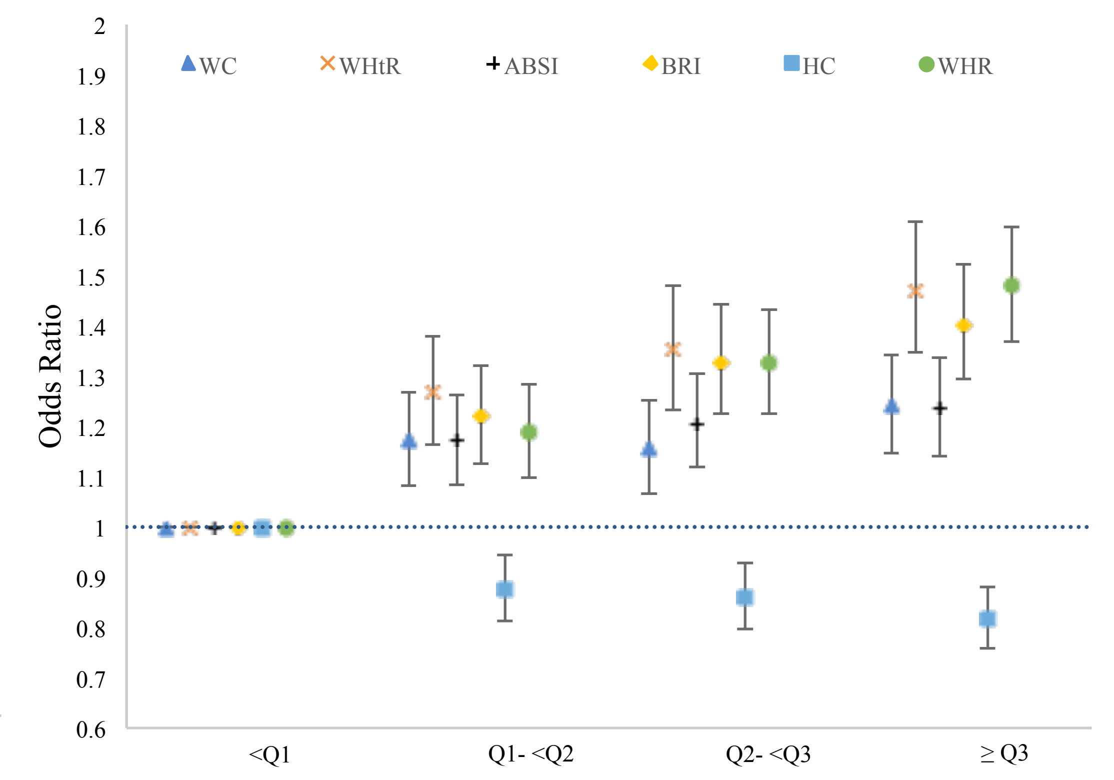

D3. OR for mixed hyperlipidemia and anthropometric indices among obese male

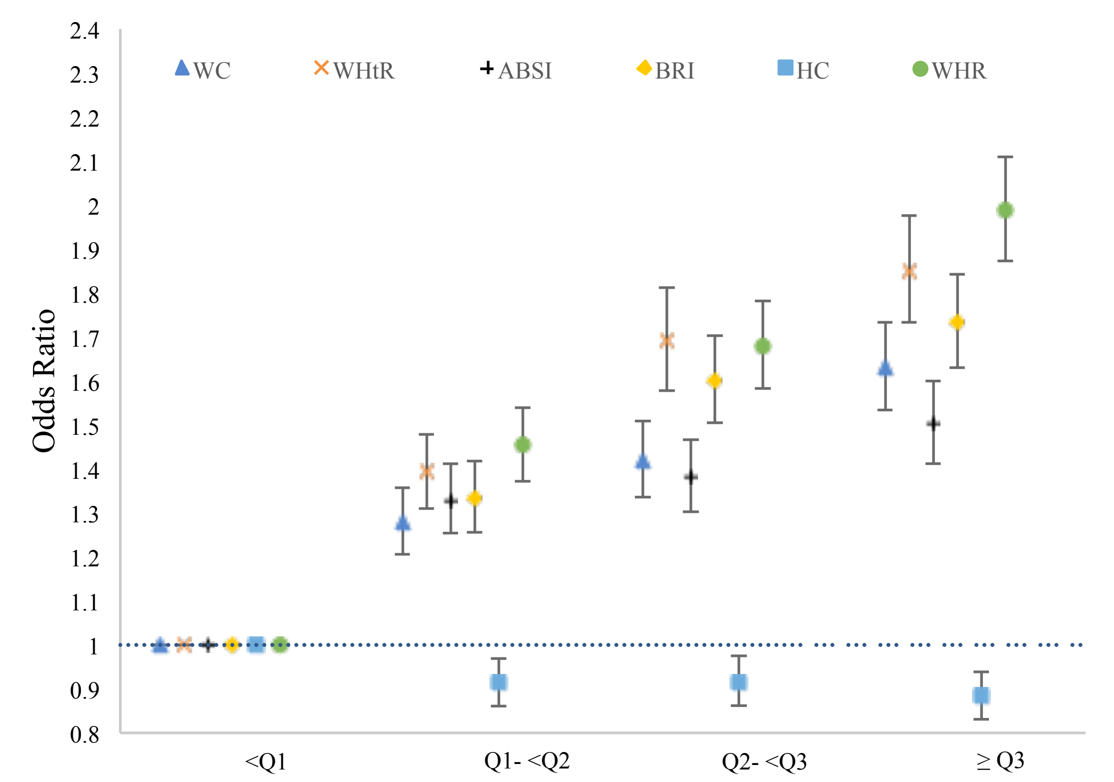

D4. OR for dyslipidemia and anthropometric indices among obese male

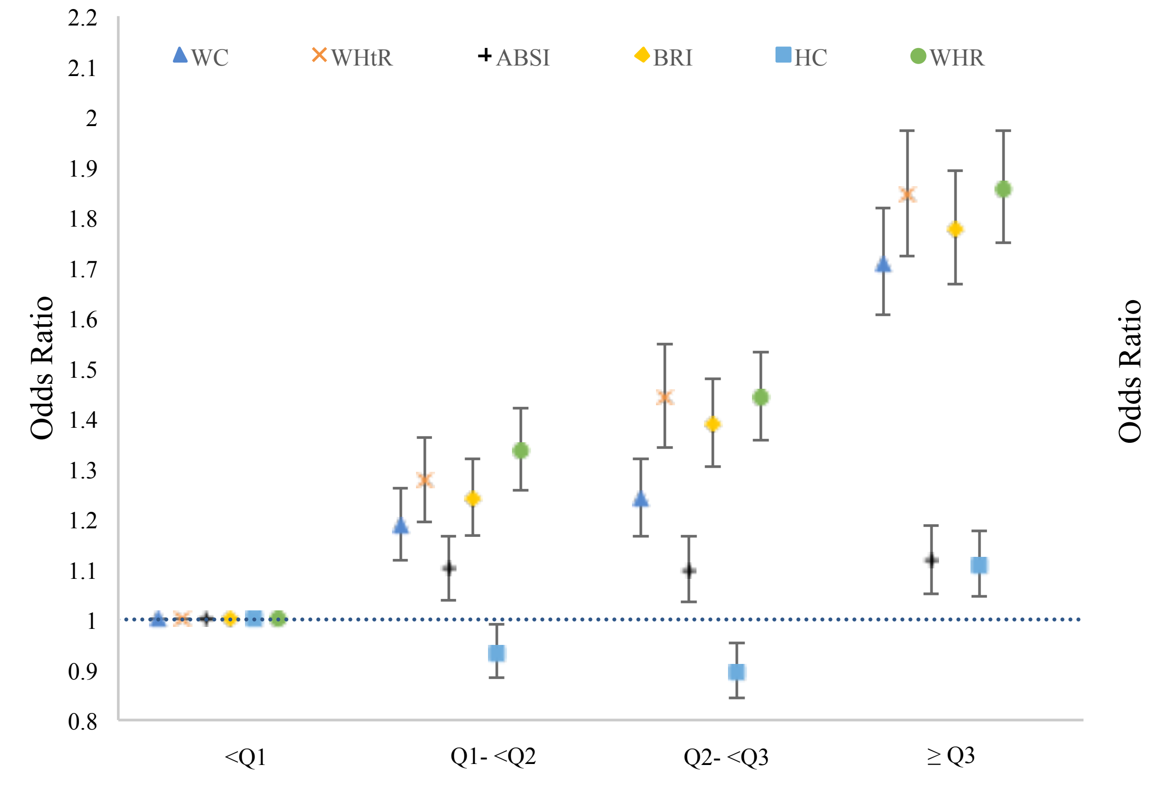

D5. OR for hypertension and anthropometric indices among obese male

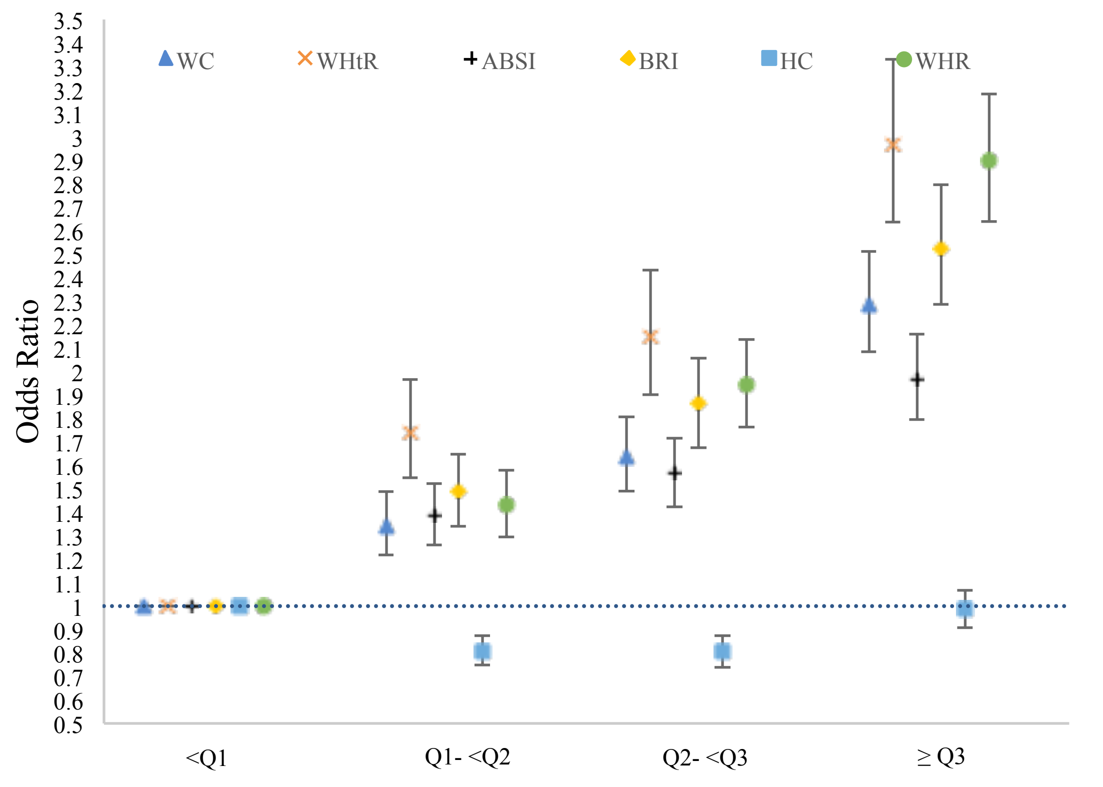

D6. OR for diabetic mellitus and anthropometric indices among obese male

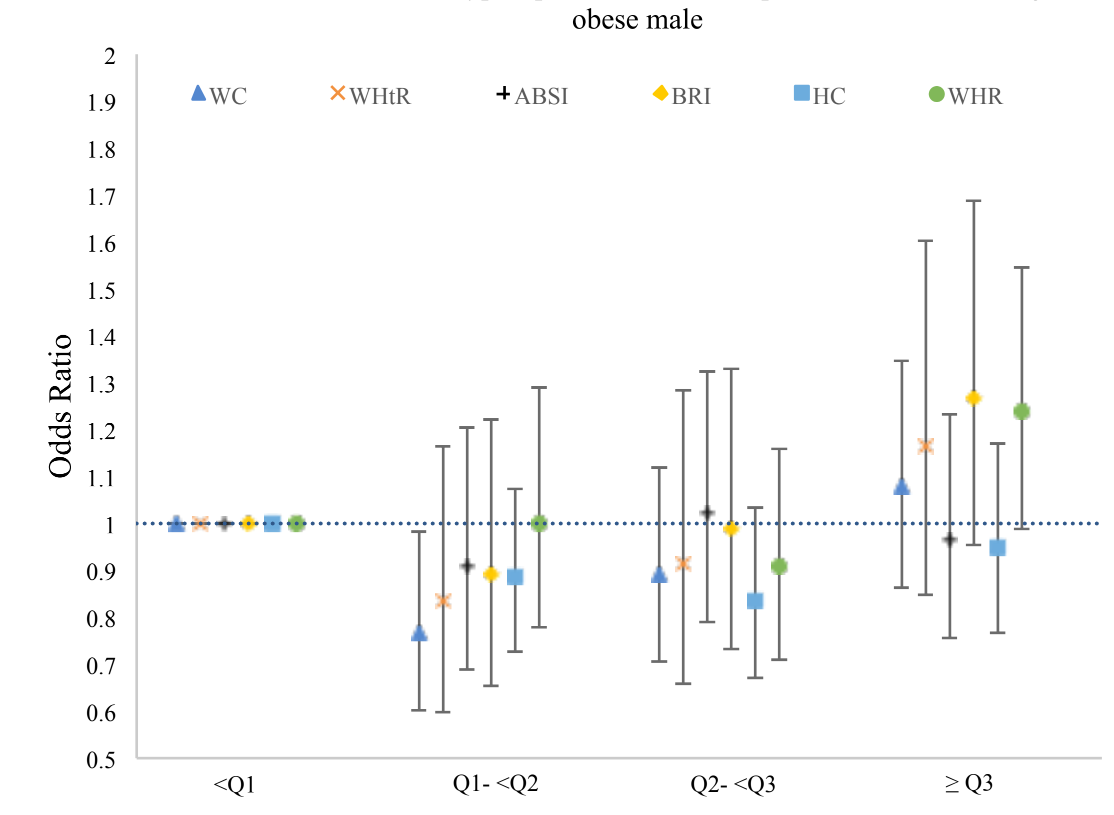

D7. OR for chronic kidney disease and anthropometric indices among obese male

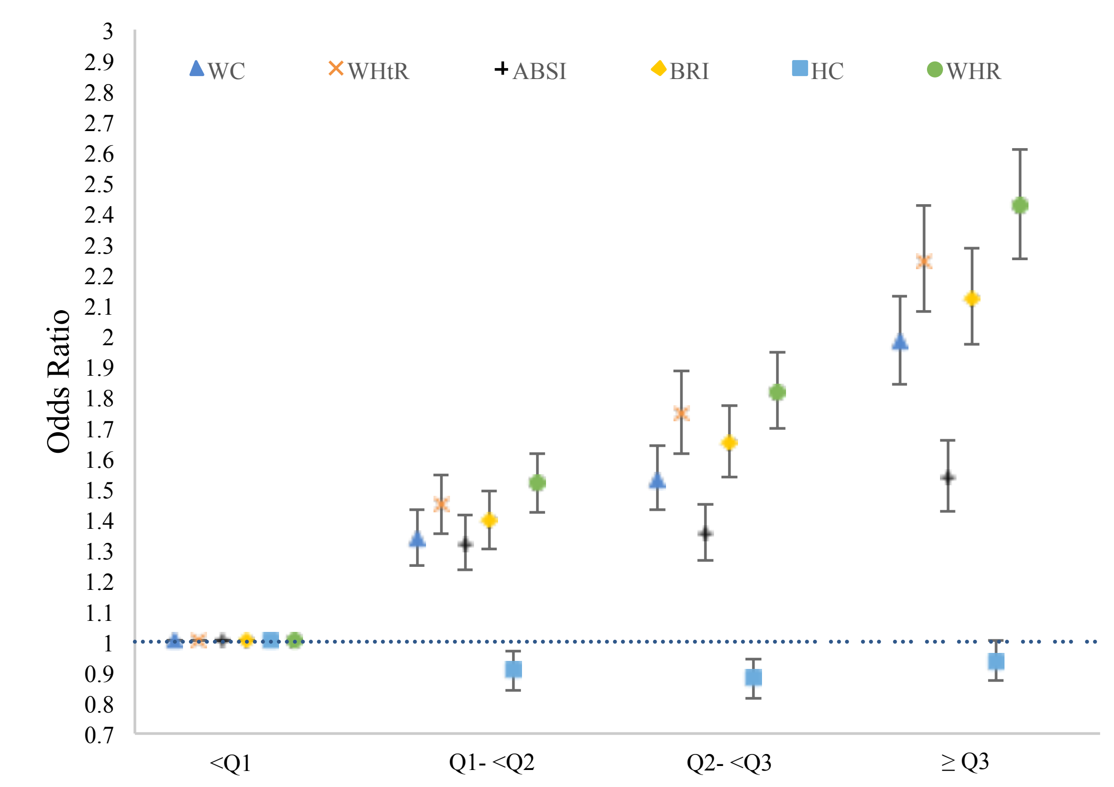

D8. OR for with at least one CVD risk and anthropometric indices among obese male

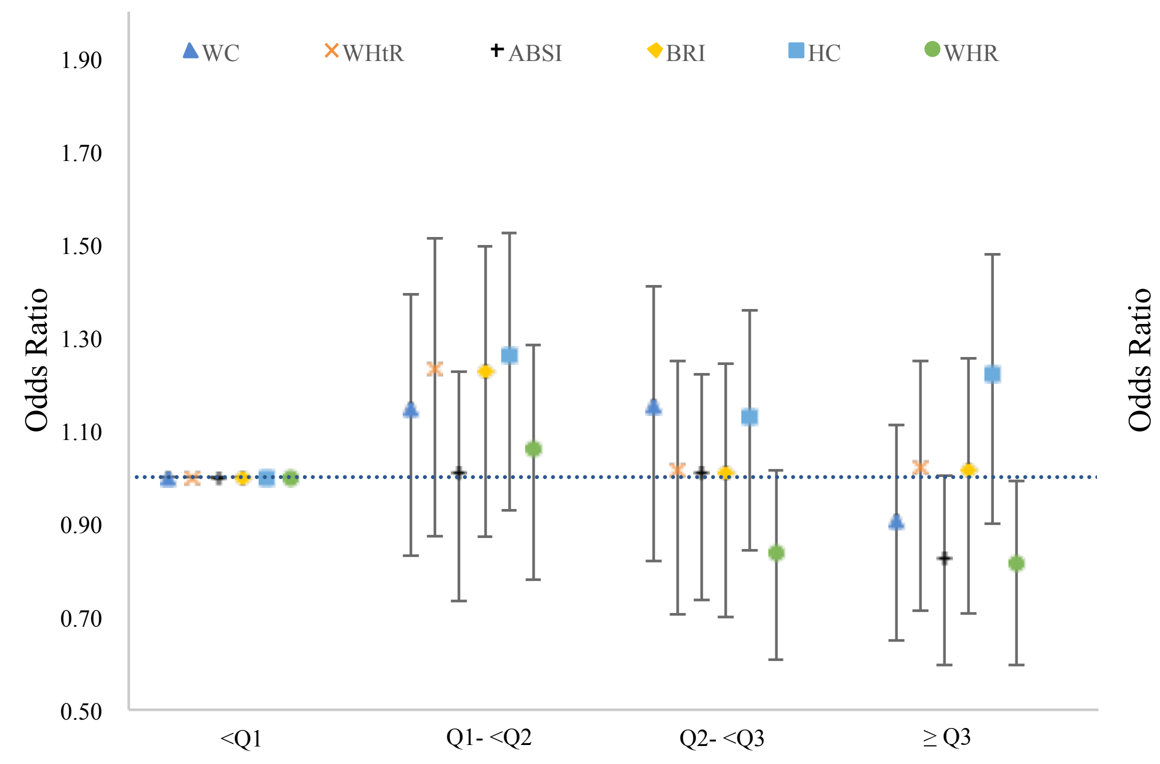

D9. OR for hypercholesterolemia and anthropometric indices among obese female

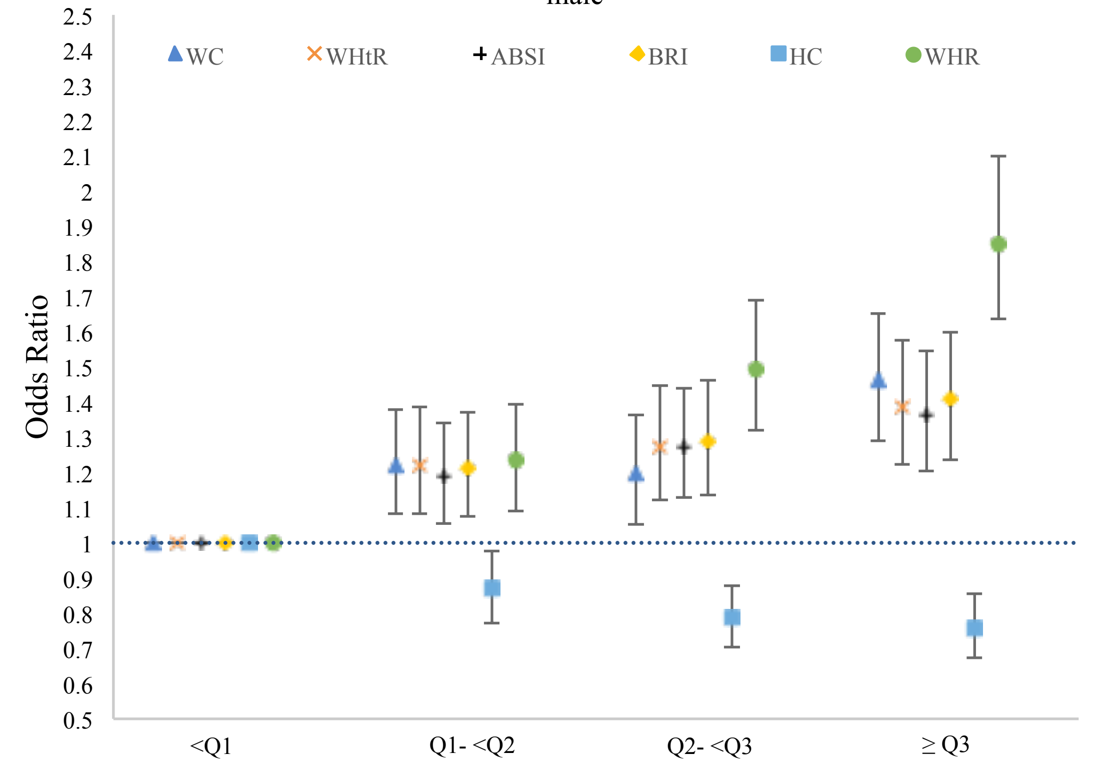

D10. OR for hypertriglyceridemia and anthropometric indices among obese female

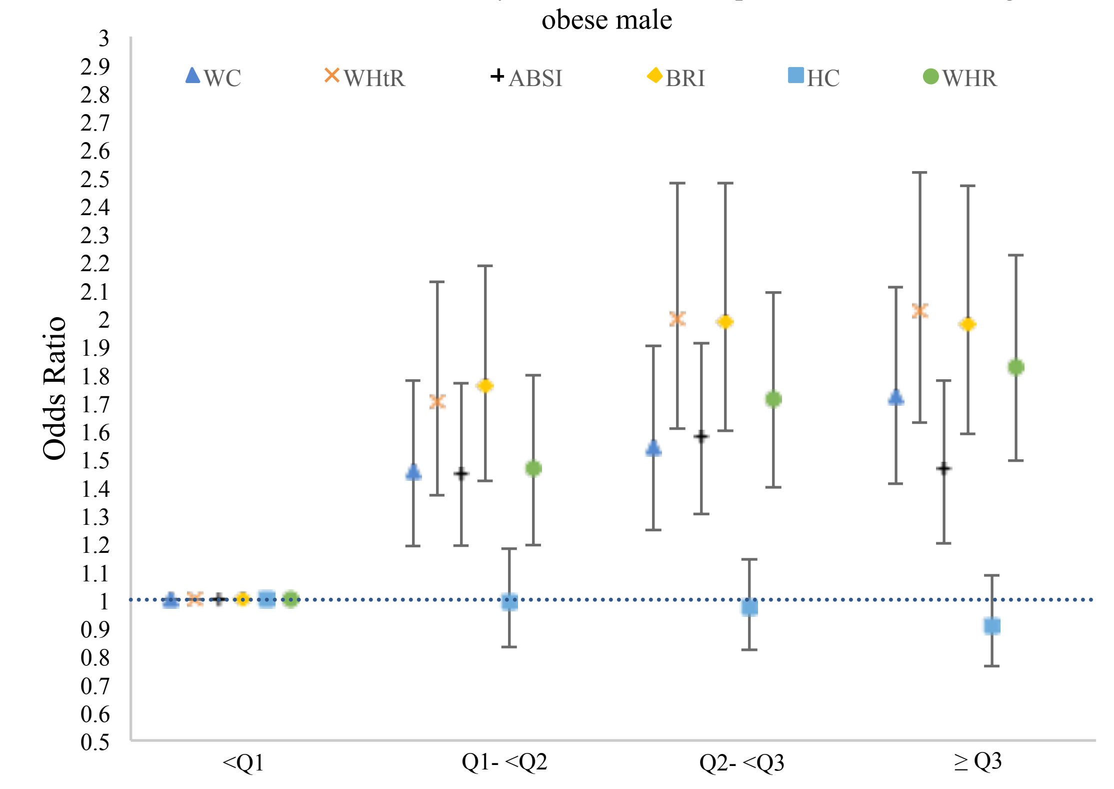

D11. OR for mixed hyperlipidemia and anthropometric indices among obese female

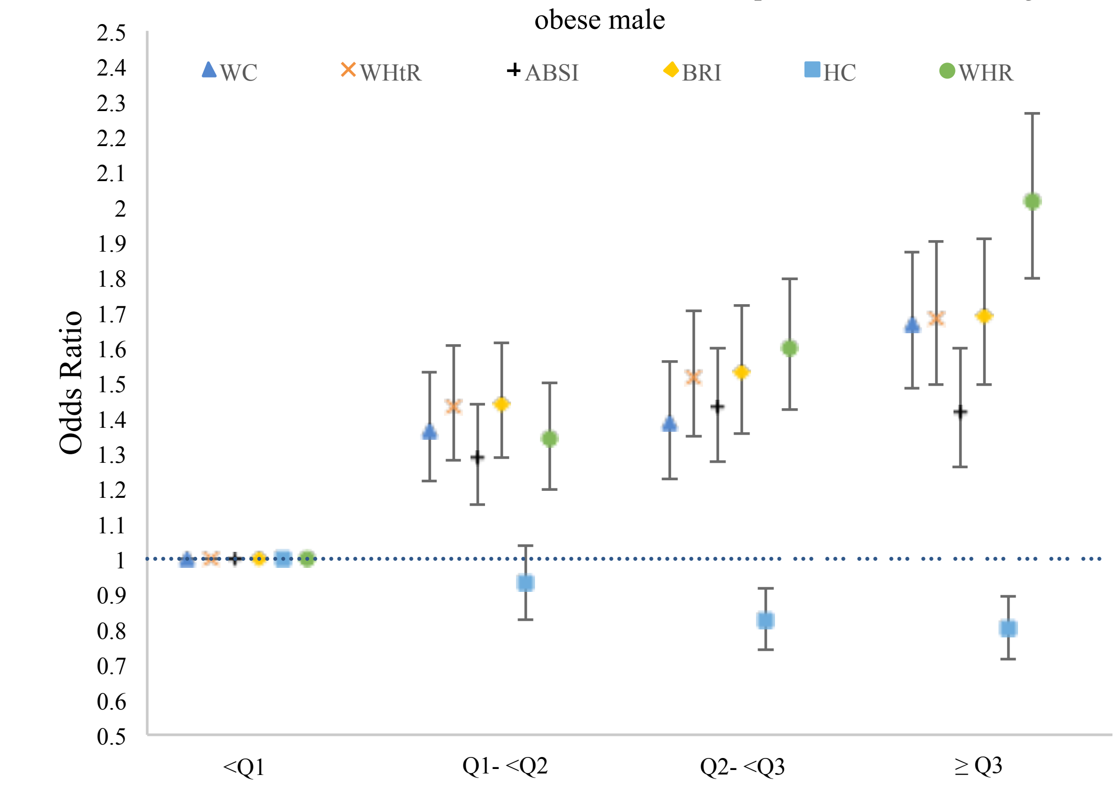

D12. OR for dyslipidemia and anthropometric indices among obese female

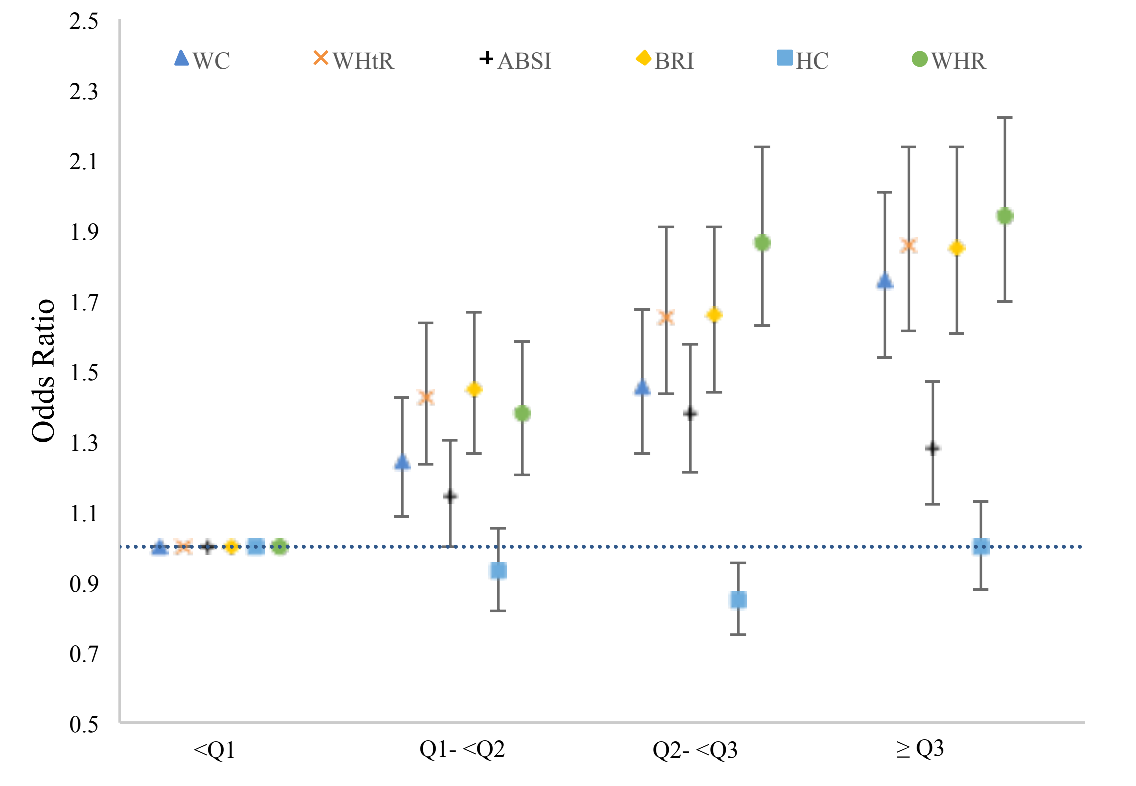

D13. OR for hypertension and anthropometric indices among obese female

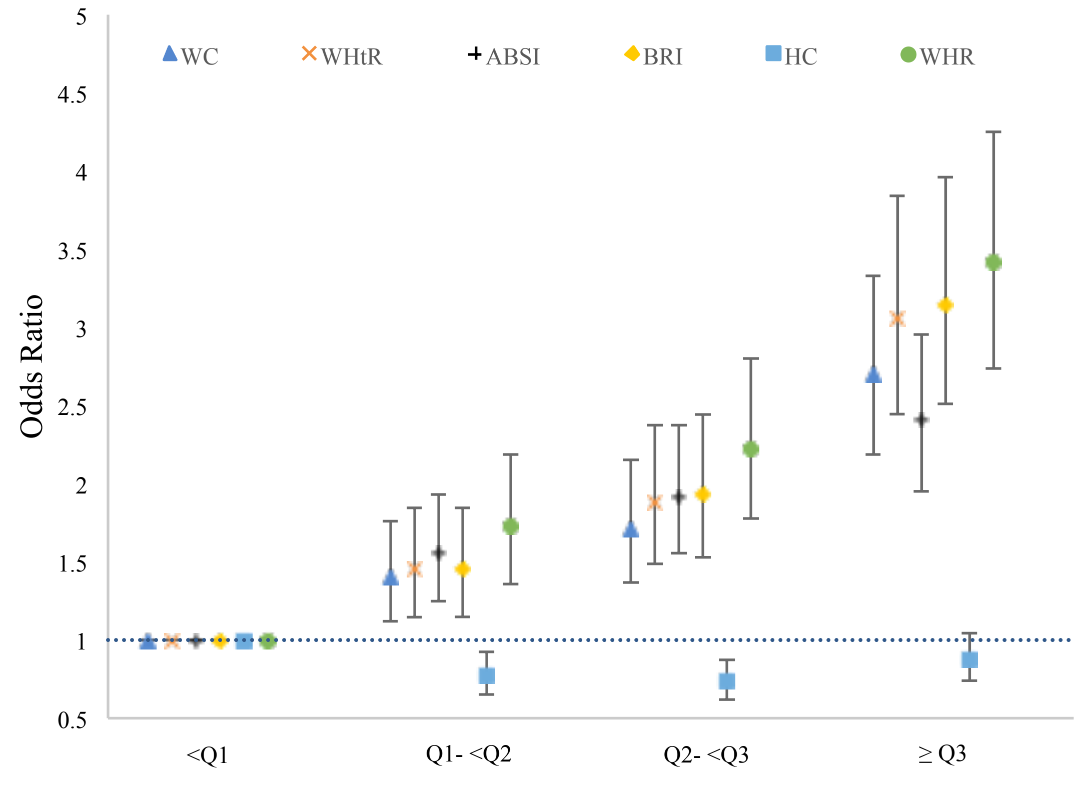

D14. OR for diabetic mellitus and anthropometric indices among obese female

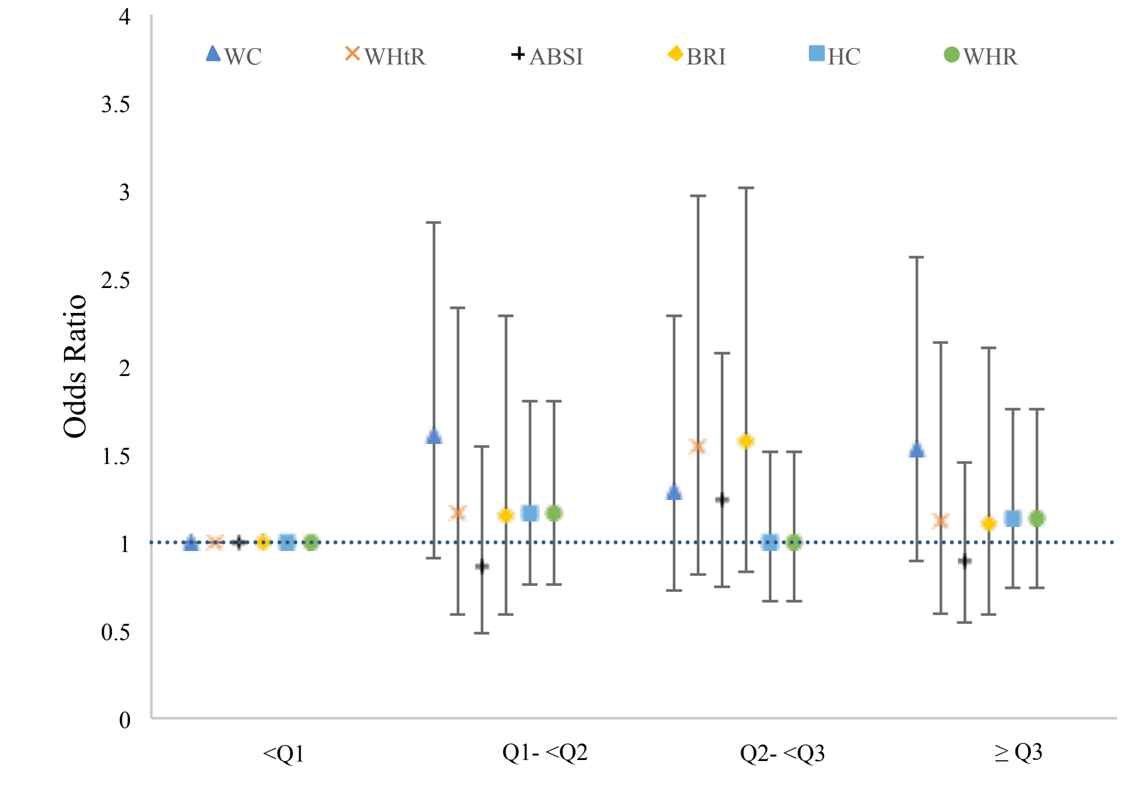

D15. OR for chronic kidney disease and anthropometric indices among obese female

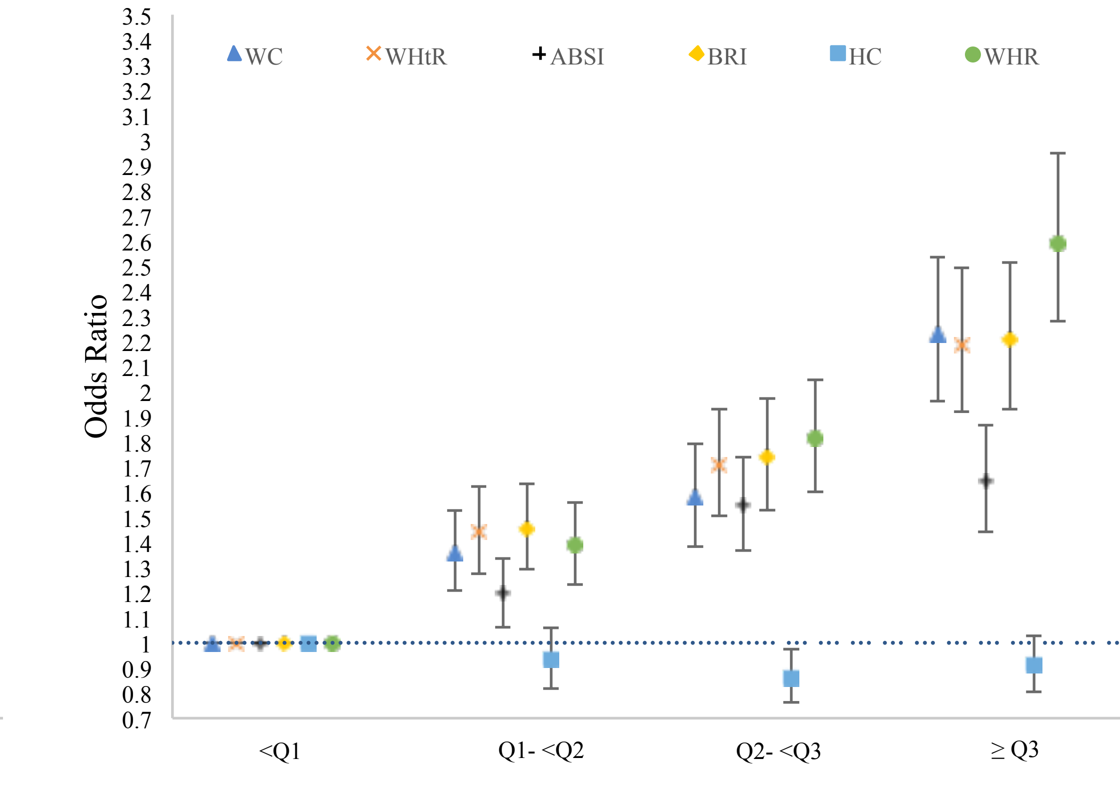

D168. OR for with at least one CVD risk and anthropometric indices among obese female

Figure S5. Association between CVD risk factors and anthropometric indices among obese Chinese *

* association was adjusted for age, smoking, and drinking status and index at <Q1 level was treated as non-exposed;
Abbreviation: Q1, first quartile, the 25th percentile; Q2: second quartile, median; Q3, third quartile, the 75th percentile; WC, waist circumstance; WHtR, waist height ratio; ABSI, a body shape index; BRI, body round index; HC, hip circumstance; WHR, waist hip ratio.

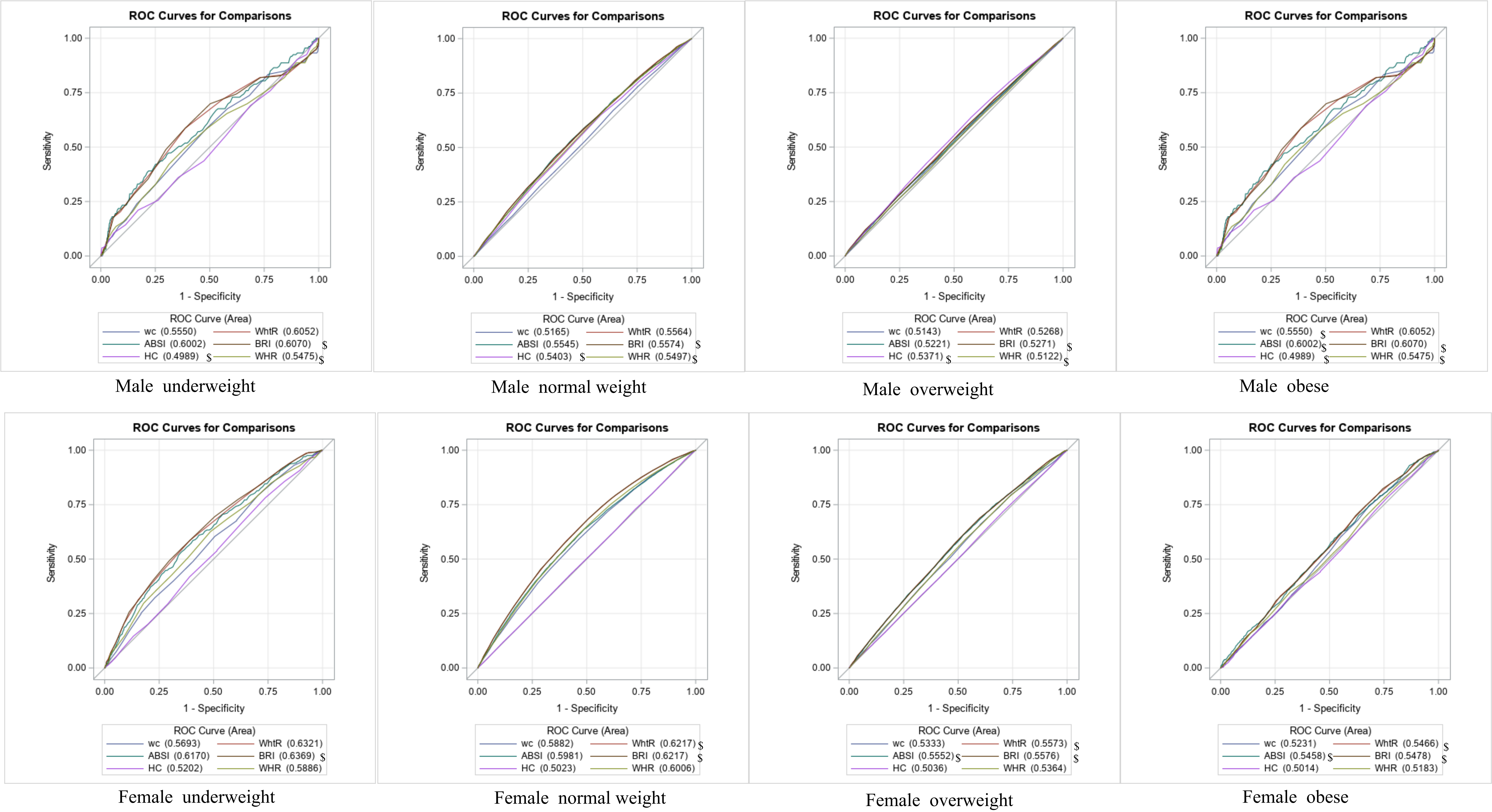


Figure S6. Comparison on area under curves for diagnosing hypercholesterolemia between different waist and hip indices within each BMI category

* Comparison on the area under different indices curves was based on Delong test; $ mean that AUROC of those indices were similar to each other (*P*>0.05) for identifying hypercholesterolemia within the specific BMI category, and meanwhile their AUROC were the highest in the six anthropometric indices(*P*<0.05).

Abbreviation: AUROC, area under receiver operator curves; ROC, receiver operator curves; BMI, body mass index;WC, waist circumstance; WHtR, waist height ratio; ABSI, a body shape index; BRI, body round index; HC, hip circumstance; WHR, waist hip ratio.

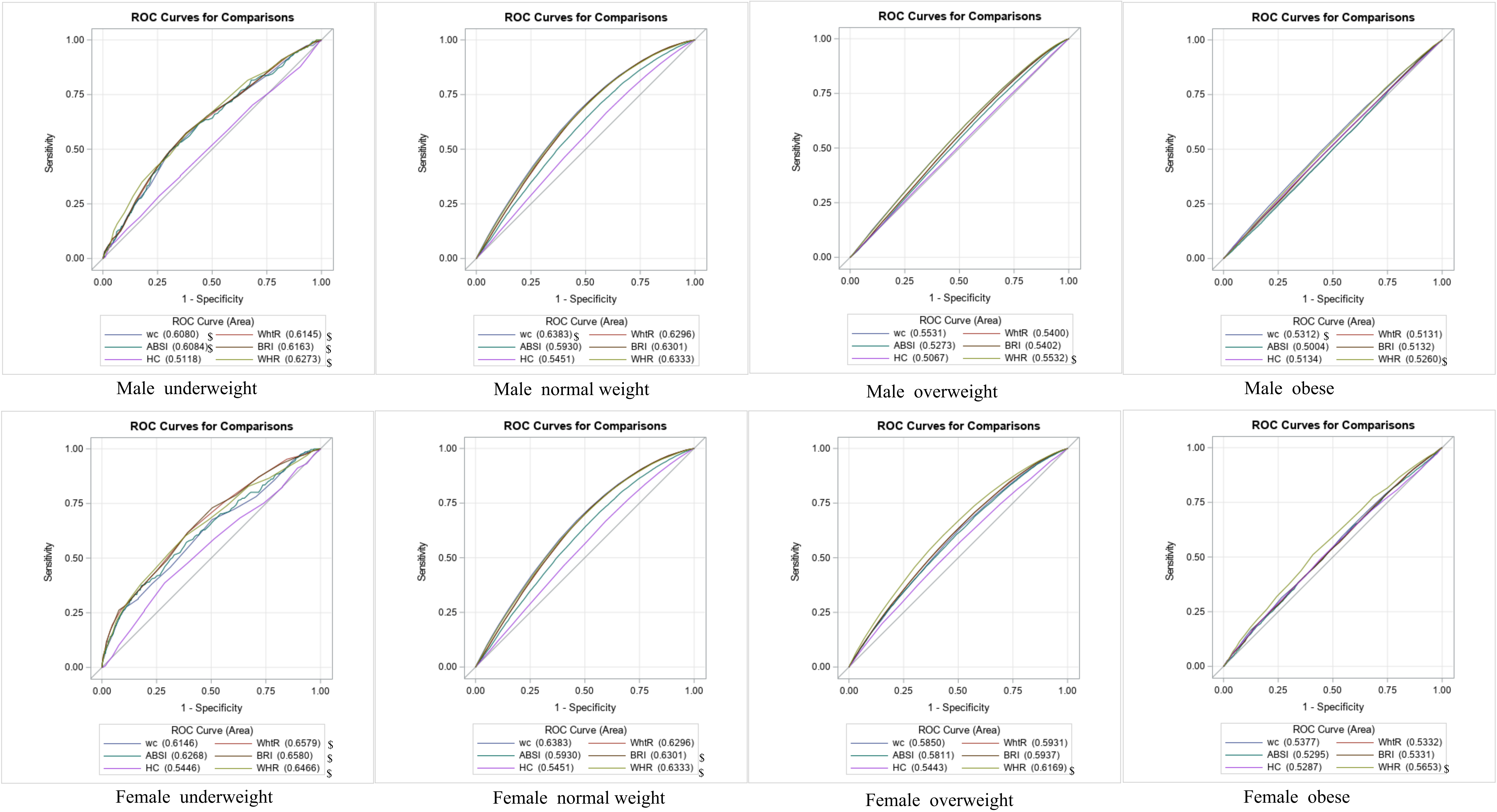


Figure S7. Comparison on area under curves for diagnosing hypertriglyceridemia between different waist and hip indices within each BMI category

* Comparison on the area under different indices curves was based on Delong test; $ mean that AUROC of those indices were similar to each other (*P*>0.05) for identifying hypertriglyceridemia within the specific BMI category, and meanwhile their AUROC were the highest in the six anthropometric indices(*P*<0.05).

Abbreviation: AUROC, area under receiver operator curves; ROC, receiver operator curves; BMI, body mass index;WC, waist circumstance; WHtR, waist height ratio; ABSI, a body shape index; BRI, body round index; HC, hip circumstance; WHR, waist hip ratio.

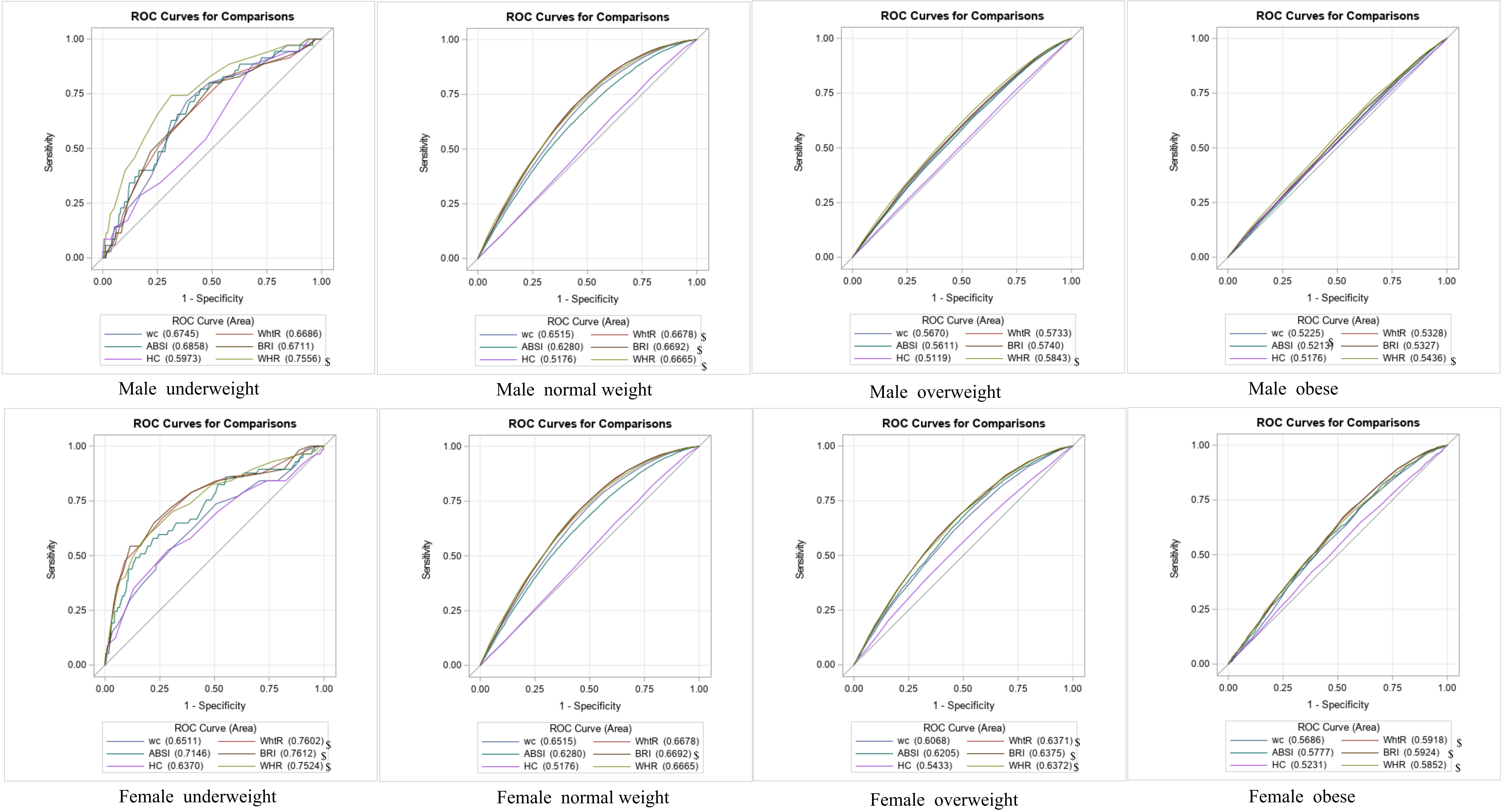


Figure S8. Comparison on area under curves for diagnosing mixed hyperlipidemia between different waist and hip indices within each BMI category

* Comparison on the area under different indices curves was based on Delong test; $ mean that AUROC of those indices were similar to each other (*P*>0.05) for identifying mixed hyperlipidemia within the specific BMI category, and meanwhile their AUROC were the highest in the six anthropometric indices(*P*<0.05).

Abbreviation: AUROC, area under receiver operator curves; ROC, receiver operator curves; BMI, body mass index;WC, waist circumstance; WHtR, waist height ratio; ABSI, a body shape index; BRI, body round index; HC, hip circumstance; WHR, waist hip ratio.

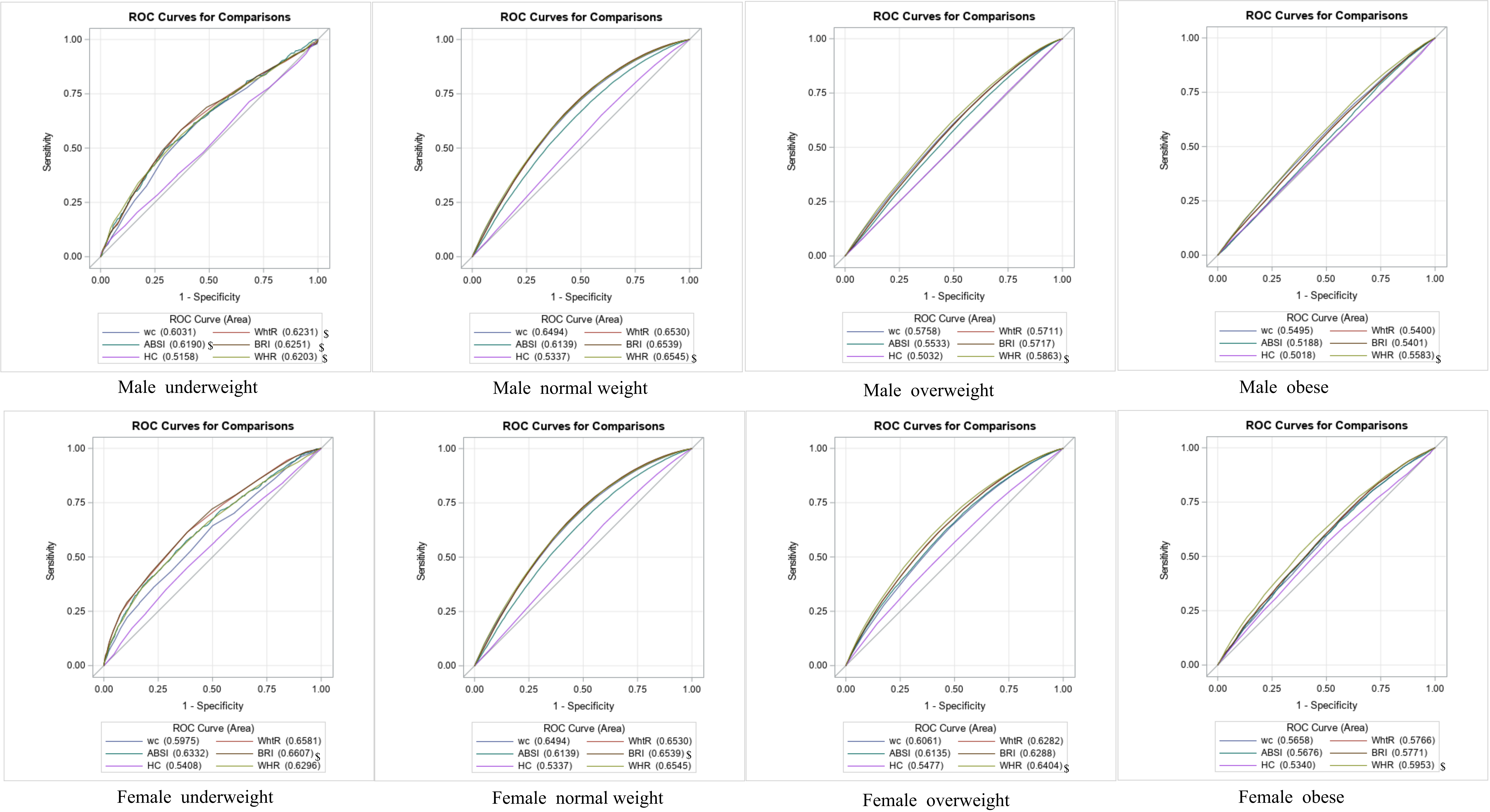


Figure S9. Comparison on area under curves for diagnosing dyslipidemia between different waist and hip indices within each BMI category

* Comparison on the area under different indices curves was based on Delong test; $ mean that AUROC of those indices were similar to each other (*P*>0.05) for identifying dyslipidemia within the specific BMI category, and meanwhile their AUROC were the highest in the six anthropometric indices(*P*<0.05).

Abbreviation: AUROC, area under receiver operator curves; ROC, receiver operator curves; BMI, body mass index;WC, waist circumstance; WHtR, waist height ratio; ABSI, a body shape index; BRI, body round index; HC, hip circumstance; WHR, waist hip ratio.

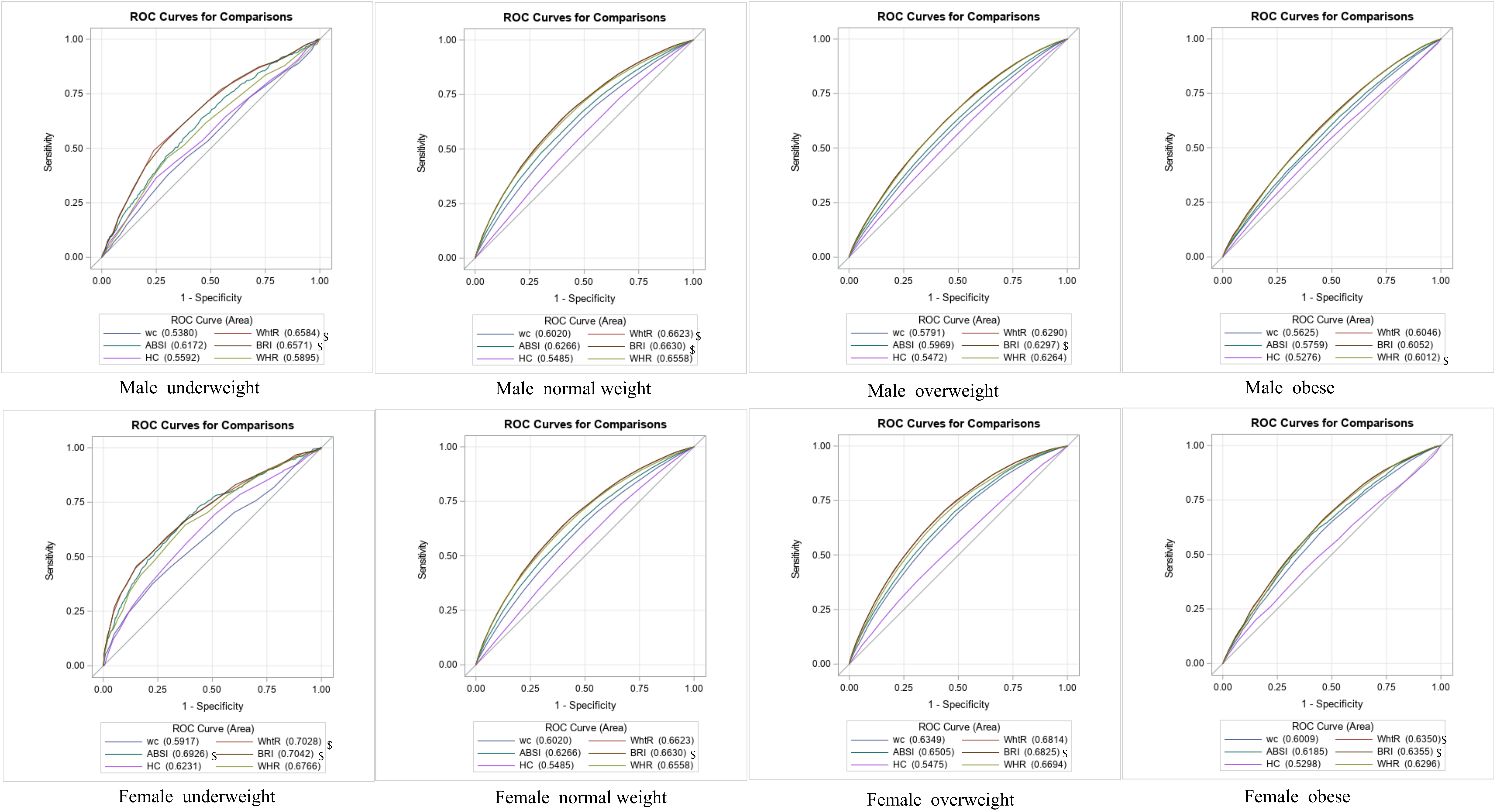


Figure S10. Comparison on area under curves for diagnosing hypertension between different waist and hip indices within each BMI category

* Comparison on the area under different indices curves was based on Delong test; $ mean that AUROC of those indices were similar to each other (*P*>0.05) for identifying hypertension within the specific BMI category, and meanwhile their AUROC were the highest in the six anthropometric indices(*P*<0.05).

Abbreviation: AUROC, area under receiver operator curves; ROC, receiver operator curves; BMI, body mass index;WC, waist circumstance; WHtR, waist height ratio; ABSI, a body shape index; BRI, body round index; HC, hip circumstance; WHR, waist hip ratio.

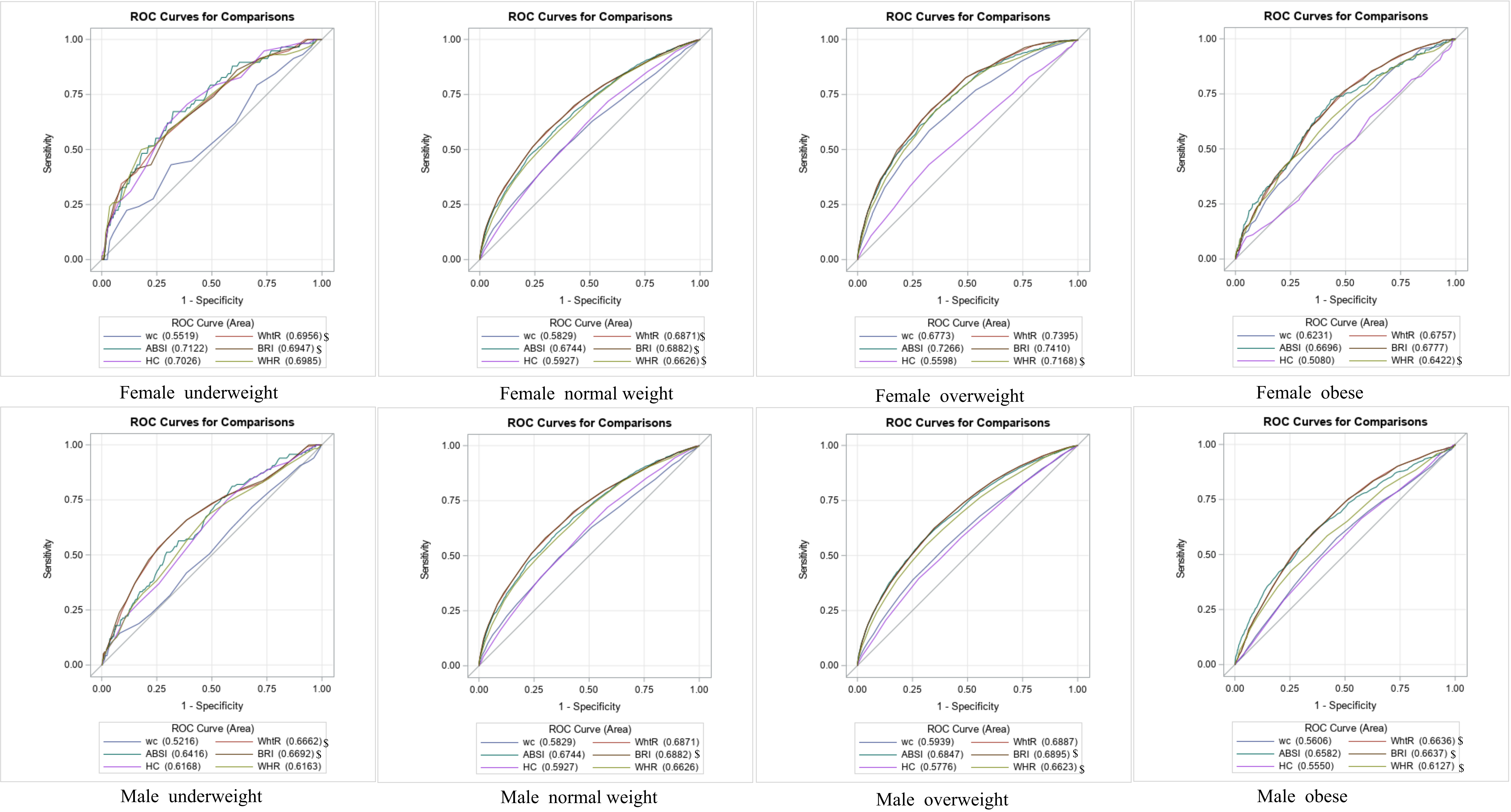


Figure S11. Comparison on area under curves for diagnosing chronic kidney disease between different waist and hip indices within each BMI category

* Comparison on the area under different indices curves was based on Delong test; $ mean that AUROC of those indices were similar to each other ($P$>0.05) for identifying chronic kidney disease within the specific BMI category, and meanwhile their AUROC were the highest in the six anthropometric indices($P$<0.05).

Abbreviation: AUROC, area under receiver operator curves; ROC, receiver operator curves; BMI, body mass index;WC, waist circumstance; WHtR, waist height ratio; ABSI, a body shape index; BRI, body round index; HC, hip circumstance; WHR, waist hip ratio.

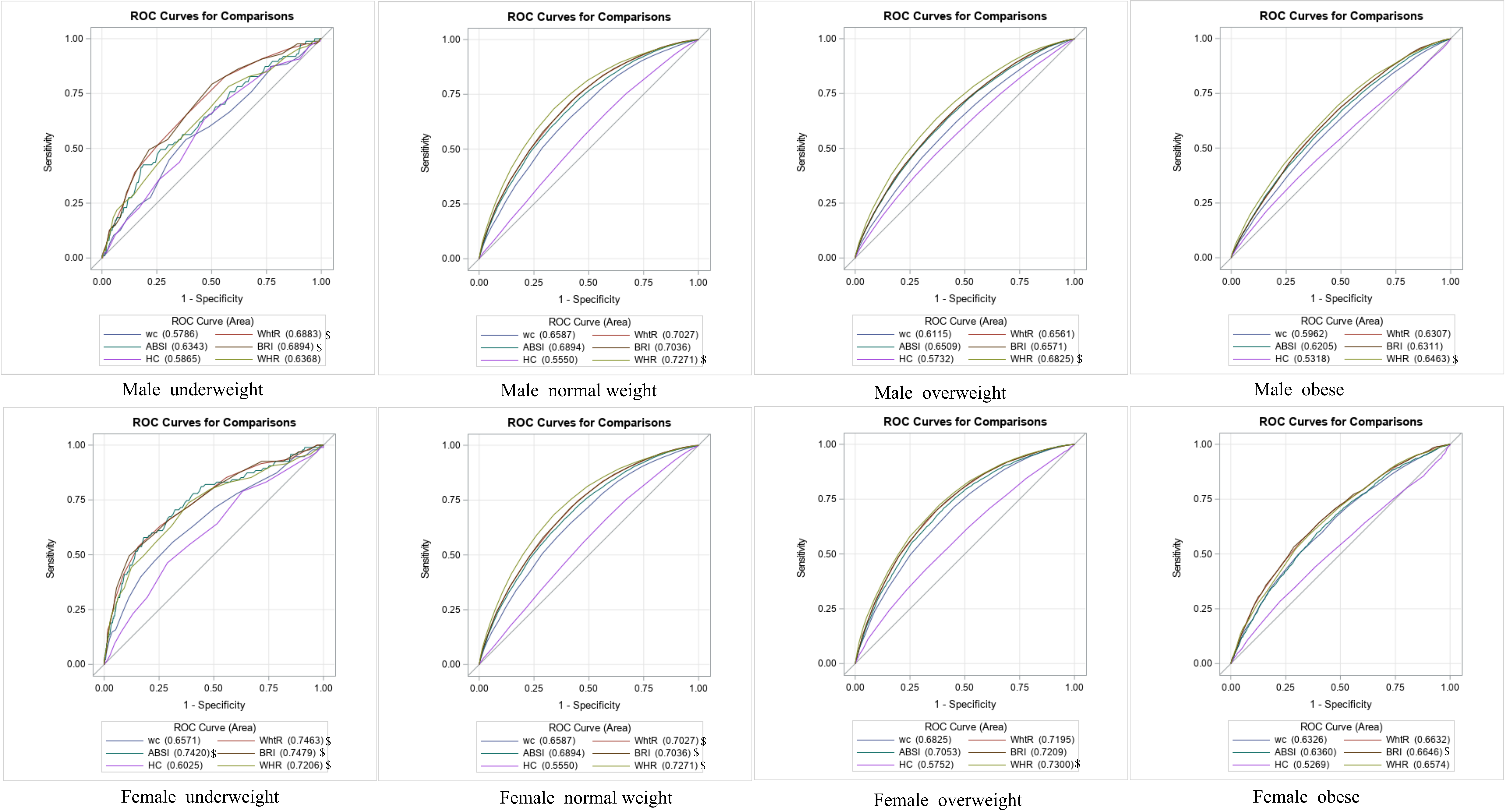


Figure S12. Comparison on area under curves for diagnosing diabetic mellitus between different waist and hip indices within each BMI category

* Comparison on the area under different indices curves was based on Delong test; $ mean that AUROC of those indices were similar to each other (*P*>0.05) for identifying diabetic mellitus within the specific BMI category, and meanwhile their AUROC were the highest in the six anthropometric indices(*P*<0.05).

Abbreviation: AUROC, area under receiver operator curves; ROC, receiver operator curves; BMI, body mass index; WC, waist circumstance; WHtR, waist height ratio; ABSI, a body shape index; BRI, body round index; HC, hip circumstance; WHR, waist hip ratio.

Waist circumstance(CM)

| Height(CM) | 60 | 62 | 64 | 66 | 68 | 70 | 72 | 74 | 76 | 78 | 80 | 82 | 84 | 86 | 88 | 90 | 92 | 94 | 96 | 98 | 100 | 102 | 104 | 106 | 108 | 110 | 112 |
|---|---|---|---|---|---|---|---|---|---|---|---|---|---|---|---|---|---|---|---|---|---|---|---|---|---|---|---|
| 146 | 1.841 | 2.055 | 2.276 | 2.504 | 2.739 | 2.982 | 3.231 | 3.488 | 3.752 | 4.024 | 4.302 | 4.588 | 4.882 | 5.182 | 5.490 | 5.805 | 6.128 | 6.458 | 6.795 | 7.140 | 7.492 | 7.852 | 8.219 | 8.594 | 8.977 | 9.366 | 9.764 |
| 148 | 1.756 | 1.964 | 2.179 | 2.401 | 2.630 | 2.866 | 3.109 | 3.359 | 3.616 | 3.880 | 4.151 | 4.429 | 4.714 | 5.007 | 5.306 | 5.613 | 5.926 | 6.247 | 6.576 | 6.911 | 7.254 | 7.603 | 7.961 | 8.325 | 8.697 | 9.076 | 9.462 |
| 150 | 1.675 | 1.877 | 2.087 | 2.303 | 2.525 | 2.755 | 2.991 | 3.235 | 3.485 | 3.742 | 4.005 | 4.276 | 4.554 | 4.838 | 5.129 | 5.428 | 5.733 | 6.045 | 6.365 | 6.691 | 7.024 | 7.365 | 7.712 | 8.067 | 8.428 | 8.797 | 9.173 |
| 152 | 1.597 | 1.794 | 1.998 | 2.208 | 2.425 | 2.648 | 2.879 | 3.115 | 3.359 | 3.609 | 3.866 | 4.129 | 4.399 | 4.676 | 4.960 | 5.250 | 5.548 | 5.851 | 6.162 | 6.480 | 6.804 | 7.136 | 7.474 | 7.819 | 8.171 | 8.530 | 8.895 |
| 154 | 1.522 | 1.714 | 1.912 | 2.117 | 2.328 | 2.546 | 2.770 | 3.001 | 3.238 | 3.481 | 3.731 | 3.988 | 4.251 | 4.521 | 4.797 | 5.080 | 5.369 | 5.665 | 5.968 | 6.277 | 6.593 | 6.915 | 7.245 | 7.580 | 7.923 | 8.273 | 8.629 |
| 156 | 1.449 | 1.637 | 1.830 | 2.029 | 2.235 | 2.447 | 2.666 | 2.891 | 3.122 | 3.359 | 3.602 | 3.852 | 4.109 | 4.371 | 4.640 | 4.916 | 5.198 | 5.486 | 5.781 | 6.082 | 6.390 | 6.704 | 7.024 | 7.351 | 7.685 | 8.025 | 8.372 |
| 158 | 1.380 | 1.562 | 1.751 | 1.945 | 2.146 | 2.353 | 2.566 | 2.785 | 3.010 | 3.241 | 3.478 | 3.722 | 3.972 | 4.228 | 4.490 | 4.758 | 5.033 | 5.314 | 5.601 | 5.894 | 6.194 | 6.500 | 6.813 | 7.131 | 7.456 | 7.788 | 8.126 |
| 160 | 1.313 | 1.491 | 1.675 | 1.864 | 2.060 | 2.262 | 2.469 | 2.682 | 2.902 | 3.127 | 3.359 | 3.596 | 3.840 | 4.089 | 4.345 | 4.606 | 4.874 | 5.148 | 5.428 | 5.714 | 6.006 | 6.304 | 6.609 | 6.919 | 7.236 | 7.559 | 7.889 |
| 162 | 1.249 | 1.422 | 1.601 | 1.786 | 1.977 | 2.174 | 2.376 | 2.584 | 2.798 | 3.018 | 3.244 | 3.475 | 3.713 | 3.956 | 4.205 | 4.460 | 4.721 | 4.988 | 5.261 | 5.540 | 5.825 | 6.116 | 6.413 | 6.715 | 7.024 | 7.339 | 7.660 |
| 164 | 1.187 | 1.356 | 1.531 | 1.711 | 1.897 | 2.089 | 2.287 | 2.490 | 2.698 | 2.913 | 3.133 | 3.359 | 3.590 | 3.828 | 4.071 | 4.320 | 4.574 | 4.835 | 5.101 | 5.373 | 5.651 | 5.934 | 6.224 | 6.519 | 6.820 | 7.127 | 7.440 |
| 166 | 1.127 | 1.292 | 1.463 | 1.639 | 1.820 | 2.008 | 2.200 | 2.398 | 2.602 | 2.811 | 3.026 | 3.247 | 3.472 | 3.704 | 3.941 | 4.184 | 4.432 | 4.686 | 4.946 | 5.211 | 5.483 | 5.759 | 6.042 | 6.330 | 6.624 | 6.923 | 7.229 |
| 168 | 1.069 | 1.231 | 1.397 | 1.569 | 1.746 | 1.929 | 2.117 | 2.310 | 2.509 | 2.713 | 2.923 | 3.138 | 3.359 | 3.585 | 3.816 | 4.053 | 4.296 | 4.544 | 4.797 | 5.056 | 5.320 | 5.591 | 5.866 | 6.147 | 6.434 | 6.726 | 7.024 |
| 170 | 1.014 | 1.171 | 1.334 | 1.502 | 1.675 | 1.853 | 2.037 | 2.226 | 2.420 | 2.619 | 2.824 | 3.034 | 3.249 | 3.470 | 3.696 | 3.927 | 4.164 | 4.406 | 4.653 | 4.906 | 5.164 | 5.428 | 5.697 | 5.971 | 6.251 | 6.537 | 6.827 |
| 172 | 0.960 | 1.114 | 1.273 | 1.437 | 1.606 | 1.780 | 1.959 | 2.144 | 2.333 | 2.528 | 2.728 | 2.933 | 3.143 | 3.359 | 3.579 | 3.805 | 4.037 | 4.273 | 4.514 | 4.761 | 5.013 | 5.271 | 5.534 | 5.802 | 6.075 | 6.353 | 6.637 |
| 174 | 0.908 | 1.059 | 1.214 | 1.374 | 1.539 | 1.709 | 1.884 | 2.065 | 2.250 | 2.440 | 2.635 | 2.836 | 3.041 | 3.252 | 3.467 | 3.688 | 3.914 | 4.145 | 4.381 | 4.622 | 4.868 | 5.119 | 5.376 | 5.638 | 5.905 | 6.177 | 6.454 |
| 176 | 0.858 | 1.005 | 1.157 | 1.313 | 1.475 | 1.641 | 1.812 | 1.988 | 2.169 | 2.355 | 2.546 | 2.742 | 2.942 | 3.148 | 3.359 | 3.574 | 3.795 | 4.021 | 4.251 | 4.487 | 4.727 | 4.973 | 5.224 | 5.479 | 5.740 | 6.006 | 6.277 |
| 178 | 0.810 | 0.953 | 1.102 | 1.255 | 1.412 | 1.575 | 1.742 | 1.914 | 2.091 | 2.273 | 2.460 | 2.651 | 2.847 | 3.048 | 3.254 | 3.465 | 3.680 | 3.901 | 4.126 | 4.356 | 4.592 | 4.832 | 5.077 | 5.326 | 5.581 | 5.841 | 6.106 |
| 180 | 0.763 | 0.903 | 1.048 | 1.198 | 1.352 | 1.511 | 1.675 | 1.843 | 2.016 | 2.194 | 2.376 | 2.563 | 2.755 | 2.952 | 3.153 | 3.359 | 3.570 | 3.785 | 4.005 | 4.230 | 4.460 | 4.695 | 4.934 | 5.179 | 5.428 | 5.682 | 5.941 |
| 182 | 0.718 | 0.855 | 0.997 | 1.143 | 1.294 | 1.449 | 1.609 | 1.774 | 1.943 | 2.117 | 2.295 | 2.478 | 2.666 | 2.858 | 3.055 | 3.256 | 3.462 | 3.673 | 3.889 | 4.109 | 4.333 | 4.563 | 4.797 | 5.036 | 5.279 | 5.528 | 5.781 |
| 184 | 0.674 | 0.808 | 0.947 | 1.090 | 1.238 | 1.390 | 1.546 | 1.707 | 1.873 | 2.043 | 2.217 | 2.396 | 2.580 | 2.768 | 2.960 | 3.157 | 3.359 | 3.565 | 3.776 | 3.991 | 4.211 | 4.435 | 4.664 | 4.898 | 5.136 | 5.379 | 5.626 |
| 186 | 0.632 | 0.763 | 0.899 | 1.039 | 1.183 | 1.332 | 1.485 | 1.643 | 1.805 | 1.971 | 2.142 | 2.317 | 2.496 | 2.680 | 2.869 | 3.061 | 3.259 | 3.460 | 3.666 | 3.877 | 4.092 | 4.311 | 4.536 | 4.764 | 4.997 | 5.235 | 5.477 |
| 188 | 0.591 | 0.719 | 0.852 | 0.989 | 1.131 | 1.276 | 1.426 | 1.580 | 1.739 | 1.901 | 2.068 | 2.240 | 2.415 | 2.595 | 2.780 | 2.968 | 3.161 | 3.359 | 3.561 | 3.767 | 3.977 | 4.192 | 4.411 | 4.635 | 4.863 | 5.095 | 5.332 |
| 190 | 0.551 | 0.677 | 0.807 | 0.941 | 1.079 | 1.222 | 1.369 | 1.520 | 1.675 | 1.834 | 1.998 | 2.165 | 2.337 | 2.513 | 2.694 | 2.879 | 3.067 | 3.261 | 3.458 | 3.660 | 3.866 | 4.076 | 4.291 | 4.509 | 4.732 | 4.960 | 5.192 |

1.8≤BRI<3.0 3.0≤BRI<3.9 3.9≤BRI<5.0 BRI≥5.0

Figure S13. Body roundness index chart for high risk CVDRFs identification among male

BRI cut-off by BMI catergory for high risk CVDRFs identification among male :underweight(BMI<18.5) BRI≥1.8; normal weight(18.5≤BMI<24) BRI≥3.0; overweight (24≤BMI<28) BRI≥3.9; Obese (BMI≥28) BRI≥5.0

Waist circumstance(CM)

| Height(CM) | 60 | 62 | 64 | 66 | 68 | 70 | 72 | 74 | 76 | 78 | 80 | 82 | 84 | 86 | 88 | 90 | 92 | 94 | 96 | 98 | 100 | 102 | 104 | 106 | 108 | 110 | 112 |
|---|---|---|---|---|---|---|---|---|---|---|---|---|---|---|---|---|---|---|---|---|---|---|---|---|---|---|---|
| 146 | 1.841 | 2.055 | 2.276 | 2.504 | 2.739 | 2.982 | 3.231 | 3.488 | 3.752 | 4.024 | 4.302 | 4.588 | 4.882 | 5.182 | 5.490 | 5.805 | 6.128 | 6.458 | 6.795 | 7.140 | 7.492 | 7.852 | 8.219 | 8.594 | 8.977 | 9.366 | 9.764 |
| 148 | 1.756 | 1.964 | 2.179 | 2.401 | 2.630 | 2.866 | 3.109 | 3.359 | 3.616 | 3.880 | 4.151 | 4.429 | 4.714 | 5.007 | 5.306 | 5.613 | 5.926 | 6.247 | 6.576 | 6.911 | 7.254 | 7.603 | 7.961 | 8.325 | 8.697 | 9.076 | 9.462 |
| 150 | 1.675 | 1.877 | 2.087 | 2.303 | 2.525 | 2.755 | 2.991 | 3.235 | 3.485 | 3.742 | 4.005 | 4.276 | 4.554 | 4.838 | 5.129 | 5.428 | 5.733 | 6.045 | 6.365 | 6.691 | 7.024 | 7.365 | 7.712 | 8.067 | 8.428 | 8.797 | 9.173 |
| 152 | 1.597 | 1.794 | 1.998 | 2.208 | 2.425 | 2.648 | 2.879 | 3.115 | 3.359 | 3.609 | 3.866 | 4.129 | 4.399 | 4.676 | 4.960 | 5.250 | 5.548 | 5.851 | 6.162 | 6.480 | 6.804 | 7.136 | 7.474 | 7.819 | 8.171 | 8.530 | 8.895 |
| 154 | 1.522 | 1.714 | 1.912 | 2.117 | 2.328 | 2.546 | 2.770 | 3.001 | 3.238 | 3.481 | 3.731 | 3.988 | 4.251 | 4.521 | 4.797 | 5.080 | 5.369 | 5.665 | 5.968 | 6.277 | 6.593 | 6.915 | 7.245 | 7.580 | 7.923 | 8.273 | 8.629 |
| 156 | 1.449 | 1.637 | 1.830 | 2.029 | 2.235 | 2.447 | 2.666 | 2.891 | 3.122 | 3.359 | 3.602 | 3.852 | 4.109 | 4.371 | 4.640 | 4.916 | 5.198 | 5.486 | 5.781 | 6.082 | 6.390 | 6.704 | 7.024 | 7.351 | 7.685 | 8.025 | 8.372 |
| 158 | 1.380 | 1.562 | 1.751 | 1.945 | 2.146 | 2.353 | 2.566 | 2.785 | 3.010 | 3.241 | 3.478 | 3.722 | 3.972 | 4.228 | 4.490 | 4.758 | 5.033 | 5.314 | 5.601 | 5.894 | 6.194 | 6.500 | 6.813 | 7.131 | 7.456 | 7.788 | 8.126 |
| 160 | 1.313 | 1.491 | 1.675 | 1.864 | 2.060 | 2.262 | 2.469 | 2.682 | 2.902 | 3.127 | 3.359 | 3.596 | 3.840 | 4.089 | 4.345 | 4.606 | 4.874 | 5.148 | 5.428 | 5.714 | 6.006 | 6.304 | 6.609 | 6.919 | 7.236 | 7.559 | 7.889 |
| 162 | 1.249 | 1.422 | 1.601 | 1.786 | 1.977 | 2.174 | 2.376 | 2.584 | 2.798 | 3.018 | 3.244 | 3.475 | 3.713 | 3.956 | 4.205 | 4.460 | 4.721 | 4.988 | 5.261 | 5.540 | 5.825 | 6.116 | 6.413 | 6.715 | 7.024 | 7.339 | 7.660 |
| 164 | 1.187 | 1.356 | 1.531 | 1.711 | 1.897 | 2.089 | 2.287 | 2.490 | 2.698 | 2.913 | 3.133 | 3.359 | 3.590 | 3.828 | 4.071 | 4.320 | 4.574 | 4.835 | 5.101 | 5.373 | 5.651 | 5.934 | 6.224 | 6.519 | 6.820 | 7.127 | 7.440 |
| 166 | 1.127 | 1.292 | 1.463 | 1.639 | 1.820 | 2.008 | 2.200 | 2.398 | 2.602 | 2.811 | 3.026 | 3.247 | 3.472 | 3.704 | 3.941 | 4.184 | 4.432 | 4.686 | 4.946 | 5.211 | 5.483 | 5.759 | 6.042 | 6.330 | 6.624 | 6.923 | 7.229 |
| 168 | 1.069 | 1.231 | 1.397 | 1.569 | 1.746 | 1.929 | 2.117 | 2.310 | 2.509 | 2.713 | 2.923 | 3.138 | 3.359 | 3.585 | 3.816 | 4.053 | 4.296 | 4.544 | 4.797 | 5.056 | 5.320 | 5.591 | 5.866 | 6.147 | 6.434 | 6.726 | 7.024 |
| 170 | 1.014 | 1.171 | 1.334 | 1.502 | 1.675 | 1.853 | 2.037 | 2.226 | 2.420 | 2.619 | 2.824 | 3.034 | 3.249 | 3.470 | 3.696 | 3.927 | 4.164 | 4.406 | 4.653 | 4.906 | 5.164 | 5.428 | 5.697 | 5.971 | 6.251 | 6.537 | 6.827 |
| 172 | 0.960 | 1.114 | 1.273 | 1.437 | 1.606 | 1.780 | 1.959 | 2.144 | 2.333 | 2.528 | 2.728 | 2.933 | 3.143 | 3.359 | 3.579 | 3.805 | 4.037 | 4.273 | 4.514 | 4.761 | 5.013 | 5.271 | 5.534 | 5.802 | 6.075 | 6.353 | 6.637 |
| 174 | 0.908 | 1.059 | 1.214 | 1.374 | 1.539 | 1.709 | 1.884 | 2.065 | 2.250 | 2.440 | 2.635 | 2.836 | 3.041 | 3.252 | 3.467 | 3.688 | 3.914 | 4.145 | 4.381 | 4.622 | 4.868 | 5.119 | 5.376 | 5.638 | 5.905 | 6.177 | 6.454 |
| 176 | 0.858 | 1.005 | 1.157 | 1.313 | 1.475 | 1.641 | 1.812 | 1.988 | 2.169 | 2.355 | 2.546 | 2.742 | 2.942 | 3.148 | 3.359 | 3.574 | 3.795 | 4.021 | 4.251 | 4.487 | 4.727 | 4.973 | 5.224 | 5.479 | 5.740 | 6.006 | 6.277 |
| 178 | 0.810 | 0.953 | 1.102 | 1.255 | 1.412 | 1.575 | 1.742 | 1.914 | 2.091 | 2.273 | 2.460 | 2.651 | 2.847 | 3.048 | 3.254 | 3.465 | 3.680 | 3.901 | 4.126 | 4.356 | 4.592 | 4.832 | 5.077 | 5.326 | 5.581 | 5.841 | 6.106 |
| 180 | 0.763 | 0.903 | 1.048 | 1.198 | 1.352 | 1.511 | 1.675 | 1.843 | 2.016 | 2.194 | 2.376 | 2.563 | 2.755 | 2.952 | 3.153 | 3.359 | 3.570 | 3.785 | 4.005 | 4.230 | 4.460 | 4.695 | 4.934 | 5.179 | 5.428 | 5.682 | 5.941 |
| 182 | 0.718 | 0.855 | 0.997 | 1.143 | 1.294 | 1.449 | 1.609 | 1.774 | 1.943 | 2.117 | 2.295 | 2.478 | 2.666 | 2.858 | 3.055 | 3.256 | 3.462 | 3.673 | 3.889 | 4.109 | 4.333 | 4.563 | 4.797 | 5.036 | 5.279 | 5.528 | 5.781 |
| 184 | 0.674 | 0.808 | 0.947 | 1.090 | 1.238 | 1.390 | 1.546 | 1.707 | 1.873 | 2.043 | 2.217 | 2.396 | 2.580 | 2.768 | 2.960 | 3.157 | 3.359 | 3.565 | 3.776 | 3.991 | 4.211 | 4.435 | 4.664 | 4.898 | 5.136 | 5.379 | 5.626 |
| 186 | 0.632 | 0.763 | 0.899 | 1.039 | 1.183 | 1.332 | 1.485 | 1.643 | 1.805 | 1.971 | 2.142 | 2.317 | 2.496 | 2.680 | 2.869 | 3.061 | 3.259 | 3.460 | 3.666 | 3.877 | 4.092 | 4.311 | 4.536 | 4.764 | 4.997 | 5.235 | 5.477 |
| 188 | 0.591 | 0.719 | 0.852 | 0.989 | 1.131 | 1.276 | 1.426 | 1.580 | 1.739 | 1.901 | 2.068 | 2.240 | 2.415 | 2.595 | 2.780 | 2.968 | 3.161 | 3.359 | 3.561 | 3.767 | 3.977 | 4.192 | 4.411 | 4.635 | 4.863 | 5.095 | 5.332 |
| 190 | 0.551 | 0.677 | 0.807 | 0.941 | 1.079 | 1.222 | 1.369 | 1.520 | 1.675 | 1.834 | 1.998 | 2.165 | 2.337 | 2.513 | 2.694 | 2.879 | 3.067 | 3.261 | 3.458 | 3.660 | 3.866 | 4.076 | 4.291 | 4.509 | 4.732 | 4.960 | 5.192 |

1.9≤BRI<2.9　3.9≤BRI<4.0　4.0≤BRI<5.2　BRI≥5.2

Figure S14. Body roundness index chart for high risk CVDRFs identification among female

BRI cut-off by BMI catergory for high risk CVDRFs identification among female: underweight(BMI<18.5) BRI≥1.9; normal weight(18.5≤BMI<24) BRI≥2.9; overweight (24≤BMI<28) BRI≥4.0; Obese (BMI≥28) BRI≥5.2
